# Diversity Drivers of Inland Saline Vegetation—What Unites Them and Divides Them?

**DOI:** 10.1002/ece3.71249

**Published:** 2025-05-14

**Authors:** Zuzana Dítě, Róbert Šuvada, Tibor Tóth, Daniel Dítě

**Affiliations:** ^1^ Institute of Botany Plant Science and Biodiversity Center, Slovak Academy of Sciences Bratislava Slovakia; ^2^ Institute for Soil Sciences Centre for Agricultural Research, HUN‐REN Budapest Hungary

**Keywords:** azonal vegetation, biogeographical variation, climate‐based vegetation analysis, continental scale assessment, edge‐effect, halophytes, intrazonal vegetation

## Abstract

The current knowledge on vegetation of salt‐affected habitats has been advanced, calling for a supra‐regional assessment. We evaluate the common and distinct features of inland saline/alkaline landscapes of temperate Europe in terms of floristic composition, vegetation types, and abiotic conditions to find out what are the main drivers of their spatial variability and diversity. We delineate 13 subregions with a high occurrence of inland saline/alkaline habitats and by utilizing extensive field surveys in the past 20 years we analyze factors presumably affecting their variability: the size of the area, its proximity to the seacoast, and bioclimatic variables. We subjected them to descriptive statistics and ANOVA; principal components analysis was performed to reduce the number of dimensions for each dataset; correlation analysis was conducted to identify the statistical dependence between the diversity of subregions and observed factors. Despite the general uniformity typical for saline habitats, we observed that the subregions exhibit significant dissimilarity. Among the 107 autochtonous plant specialists, they have in common only one obligate and five facultative halophytes ( *Puccinellia distans* agg.; *Carex distans* , *Juncus gerardi* , *Lotus tenuis* , *Schoenoplectus lacustris* subsp. *glaucus* and *Trifolium fragiferum* ). The size of the subregion and its distance from the nearest seas did not affect the overall variability. Higher halophyte richness is driven by the broader range of abiotic and biotic prerequisites, especially the specific climate featuring summer evaporation causing various salinization levels in the soil, which is the most pronounced in the central subregions of the Pannonian Lowland. In its peripheries, the effect of specific conditions is lower, generating a reduced richness of halophytes, and in the subregions of the North German and Polish Plain and the Transylvanian Basin, the edaphic conditions (salt springs from salt deposits) take the main role, resulting also in a decreased halophyte richness and variability.

## Graphical abstract

We evaluated the drivers of spatial variability and halophyte diversity of inland saline habitats in 13 subregions in temperate Europe. Among the 107 native halophytic plant specialists, the 13 subregions have in common one obligate halophyte and five facultative halophytes. The leading position in the number of halophytes, plant communities, and specific climate for the development of saline/alkaline habitats has Alföld, the central part of the Pannonian Lowland. The size of the subregion and its distance from the nearest seas did not affect the overall variability; the high variability is driven by the broader range of abiotic and biotic prerequisites.

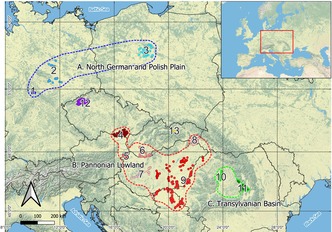

## 1 Introduction

Salt‐affected habitats are largely used as model ecosystems for displaying comprehensive patterns of varying affinities of species and plant communities to soil moisture and salinity.

(Adams 1963; Dijkema 1984; Bergmeier and Schaminée 2016). Plant life forms, physiognomy, and diversity in saline landscapes greatly differ among tropical, mediterranean, temperate, boreal, or arctic macroclimates (Chapman 1974), while a higher species richness is seen at medium latitudes in the temperate zone (Adam 1990). They are made up there from rushland‐grassland mosaics with prevailing hemicryptophytes, and in the lower mudflats with therophytes (Cantero et al. 2016; Leuschner and Ellenberg 2017). Although coastal and inland saline habitats are uniform and species‐poor in a certain climatic zone due to the extreme edaphic conditions (Lee and Kim 2018), their assembly of communities and zonation pattern greatly vary depending on the local soil properties (Sánchez et al. 1996; Szabó and Tóth 2011; Hulisz et al. 2016). The fragmented abiotic conditions cause isolation between the species formations, thereby causing a fragmented appearance of halophytic vegetation. Saline/alkaline habitats of the interior occur in internal drainage basins with a continental to subhumid climate where the dry periods promote high evaporation, causing increased salinity levels in the soil with various ionic compositions (Burchill and Kenkel 1991; Akhmedenov 2018; Pätsch et al. 2024), in some areas often associated with very high pH (Mezősi 2017).

The occurrence of saline habitats is restricted to the coastlines; in the interior, they develop only under particular climatic and/or edaphic conditions. In Europe, they have insular distribution mostly in the lowlands of the North European Plain (Lubińska‐Mielińska et al. 2023) and the Carpathian‐Pannonian bioregion (Borhidi et al. 2012; Eliáš jun. et al. 2013; Dítě et al. 2023). They are recognized in the Natura 2000 network as “Inland salt marshes and salt steppes 1530” and “Inland salt meadows 1340” according to the European Habitat directive (European Communities 1991) and they are also included on the European Red List of Habitats harboring rare endangered plant and animal species (Janssen et al. 2016). Although the evolution center of halotolerant phanerogams in Eurasia are the semi‐desert steppe regions and the warm lagoons of the Mediterranean (Acosta et al. 2007; Breckle and Wucherer 2012), these central European saline/alkaline habitats have other biogeographical importance; the disjunctive Eurasian range of halophytes *Artemisia rupestris* or *A. laciniata* underlies the relic character of inland salt‐affected vegetation (Wendelberger 1950). The significance of inland saline habitats as stepping stones in the postglacial migration routes of coastal and inland halophytes is also recognized in the phylogeographic studies (Weising and Freitag 2007).

As the westernmost enclave of the 9000‐km‐long Eurasian forest‐steppe belt, continental saline vegetation is in the transitional zone between temperate forests and steppes, featuring a complex mosaic of herbaceous and woody habitats (Bede‐Fazekas et al. 2023). Towards the south, inland saline/alkaline landscapes are typical in the basins of the Balkan Peninsula: in Serbia (Zlatković et al. 2019), North Macedonia (Micevski 1965) and Bulgaria (Tzonev et al. 2008), influenced by sub‐Mediterranean climate. To the east, through the Wallachian Plain and Bessarabia (Pop 2002), they reach their highest volume in the East European Plain (Golub et al. 2017; Dziuba and Dubyna 2021) to Central Asia (Inelova et al. 2024) as the part of the Eurasian steppe zonobiome with semi‐arid climate. Another European hotspot of inland saline habitats is the semi‐arid meseta of the Iberian Peninsula (Ladero et al. 1984; Salazar‐Mendías and Lendínez 2021) with pronounced Mediterranean character. In areas with exceeding precipitation over evapotranspiration in the summer period, spring‐fed, azonal saline vegetation has been formed (Westhus et al. 1997; Danihelka et al. 2022; Dítě, Šuvada, and Dítě 2023).

Not all of continental saline landscapes have received the same research intensity in Europe. Most scholarly publications are concentrated in the regions in Germany (e.g., Brandes 1999), Poland for example, (Piernik 2005) or the Czech Republic (Grulich 1987), while the saline vegetation of Romania has been surveyed recently (Dítě et al. 2021; Dítě et al. 2023). Only a few studies elaborate data extending across national boundaries, describing the vegetation variability along salinity, inundation, and microtopography gradients in temperate Europe (Eliáš jun. et al. 2013; Lubińska‐Mielińska et al. 2023). An exceptional biogeographical study was provided in the past (Wendelberger 1950), where the main territories of the European continental salt flora (inland Germany, the Great Hungarian Plain and Romania) are defined and compared, along with connections to the coastal salt marshes of the North Sea. This fundamental survey is the first and single vegetation synthesis that provides an exhaustive description of salt‐affected regions. After a not quite a century, the impact of rapid technical development and a growing human population in Europe abruptly transformed the biosphere. Water regulations, intensive agriculture, plowing of uncultivated grasslands, and the abandonment of traditional grazing have all led to the degradation of natural ecosystems, severely suffering from biological invasions and native species extinctions (Rogan and Lacher 2018).

In our study, the targeted habitats are continental saline/alkaline landscapes threatened by severe degradation processes and area decrease (Janssen et al. 2016). We conduct a supra‐regional synthesis of inland saline/alkaline habitats, and we outline their floristical relations to the nearest coastal salt marshes in temperate Europe. Along with their biogeographical differentiation, we aimed to find out what governs the spatial variability of inland saline habitats and which factors are decisive in their higher diversity?

## 2 Methods

### 2.1 Study Area

The study area, located in eastern central Europe, covers approximately 650,000 km^2^, stretching between 45.3° N–52.2° N latitude and 10.1° E–25.5° E longitude. It has a high proportion of rushlands, grasslands, and muflats shaped by extreme edaphic conditions (groundwater fluctuation and high soil salinity/sodicity), confined to flat lowlands or large basins (Leuschner and Ellenberg 2017) encompassing the vegetation classes of *Therosalicornietea*, *Crypsietea aculeatae*, and *Festuco‐Puccinellietea* (Dítě et al. 2023).

**FIGURE 1:**
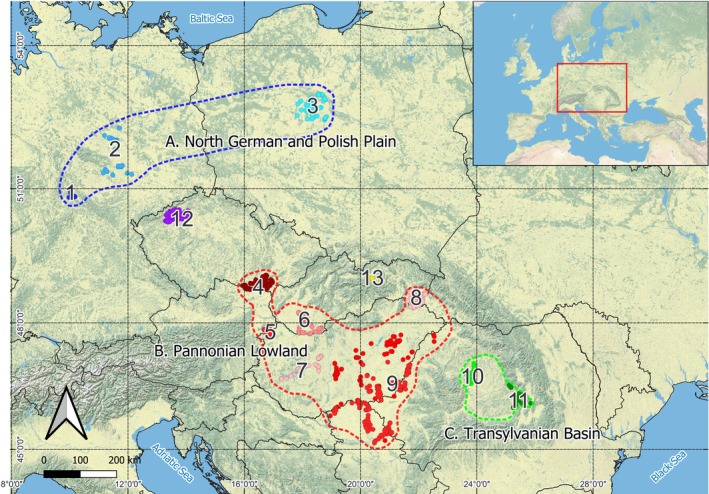
Study area showing the occurrence of inland saline/alkaline landscapes in eastern central Europe. The dots are documented localities of halophytic vegetation outlining the boundaries of subregions (numbered from 1 to13) subjected to assessment; the polygons with interrupted lines are the assessed macroregions.

Based on the literature involving saline/alkaline landscapes regardless of administrative borders, we defined three major territories of continental halophytic vegetation, in this study entitled as macroregions A, B, C; within them we delineated 11 areas and outside them two, all entitled as subregions (numbered from 1 to13). Each is marked on the map (Figure 1), and the ecoregional division is as follows:

- North German and Polish Plain: 1. Thüringen, 2. Sachsen‐Anhalt, 3. Kujawy.
- Pannonian Lowland: 4. Jižní Morava, 5. Seewinkel, 6. Podunajská nížina, 7. Dunántúl, 8. Východoslovenská nížina, 9. Alföld.
- Transylvanian Basin: 10. Câmpia Transilvaniei, 11. Harghita.

12. Mostecká pánev, 13. Spiš.

All macro‐and subregions belong to the Pannonian and the Continental biogeographical regions according to the European Environment Agency (https://www.eea.europa.eu/data‐and‐maps/figures/biogeographical‐regions‐in‐europe‐2), with the exception of Spiš, recognized in the Alpine biogeographical region.

The study area is situated in a moderate climate zone with four seasons and year‐round precipitation. According to Köppen‐Geiger climate types (Peel et al. 2007) it belongs to temperate (“D”) and continental (“C”) climate groups, without a dry season (“f”), and the level of heat in summers is hot (“a”) to warm (“b”). On the global high‐resolution (1 km) classification map by Beck et al. (2023) the four identified climate types Cfa, Cfb, Dfa, Dfb show overlap among the subregions. Further climatic differences among the subregions we evaluate by using bioclimatic variables defined by Fick and Hijmans (2017); more details about the analysis are in data processing section below.

**A. North German and Polish Plain**

On the eastern part of the North European Plain, there are two continuous lowlands: the North German Plain and the Polish Plain (Woronko and Dąbski 2022), extending from the North and Baltic seas southward to the Central German Uplands. They were formed during various glacial advances of terrestrial Scandinavian ice sheets and periglacial geomorphologic processes in the Pleistocene era. Due to the mild climate and fertile soils of the plain, the transformation of the landscape is significant. Inland salt marshes and meadows occur on a loess belt stretching along the southeastern rim of the North German Plain and in central Poland (Piernik 2012). They were traditionally included in the coastal habitats of the *Puccinellion maritimae* and *Armerion maritimae* alliances (Bergmeier 2020). The relation with the inland salt marshes has been recently investigated (Dítě et al. 2022). Salt‐affected soils are attributed to the salinization of the groundwater, where salts originate from the relict seawater of the Zechstein sedimentary rock layers deposited after a series of transgressions and regressions during the Upper Permian period. The original distribution of salts has changed due to regional tectonics taking place from the Triassic to the Early Cenozoic, resulting in the formation of different salt structures like pillows and diapirs (Balaske 2012; Zhang et al. 2013). A great extent of saline soils is the result of industrial mining (salt/potash/soda) and the products of their processing (Braukmann and Böhme 2011; Pindral et al. 2023). Potash‐mine dumps and their vicinity, affected by secondary salinization of the groundwater, have been colonized by halo‐tolerant vegetation (Garve and Garve 2000). The long history of salt mining and the water regulations beginning already from the 17th century do complicate the unambiguous distinction of natural and secondary saline habitats.

**B. Pannonian Lowland**

It forms a topographically discrete basin surrounded by obvious geographic barriers: the Carpathian arc, the Alps, the Dinarides, and the Balkan Mountains (Hoffman and Davies 1983). During the Pliocene epoch, there were deposited 3–4 km of fine‐grained sediments when the shallow Pannonian Sea reached its greatest extent. This was followed by an uplift on the margins, leaving an inland lake (today's Alföld), which dried up and was filled with a thick layer of riverine deposits from the surrounding uplifted highlands and eolian sediments (Fodor et al. 1999; Nemčok et al. 2006). The Neogene basin spans approximately 600 km from east to west and 500 km from north to south. The deposited silt into the loess‐silt surface depressions resulted in sodification processes with predominating alkaline salts (NaHCO_3_, Na_2_SO_4_, MgSO_4_) and salinization induced by NaCl (Pécsi 1970). Hydrological conditions of the Pannonian Lowland were shaped by the changes in the course of the largest rivers (Danube and Tisza). Up to the massive river regulations before the mid‐19th century, the lowland was a continuous marsh with open water (Mezősi 2017). Some areas of saline/alkaline habitats on the edges of the former flood plains have therefore secondary origins, but ancient salt steppes from the Würm glacial stage have been also revealed (Sümegi 2011).

**C. Transylvanian Basin**

It is a major sedimentary basin located in the eastern part of the European Alpine orogenic system. It has a roughly circular shape surrounded by the internal Eastern and Southern Carpathians. Its sedimentary fill (5 to 8 km thick) is comprised of Upper Cretaceous to upper Miocene deposits (Krézsek and Bally 2006). One of the most important factors influencing the late stage of the basin is related to the Late Miocene uplift of the Carpathians (Sanders et al. 2002) associated with gravitational spreading of the post‐salt overburden strata. For the rich NaCl diapirs found there, the scientific study of salt tectonics became established right in the Transylvanian Basin (Jackson 1997). Inland saline environments, including salt ponds and salt marshes near the emerging springs, are characteristic features of the Transylvanian landscape (Simulescu and Grigoraş 2016). The appearance of salt led to mining and/or evolution of surface pseudokarst, resulting in numerous artificial (anthroposaline) or natural (karstosaline) lakes (Alexe et al. 2018).

Appendix S1 contains the main features of the subregions in terms of physical geography, landscape characteristics, and research history.

### 2.2 Conceptualization of the Study

The above named macro‐ and subregions with a high occurrence of continental halophytic vegetation were subjected to a detailed biogeographical assessment in the context of temperate Europe, where we analyze their common and distinct features in terms of floristic and vegetation composition concerning also their relations to the seacoasts and climatic conditions. We selected several attributes which presumably affect the diversity and spatial variability of inland saline/alkaline habitats:

*Size* of a discrete area (in this study designated as subregions from 1 to 13 listed above). We assume that a larger area has a higher number of salt‐adapted species;

*Proximity* of the subregion to the nearest seacoast: the geographical distribution of each halophyte is different, which may reveal several phytogeographical patterns depending on the relations of the species to a particular coastal region;

*Bioclimatic variables*: different latitude/longitude and topography of a subregion manifest different bioclimatic conditions which may have an important role in shaping the floristic composition and variability of inland saline habitats.

Under the term “diversity” in this assessment we mean the representation of plant specialists (obligate and facultative halophytes) in the particular subregions, and under “spatial variability” the occurrence of halophytes in various vegetation types (plant communities).

We evaluate two different aspects of diversity: “species composition” refers to particular halophytes occurring in the subregions (not count) and “halophytes richness” which refers to the number of occurring halophytes in the subregions.

To find out the drivers contributing to high halophyte richness, we tested its dependence on the following factors: species composition, vegetation types, climatic characteristics, subregion size and proximity to the closest sea.

### 2.3 Data Collection and Data Processing

Distribution data of halophytes (in this study salt‐adapted vascular plants living in terrestrial environments) and their vegetation types at low syntaxon unit (associations) constitute several data sources:

- Own data obtained during a series of phytosociological surveys from 2003 to 2022 across central Europe, which we store in the TURBOVEG database (Hennekens and Schaminée 2001)
- Regional/local floristic and vegetation studies conducted within the North German and Polish Plain Pannonian Lowland, and Transylvanian Basin. Due to the large amount, we include here only the most important: for Germany and Poland, for example, Wilkoń‐Michalska (1963), Westhus et al. (1997), Frank et al. (2004), John and Stolle (2011), (Hartenauer et al. 2012) and Kaźmierczakowa et al. (2014); for Austria, Czech Republic, Slovakia, Hungary, and Serbia, for example, Wendelberger (1943), Vicherek (1973), Toman (1976), Király (2009), Borhidi et al. (2012), Bartha et al. (2015); for Romania, for example, Soó (1947), Sârbu et al. (2013) and Grigore and Cojocariu (2020). For the distribution of coastal species outside our study area, we used global distribution maps by Meusel and Jäger (1992).
- Personal consultations with local florists (their names are given in the Acknowledgements).

Halophytes with historical occurrence or regionally extinct (based on the literature) were also included in the assessment.

The classification of plant specialists according to their salt tolerance follows the habitat‐based concept of Dítě et al. (2023), where obligate halophytes occur exclusively in saline habitats, and facultative halophytes occur in at least one non‐saline habitat in their central European area.

The taxonomy and nomenclature of the plant species follows Euro+Med (2018); Species in taxonomically difficult groups were aggregated: *Salicornia europaea* (incl. *S. appressa*) and *Puccinellia distans* agg. (incl. *P. distans* , *P* . *limosa* , *P. peisonis*). Nomenclature of the higher syntaxa is consistent with the EuroVegChecklist (Mucina et al. 2016); association names follow Borhidi et al. (2012); Dítě et al. (2014, 2021, 2022, 2023).

#### 2.3.1 Occurrence Data of Species and Vegetation Types

The datasets on the occurrence of halophytic plants and associations were compiled to create the matrix tables for principal components analysis (PCA) and correlation analysis. Each species and association is characterized there as ordinal variables based on their frequency (rate of occurrence) in the subregions on the following scale: 0—no occurrence; 1—a very rare occurrence; 2—scattered occurrence, in less than half of the locations; 3—a common occurrence, in most locations. Analytic tables carrying the data on the occurrence of halophytes and associations were used to create a series of tables showing the similarity in terms of mutual and unique halophytes and vegetation types in the individual macro‐ and subregions (Appendix S4).

To all locations, we assigned coordinates in the WGS84 coordinate system and the attribute of belonging to specific macroregions (A, B, C) and subregions (1–13). These points were used for calculating climate data, subregion size, and proximity to the sea coasts.

#### 2.3.2 Climate Data

For all occurrence data assigned to subregions, we calculated 19 bioclimatic variable values (BIO1‐BIO19) defined by Fick and Hijmans (2017) based on the geographical location of the subregions. The data source was obtained from WorldClim2 raster layers with 2.5‐min spatial resolutions and further evaluated by descriptive statistics; results are shown in Appendix S2. We used the ANOVA test and the Unequal N HSD test (alpha = 0.05) to assess whether the groups presented statistically significant differences; then all bioclimatic values are displayed by boxplots.

The *size* of each subregion was determined based on the area of the polygon generated from point occurrence data matching with the particular subregion. Each of these polygons was created by dissolving all the subregion surfaces made by the *Delaunay triangulation* tool in QGIS software.

The *proximity* to the seacoast was calculated as the shortest distance from the polygon centroids of each subregion to the coastline of the Baltic, Black, and Adriatic seas. We created a four‐point scale for the distribution data of halophytes to what extent they are confined to the interior and coasts, based on similar studies in the literature: C0—exclusively inland species, C1—species with common inland occurrence, very rare on coasts, C2—species growing both on coast and inland, C3—coastal species, very rare inland. The *Centroids* tool was used for identifying the centroid, and the *Point to line distances* tool for the shortest distance. Coastlines were obtained from the shapefile *Coastlines*, which is part of Map data from OpenStreetMap. Three quantitative variables, which represent the minimum distance (km) from subregions centroids to the coastline of the Baltic, Black, and Adriatic seas, were used as a source for principal component analysis (PCdiss) and are listed in Appendix S4.

#### 2.3.3 Principal Components Analysis

For the compiled datasets (species, vegetation types, climate and proximity to seas) described above, we calculated ordination scores for each subregion on the first axes using Principal components analysis (PCA, variance–covariance matrix, see Appendix S4 _#2 Ordination scores+rank level). We reduced the total number of dimensions of each dataset to one that includes the highest variability to evaluate the dependence of “halophyte richness” on the observed factors. Hereafter, we refer to the calculated values as PCspec (halophytes composition), PCassoc (vegetation composition), PCclime (climatic values), and PCdiss (geographical distance from the seas), and the 3D scatter plot was created to graphically display the similarity among the subregions using the three ordination values (PCspec, PCassoc, PCclime) of the highest correlation with the number of halophytes in the subregions.

#### 2.3.4 Correlation Analysis

For a normal distribution of data and a unified type of variables for subsequent correlation analysis, we transformed all analyzed variables to the rank level. This data transformation ensured a normal distribution for all factors that were tested by the Shapiro–Wilk test, and the influence of outlier cases was excluded. The results are shown in the table in Appendix S4 #4_Ordination scores+rank level, and their transformation is displayed by barchart plots in Appendix S3. After this adjustment, all variables were used to evaluate the statistical dependence between “halophyte richness” of subregions and observed factors by Pearson correlation analysis and the test of its statistical significance. Their dependence is shown by the regression line supplemented with coefficients of determination (*r* ^2^) and statistical significance of correlation (P).

All analyses were carried out in Statistica 7 (StatSoft Inc. 2004) and PAST 4.05 (Hammer et al. 2001). The maps were created using the program QGIS, version 3.22 (QGIS Development Team 2022) with a background layer from World Physical Map (source US National Park Service).

## 3 Results

### 3.1 Species Composition and Diversity

The entire study area carries 46 obligate and 61 facultative halophytes. The richest subregion in halophytes is Alföld with 28 obligate and 61 facultative halophytes (Table 1) followed by the western subregions of the Pannonian Lowland: Dunántúl, Seewinkel, Podunajská nížina, and Câmpia Transilvaniei (belongs to the Transilvanian Basin). The marginal subregions of the Pannonian Lowland (Jižná Morava and Východoslovenská nížina) came up with a reduced number of halophytes, and a lower number of halophytes are hosted by the subregions of the North German and Polish Plain, among them Sachsen‐Anhalt is the richest. Three subregions have less than a third of the entire matrix of halophytes (Table 1).

**TABLE 1:** Number of halophytes and their proportion according to their category of salt tolerance (sensu Dítě, et al. 2023) in 13 subregions of inland saline/alkaline habitats of eastern central Europe.

Subregion name	Total no. of halophytes	Obligate halophytes	Facultative halophytes
1. Thüringen	24	10	14
2. Sachsen‐Anhalt	45	20	25
3. Kujawy	20	6	14
4. Jižní Morava	46	11	35
5. Seewinkel	68	25	43
6. Podunajská nížina	65	19	46
7. Dunántúl	74	26	48
8. Východoslovenská nížina	37	10	27
9. Alföld	89	28	61
10. Câmpia Transilvaniei	65	25	40
11. Harghita	32	11	21
12. Mostecká pánev	31	6	25
13. Spiš	13	2	11

**FIGURE 2:**
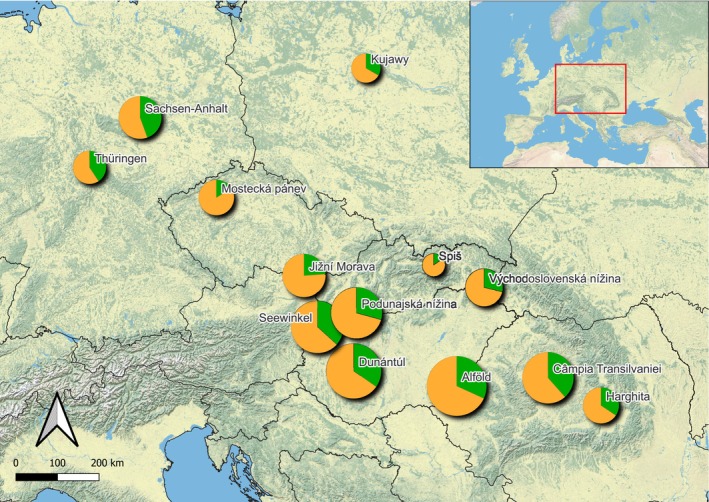
Proportion of obligate (green) and facultative halophytes (orange) among the 13 subregions of inland saline/alkaline habitats. The categories of salt tolerance are sensu Dítě et al. (2023). The size of the circles represents the total number of halophytic species in the subregion.

Facultative halophytes prevail everywhere throughout the study area (Figure 2) with various distribution patterns among the subregions (56%–85%). The highest proportion of obligate halophytes is in Sachsen‐Anhalt (44%), Thüringen (42%), Câmpia Transilvaniei (38%) and Seewinkel (37%). The isolated regions of Spiš and Mostecká pánev have the lowest proportion of obligate halophytes (Figure 2).

From the overall 46 obligate halophytes, only a single species is common for all subregions: *Puccinellia distans* agg. If we exclude the two isolated subregions, *Tripolium pannonicum* is the second obligate halophyte shared by each (Appendix S4 _#7 Mutual and unique species).

When merging the subregions into the larger macroregions, their number increases by five more: *Atriplex litoralis*, *Bupleurum tenuissinum*, *Glaux maritima* , *Oxybasis chenopodioides*, and *Spergularia marina* (Appendix S4 _#1 Source PCspec).

From the 61 facultative halophytes, there are only five species that occur in each subregion: *Carex distans* , *Juncus gerardi* , *Lotus tenuis* , *Schoenoplectus lacustris* subsp. *glaucus*, and *Trifolium fragiferum* . On a macroregional scale (without the isolated regions Spiš and Mostecká pánev) this number greatly increases: *Althaea officinalis* , *Atriplex prostrata* , *Carex hordeistichos* , *C*. *secalina*, *Centaurium littorale* subsp. *compressum*, *Cerastium dubium* , *Juncus ranarius* , *Matricaria chamomilla* , *Melilotus dentatus*, *Mentha pulegium* , *Peucedanum officinale* , *Pulicaria dysentherica*, *Rumex stenophyllus* , *Scorzonera parviflora*, *Trifolium striatum* , and *Triglochin maritima* (Appendix S4 _#1 Source PCspec). Alföld holds all facultative halophytes occurring in the entire study area.

**FIGURE 3:**
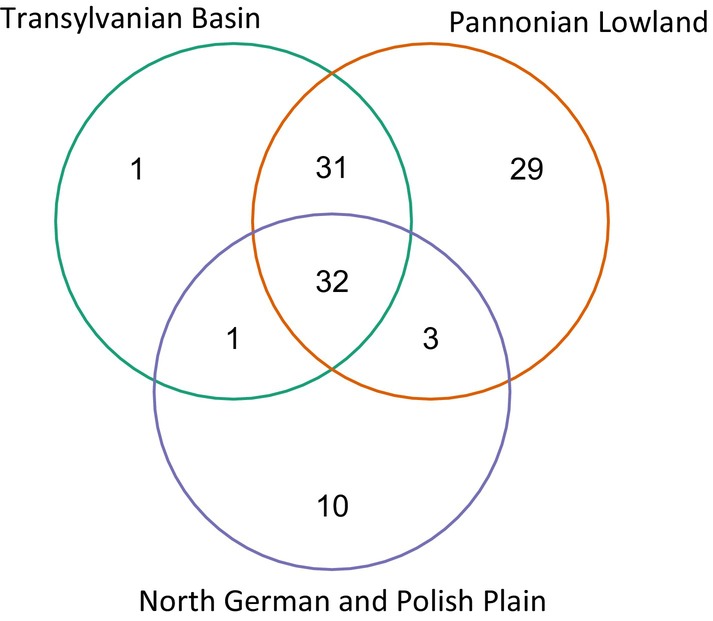
The number of shared halophytes (obligate and facultative) among the macroregions in eastern central Europe.

All the three macroregions share together 32 halophytes (Figure 3). The Pannonian Lowland and Transylvanian Basin have the most shared (63), from which there are 23 obligate halophytes, and 11 of them in the North German and Polish Plain are missing (*Artemisia santonicum*, *Camphorosma annua*, *Crypsis aculeata* , *Galatella sedifolia*, *Petrosimonia triandra*, *Peucedanum latifolium*, *Plantago schwarzenbergiana*, *Plantago tenuiflora*, *Rorippa sylvestris* subsp. *kerneri*, *Suaeda prostrata* , *S. salsa*). These are mainly species of Irano–Turanic distribution.

Still on a macroregional scale, the most unique species in the group of both obligate and facultative halophytes are in the Pannonian Lowland, with 29 species, for example, *Lepidium cartilagineum*, *Juncus maritimus* , *Pholiurus pannonicus*, *Schoenoplectus litoralis* , and *Suaeda pannonica*; then *Beckmannia eruciformis, Galatella cana*, *Lepidium perfoliatum* , *Lythrum tribracteatum* , *Sedobassia sedoides*, *Schoenoplectus pungens* , *Silene multiflora, Trifolium strictum*, and *Cirsium brachycephalum* (Appendix S4 _#7 Mutual and unique species).

The North German and Polish Plain has a unique occurrence of 10 obligate halophytes (e.g., *Apium graveolens* and *Hornungia procumbens* ) and the Transylvanian Basin has only one, *Plantago cornuti* (Figure 3, or Appendix S4 _#7 Mutual and unique species).

Among the subregions, the highest number of unique obligate halophytes has Sachsen‐Anhalt. Six species occur exclusively here: *Artemisia maritima* , *A. rupestris* , *Blysmopsis rufa* , *Eleocharis parvula* , *Halimione pedunculata*, and *Plantago coronopus* . Two unique obligate halophytes have Alföld ( *Lotus angustissimus* , *Salsola soda* ) and Seewinkel ( *Carex extensa* , *Linum maritimum*). Regarding facultative halophytes, Alföld is the single subregion with unique occurrence of *Gagea szovitsii*, *Galatella villosa*, *Rumex pseudonatronatus* , *Trifolium micranthum* , *T. ornithopodioides* , and *T. subterraneum* .

The endemism in the salt flora of eastern central Europe is low; only on a macroregional scale was confirmed one obligate halophyte (*Suaeda pannonica*) and two facultative halophytes (*Armoracia macrocarpa*, *Cirsium brachycephalum*), each growing in the Pannonian Lowland.

### 3.2 Floristic Relations With Seacoasts

**FIGURE 4:**
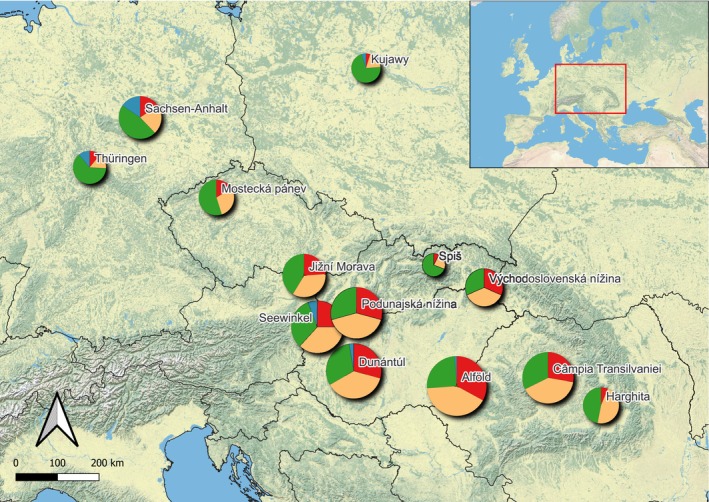
The proportion of halophytes in 13 subregions of inland saline habitats based on their connection to the seacoast and interior: 0/red—exclusively inland species, 1/orange—species with common inland occurrence, very rare on coasts, 2/green—species growing both on coast and inland, 3/blue—coastal species, very rare inland. The size of the circles represents the total number of species in the subregion.

The aspect *coasts* analyses the floristic relation of halophytes of the interior with the coastal salt marshes. The third of the salt flora of the study area (31 species) has strictly inland distribution, not growing on any sea coast. Their highest number was detected in the Pannonian subregions, for example, in Alföld, we confirmed 29 species (Appendix S4 _#5 Source Fig4). In the other two macroregions, the proportion of coastal halophytes is higher (Figure 4).

**FIGURE 5:**
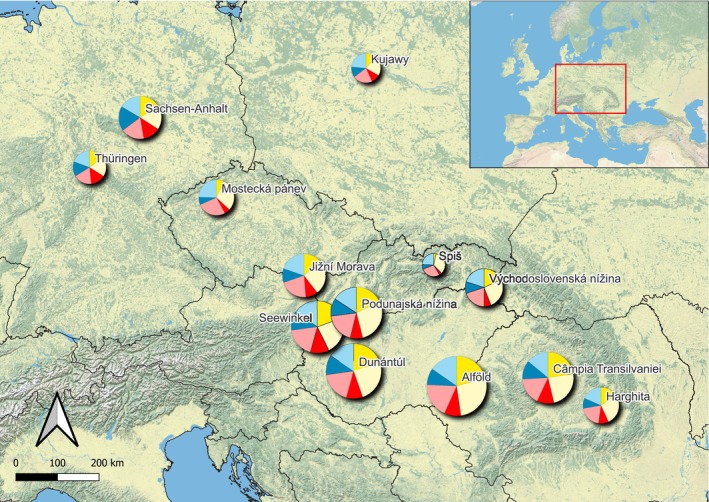
Connection of halophytes to different seacoasts in 13 subregions of inland saline habitats. Baltic Sea coast: Dark blue—obligate halophytes, light blue—facultative halophytes. Adriatic Sea coast: Dark red—obligate halophytes, light red—facultative halophytes. Black Sea coast: Dark yellow—obligate halophytes, light yellow—facultative halophytes. The size of the circles represents the total number of species in the subregion.

From the 107 halophytes, there are 76 species that, beyond their inland distribution, are also known on the coasts of the three closest seas: Adriatic Sea (belonging to the Mediterranean Sea), the Black Sea, and the Baltic Sea (belonging to the North Sea) (Appendix S4 _#6 Source Fig5); 26 of them are reported from each coast.

There are 31 halophytes known from exclusively one of the seacoasts: two species on the Adriatic, seven on the Baltic seacoast, and 22 halophytes have coastal distribution only at the Black Sea coast. These Pontic and Irano‐turanic species occur in the Pannonian Lowland and Transylvanian Basin, almost half of them in Alföld and Câmpia Transilvaniei subregions. Towards the west, their proportion is decreasing (Figure 5) in favor of species distributed on the Baltic coast. In Mostecká Pánev and in the three subregions of the North German and Polish Plain, the proportion of species from the three sea coasts is approximately the same (Appendix S4 _#6 Source Fig5). Thus, the salt flora of inland Germany and Poland has a southward flow from the Atlantic coast across the North German lowlands, while the representation of Mediterranean salt‐adapted species is also noteworthy. The salt flora of the Pannonian region has a strong westward flow from the Black Sea coast along the Danube river and its tributaries. This confirms Wendelberger's theory about plant migration along the rivers into the foothills of the remote valleys, for example, Jižná Morava, the most distant appendix of the Pannonian salt vegetation (Wendelberger 1950). The low representation of species from the Adriatic coast is explained by the geographical obstacles (Dinarides) and the absence of large river flows in that direction.

### 3.3 Vegetation Types

**FIGURE 6:**
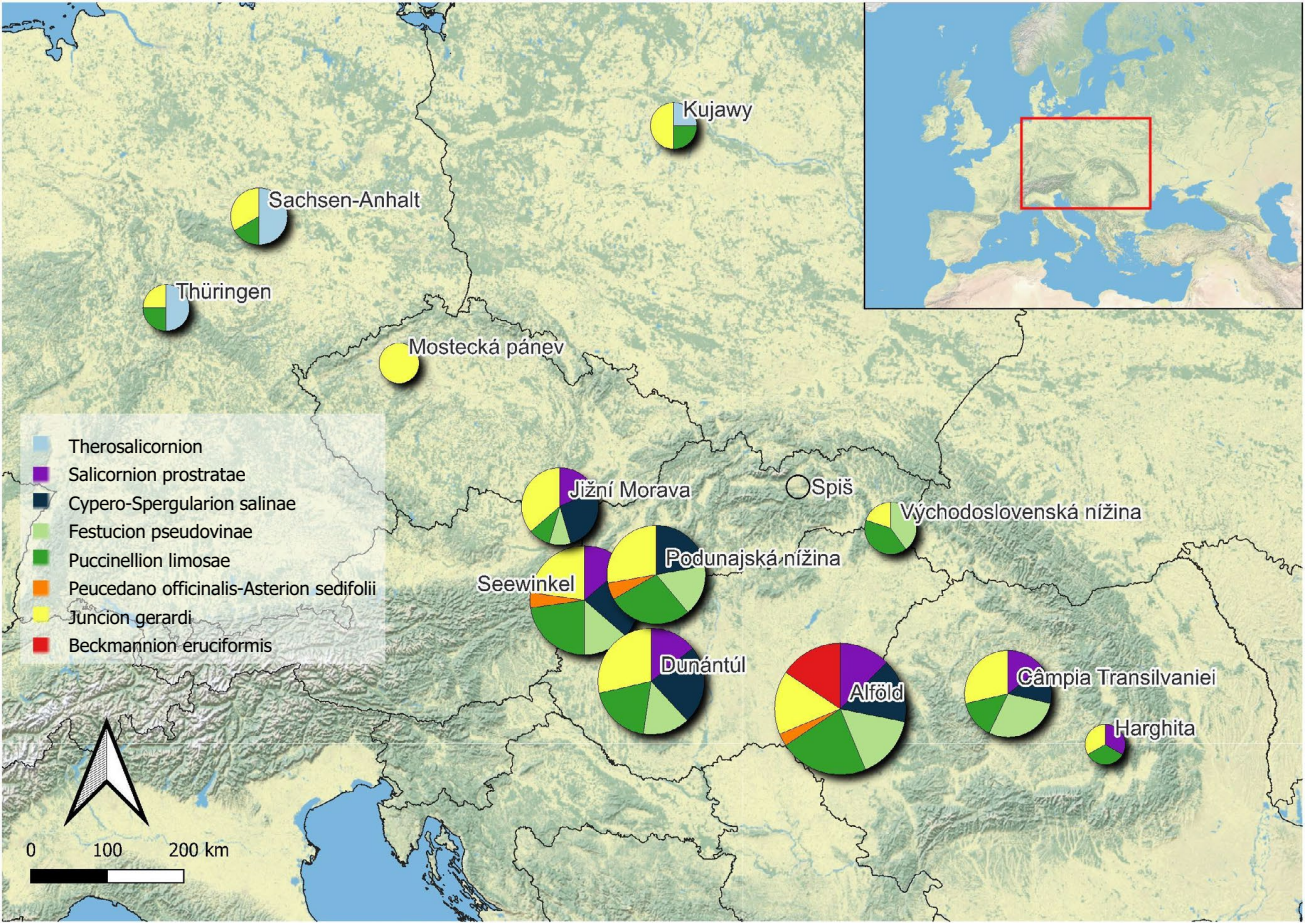
Proportion of vegetation types assigned to alliances in 13 subregions of inland saline habitats. The size of the circles represents the total number of associations in the subregion.

The vegetation of inland saline habitats in central Europe consists of three syntaxonomic classes, five orders, eight alliances, and 38 associations (Appendix S4 _#1 Source PCasoc). Comparing the subregions, except for the isolated Spiš region, only one alliance is common for all: *Juncion gerardi*. When we exclude the second isolated subregion Mostecká pánev, the other mutual alliance is *Puccinellion limosae* (Figure 6).

The highest vegetation diversity has the Pannonian Lowland, with various patterns among the individual subregions. Alföld has the highest number of alliances (seven) and only it has a unique alliance (*Beckmannion eruciformis*). The lowest vegetation variability, with three alliances, has Východoslovenská nížina, Harghita, Spiš, Mostecká pánev, Thüringen, and Sachsen‐Anhalt (Figure 6). On macroregional scale, the highest number of shared alliances (five) is between the Pannonian Lowland and Transylvanian Basin; both share two alliances with the North German and Polish Plain (Appendix S4 _#8 Mutual and unique vegetation).

On the lower syntaxon unit, there is not any mutual association for all the 13 subregions (Appendix S4 _#8 Mutual and unique vegetation); only when we exclude Mostecká pánev and Spiš, then it is *Puccinellietum limosae*. Except for Thüringen and Východoslovenská nížina, one more association is shared: the *Scorzonero parviflorae*‐*Juncetum gerardii*. The subregions of the North German and Polish Plain share one association, *Triglochino maritimae*‐*Glaucetum maritimae*. The highest number of mutual associations has Pannonian Lowland with Transylvanian Basin (Appendix S4 _#8 Mutual and unique vegetation).

There are many associations limited to only one subregion. In Sachsen‐Anhalt it is *Halimioni pedunculatae*–*Puccinellietum distantis*, in Dunántúl *Astereto pannonici*–*Schoenetum nigricanti*, in Câmpia Transylvanei *Artemisio*–*Petrosimonietum triandrae* and *Plantagineto cornuti*–*Agrostetum stoloniferae*. In Alföld, there are seven unique associations, not occurring elsewhere within the study area: *Salsoletum sodae*, *Bassietum sedoidis*, and the five associations of the *Beckmannion eruciformis* alliance.

### 3.4 Bioclimatic Variables

In each subregion, the warmest month is July, while Alföld features the highest monthly mean temperature of 20.5°C and a maximum 27.5°C (Appendix S2, boxplot BIO5, BIO10).

The mean annual air temperature (BIO1) is the highest in Dunántúl and Alföld at 10.6°C; the lowest is Harghita at 6.3°C. High seasonality and annual range of temperature (BIO4, BIO7) are also typical of the subregions of the Pannonian Lowland and Transylvanian Basin; noteworthy is Harghita with the highest seasonality of precipitation (BIO15). The annual precipitation (BIO12) in the whole study area varies between 510 and 690 mm; the lowest mean value is documented in Sachsen Anhalt, and the highest in Thüringen; smaller variance was found among the Pannonian subregions (Table 1 in Appendix S2). The rainfall is distributed regularly throughout the year only in Thüringen, where it significantly exceeds in the driest and coldest quarter of the year, too (boxplots BIO15, BIO17 and BIO19).

The analysis of all bioclimatic factors confirms a significant distinction of the central Pannonian subregions, especially Alföld, being the most outstanding in terms of mean annual temperature BIO1, maximum temperature of the warmest month BIO5, minimum temperature of the coldest month BIO6, temperature annual range BIO7, mean temperature of the driest quarter BIO9, and coldest quarter BIO11 (see boxplots and Table of Statistically significant differences among groups using ANOVA and the Unequal N HSD test in Appendix S2).

### 3.5 Similarity and Drivers Shaping Halophytes Diversity

**FIGURE 7:**
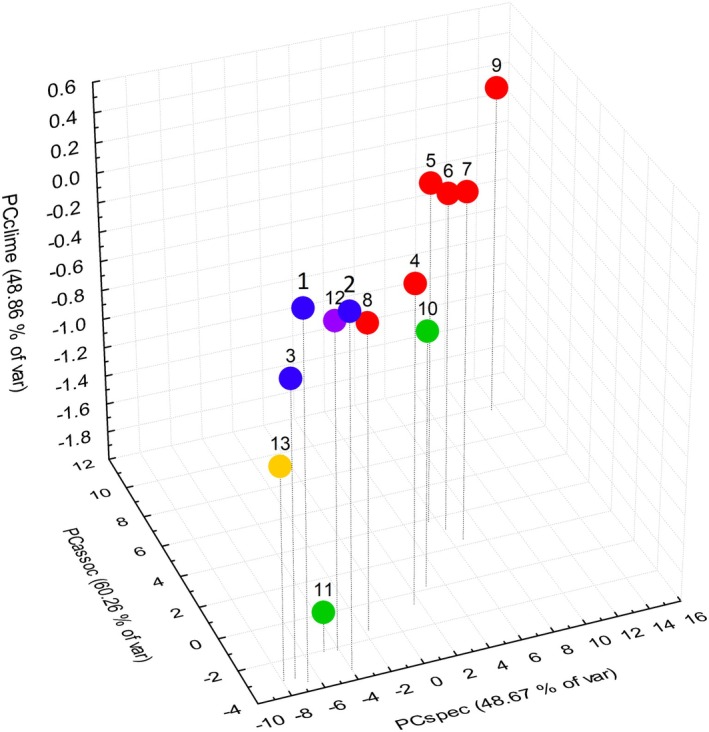
3D scatter plot displaying the similarity among the 13 subregions of central European saline habitats with three ordination values obtained by using principal components analysis; PCspec—first axis of species composition, PCassoc—first axis of vegetation types on association rank, PCclime—first axis of climatic values with logarithmic transformation. Macroregion North German and Polish Plain (blue): 1. Thüringen, 2. Sachsen‐Anhalt, 3. Kujawy. Macroregion Pannonian Lowland (red): 4. Jižní Morava, 5. Seewinkel, 6. Podunajská nížina, 7. Dunántúl, 8. Východoslovenská nížina, 9. Alföld. Macroregion Transylvanian Basin (green): 10. Câmpia Transilvaniei, 11. Harghita. Isolated subregions: 12. Mostecká pánev (violet), 13. Spiš (yellow).

The overall similarity of subregions according to multiple evaluated factors (halophytes composition, vegetation types and climate) underlines the outstanding position of Alföld (Figure 7). The most related subregions to Alföld are Seewinkel, Podunajská nížina, and Dunántúl. Harghita and Spiš are the most distinct, showing the lowest similarity to the other subregions. The macroregion Transylvanian Basin is split, as Câmpia Transilvaniei is more related to the Pannonian group in terms of vegetation and species composition than to the geographically closer, but climatically distinct Harghita.

**FIGURE 8:**
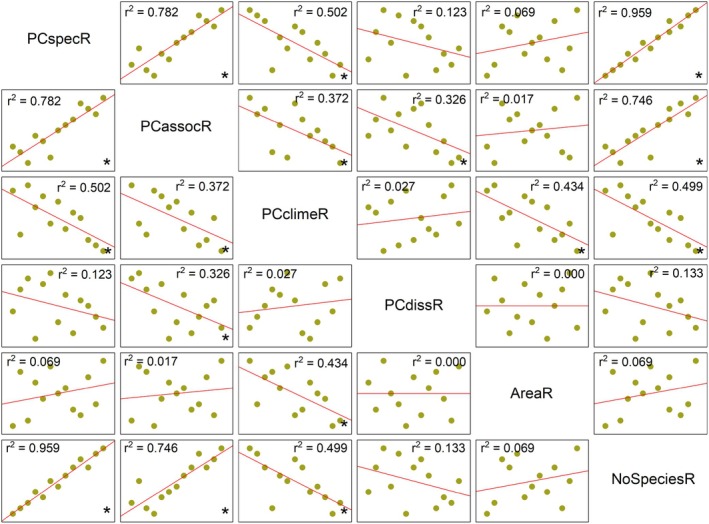
Matrix plot of Pearson correlation between the species diversity (NoSpeciesR) of subregions and the observed variables, transformed to the rank level; PCspecR (species composition), PCassocR (vegetation composition), PCclimeR (climatic values), PCdissR (geographical distance from the seas) and AreaR (subregion size). The regression line, supplemented with coefficients of determination (*r* ^2^) and statistical significance of correlation (*p < 0.05), is displayed.

The size of the subregion and its distance from the nearest seas did not affect the halophyte richness (Figure 8). It primarily correlated with the species composition, vegetation types, and the climatic characteristics.

## 4 Discussion

Despite the physiognomical uniformity typical for both inland and coastal saline landscapes (Silvestri et al. 2005; Eallonardo jr and Leopold 2014; Eliáš jun. et al. 2013; Lee and Kim 2018) we found that inland territories of salt‐affected habitats in temperate Europe are more distinct than similar in terms of floristic and vegetation composition. On a relatively small study area (650,000 km^2^), there occur altogether 107 autochthonous salt‐adapted plants. From them, the assessed subregions have in common only one obligate and seven facultative halophytes. The subregion Alföld came out as the most outstanding subregion, having the highest halophyte richness, unique halophytic species, and plant communities. Its variability did not depend on the size nor its distance from the nearest seacoast (Figure 8), but the decisive factor for a richer halophytic flora is the variety of opportunities for its existence.

Along with the crucial edaphic conditions (the presence of salt strata), the variability of inland saline/alkaline habitats is driven by climatically suitable conditions, such as the high‐temperature seasonality, maximum temperature of the warmest month, mean temperature of the warmest quarter, precipitation of the wettest quarter, and warmest quarter (see Appendix S2, boxplots, BIO4, BIO5, BIO10, BIO16, BIO18). These specific bioclimatic characteristics supporting diverse halophytic vegetation are the most pronounced in the Pannonian subregions, which is indicated by the high abundance of steppic species in those territories (e.g., *Artemisia santonicum*, *Camphorosma annua*, *Petrosimonia triandra*), many of them reaching their westernmost distribution range. From this viewpoint, saline/alkaline vegetation of the classes *Festuco‐Puccinellietea* and *Crypsietea aculeatae* is regarded as intrazonal vegetation of the forest‐steppe belt, developing in azonal habitat conditions, but showing affiliation to a particular zonal macrohabitat (Mucina et al. 2016). In these climatically conditioned saline habitats, the (semi)arid/subhumid climate in the growing season and the high summer temperatures support a negative water balance in the soil, one of the essential prerequisites for halomorphic soils. Shallow stagnant saline groundwater in flat sedimentary landscapes and its wide fluctuations due to capillary movements in the soil induced by drought contribute to increased alkalinity as well (Jobbágy et al. 2017; Szendrei et al. 2014).

The leading position of Alföld subregion in spatial variability and halophyte richness is also a result of habitat continuity ensured by suitable land use. Inland saline/alkaline vegetation is regarded as a semi‐natural habitat where the most appropriate is the non‐intensive grazing by mixed herd (Török et al. 2018). A high number of specialists in other grasslands is also supported by habitat continuity, for example, the regular mowing of hay meadows, as the most appropriate utilization, confirmed by several case studies (e.g., Raduła et al. 2022). These anthropogenic circumstances should not be omitted in the interpretation of the diversity of saline habitats, but due to the high variation in land use from site to site, the evaluation of such attributes on a macro‐regional scale is unfeasible.

The influence of specific climate is less pronounced in the case of the azonal saline vegetation, as the abiotic conditions originate mainly from the groundwater movements. In the precipitation‐richer subregions (Thüringen, Mostecká pánev or Harghita), the saline habitats are limited in spatial extent and their zonation is more simple. Therefore, a set of other, mostly topographical prerequisites contribute to their variability: groundwater rich in soluble salts originating from fossil salt deposits, endorheic basins, or compact, trampled soils with lack of air. In the case of Thüringen, as the rainiest subregion of the study area (Appendix S2, boxplots BIO12, BIO14, BIO17, BIO19), a lower evaporation in the growing season suggests a secondary origin of saline habitats there, strongly dependent on human activities such as soda/potash production and their disposal on slag heaps, although greater rainfall might raise the soil salinity through rising saline groundwater level.

The combination of edaphic factors with a pronounced anthropogenic influence therefore plays a principal role in shaping continental saline habitats´ variability. In the Pannonian Lowland, this human intervention is the drainage of permanent wetlands and former river regulations, which contributed to the development of secondary saline habitats there (see Appendix S1, or Tóth 2010b). The opposite process is also apparent; drainage of waterlogged saline areas caused groundwater sinking, withdrawal of sodication processes (leaching) in the soil, and lowered salinity, which strongly affects the recent plant composition documented in several Pannonian regions (Rakonczai et al. 2008; Dítě et al. 2015; Ladányi et al. 2023) and also in the North German and Polish Plain (Bosiacka et al. 2011).

### 4.1 Regional Variability of Soil Conditions

Soil is the main medium for the growth of halophytes, and its properties are decisive for the habitat (Tóth 2010a). Because of this close relationship, it is intuitional to compare the separation of halophyte species/associations into regions to that of the soil properties. Although some maps are available on the continental factors, they are not suitable for the regional/local factors providing uniform information for assessment. Depending on tradition and priorities, different (national/international) soil classification systems (of which there are many, including two “international”) distinguish salt‐affected soils at different hierarchical levels; therefore, not every soil map will express salinity at every level. Moreover, the use of a key to classes means that some properties are expressed at a higher level than others. In the case of salt‐affected soils, strong cracking will give priority over high salt content, and consequently, cracking saline soils will not be shown to be saline, but to be swelling‐shrinking soils at the first classification level of World Reference Base for Soil Resources maps (IUSS Working Group 2022). Using this international classification at 1:1500000 scale only Seewinkel and Alföld are distinguished among the studied subregions by the most widespread Paneuropean data source, the Soil Atlas of Europe (Jones et al. 2005). However, the high variability of halomorphic soil types in Alföld (Solonchak, Solonchak‐Solonetz, and Meadow Solonetz) explains the highest variability of vegetation/species composition as well (Szatmári et al. 2020). Salt‐affected soils in the North German Plain (mostly Thuringia) are in a great proportion anthropogenic due to the contamination of soda/potash industry (Schuster et al. 2010). These soils cannot be classified into the halomorphic kind of soils similarly as the histosols and gleysols in Kujawy (see Appendix S1) where for the Molli‐Sodic Solonchaks a new unit grouping industrial and urban soils (“Technosols”) was introduced due to the large amounts of fluid wastes of the soda processing (Hulisz et al. 2010). Saline soils of the Transylvanian Basin are more or less uniform, where the main role is played by the NaCl content and moisture depending on the spring activity in aRer certain season (Kis et al. 2012; Dítě et al. 2021; Dítě, Šuvada, and Dítě 2023). Weindorf et al. (2011) showed that 0.1% of total soil cover of the Transylvanian Basin is made up by the salt‐affected soil category named “Salsodisols”.

### 4.2 Analogies With Other Territories of Inland Saline Vegetation

The salt flora of central Europe carries less than half of the halophytes recorded in the two nearest important territories of saline habitats in Eurasia; however, these enumerations include the coastal areas as well. One significant province of saltmarshes and saline steppes is in the interior of the Iberian Peninsula in the La Mancha, Ebro, and Duero basins (Ladero et al. 1984; Fernández‐González et al. 2017; Loidi 2017; Penas et al. 2017). The newest inventory of strictly halophytic vegetation (Appendix II in Salazar‐Mendías and Lendínez 2021) reports 241 taxa (206 species and 35 subspecies) there. The inland saline habitats of our study area share 21 halophytes with the Iberian saline habitats. The number of endemic species is extremely high there (*Limonium* genus) compared to central Europe, where there are only three endemic halophytes occurring exclusively in the Pannonian Lowland. The phytocoenological classification of the Iberian saline vegetation encompasses 11 classes, 16 orders, 30 alliances, and 139 associations (Appendix I in Salazar‐Mendías and Lendínez 2021).

The second territory of halophytic vegetation is in the East European Plain, also marked by high coenotic diversity for its steppic climate and hyperspace of ecological conditions (Dziuba and Dubyna 2021). Around 250 halophytes are recorded in Ukraine, including the coastal part *(T. Dziuba pers. comm)*. The syntaxonomic structure includes 6 classes, 12 orders, 27 alliances, and 115 associations (Dubyna et al. 2019). Endemic and subendemic species are diagnostic for syntaxa of different ranks (most often the level of association). Six plant alliances are in common with the inland saline habitats of our study area (*Therosalicornion, Puccinellion limosae, Juncion gerardi, Beckmannion eruciformis, Festucion pseudovinae, Cypero‐Spergularion salinae*), and there are 83 halophytic taxa in common. That is much more than the shared species of Iberian salt flora. The higher similarity in floristic composition with Ukraine we explain by the strong relation of the central‐eastern European flora with the Black Sea coast which we confirmed in the case of the Pannonian Lowland (Figure 5). A higher floristic diversity of the Pannonian saline habitats against the lower diversity of boreal and oceanic salt regions of Europe was pointed out by Molnár et al. (2018) which complies with the generalized pattern of “southern richness versus northern purity” defined by Hewitt (1996).

## 5 Conclusions

During the biogeographical assessment of 13 important areas of inland halophytic vegetation in eastern central‐Europe, we recorded 107 native halophytic plant specialists, from which the subregions have in common only one obligate and five facultative halophytes. We found a reduced number of halophytes endemic to the study area (three) compared to older enumerations and revealed a high proportion of coastal species, which are known mostly from the Black Sea coast. The size of the subregion and its distance from the nearest seas did not affect the halophyte richness; the decisive factor in the halophytic flora is the variability of opportunities for its existence. The leading position in the number of halophytes and vegetation types has the subregion Alföld, endowing also the most types of halomorphic soils. The decisive factor for its high variability is also the specific climate, featuring high seasonality of temperature and precipitation, enhancing summer evaporation of the soil and causing various salinization levels. The principal role of bioclimate underlines the intrazonality of Pannonian salt steppes and salt marshes, and the azonality of inland saline habitats of the Transylvanian Basin and North German and Polish Plain. Land use, habitat continuity, and soil properties undoubtedly contribute to the variability of inland saline/alkaline habitats, but due to their high patchiness from site to site, our study did not evaluate these attributes.

Our results addressing floristical relations of inland halophytes to the seacoasts can be applied in the ongoing investigations on phylogeographic patterns of plants from coasts and the interior (Prinz et al. 2010), or can also be integrated into multidisciplinary approaches like waterbird ecology, as important vectors of natural species dispersal between continental and maritime areas are the avifauna via endozoochory (Lovas‐Kiss et al. 2019).

The results of this study may be utilized also as a groundwork for future local to macroregional assessments in light of rapid vegetation dynamics, species‐area shifts, or biotic invasions induced by the ongoing climatic zone shifts (Skalák et al. 2018).

## Author Contributions

**Zuzana Dítě:** conceptualization (equal), data curation (supporting), funding acquisition (equal), investigation (lead), project administration (equal), writing – original draft (lead), writing – review and editing (lead). **Róbert Šuvada:** data curation (equal), formal analysis (lead), methodology (equal), visualization (lead), writing – original draft (supporting), writing – review and editing (supporting). **Tibor Tóth:** formal analysis (supporting), supervision (equal), writing – original draft (equal), writing – review and editing (equal). **Daniel Dítě:** conceptualization (equal), data curation (lead), funding acquisition (equal), investigation (lead), methodology (equal), writing – original draft (equal), writing – review and editing (equal).

## Ethics Statement

The authors confirm that all the research meets the ethical guidelines, including adherence to the legal requirements of the study country.

## Consent

The authors confirm that the manuscript has been submitted solely to this journal and is not published, in press, or submitted elsewhere.

## Conflicts of Interest

The authors declare no conflicts of interest.

## Supporting information


**Appendix S1.** Geography, landscape features and research history of 13 subregions with significant occurrence of inland saline habitats in eastern central Europe.

**Appendix S2.** Descriptive statistics of 19 bioclimatic factors for all subregions.

**Appendix S3.** Barchart plots for six variables (PCspec, PCassoc, PCclim, PCdiss, Area, Number of Species) showing the transformation of ordination scores and original values into rank categories.

**Appendix S4.** Source tables for the analysis with eight sheets: #1 Source PCspec, #2 Source PCasoc, #3 Source PCdiss, #4 Ordination scores+rank level, #5 Source Fig4, #6 Source Fig5, #7 Mutual and unique species, #8 Mutual and unique vegetation.

## Data Availability

All relevant data is contained in the manuscript's Supporting Information.
