# Supplementary material for: Diversity Drivers of Inland Saline Vegetation—What Unites Them and Divides Them?
Source: Ecol Evol. 2025 May 14;15(5):e71249. doi: 10.1002/ece3.71249 (PMC12078054; doi:10.1002/ece3.71249)
Supplement: Supplementary file 1 — Appendix S1. Geography, landscape features and research history of 13 subregions with significant occurrence of inland saline habitats in eastern central Europe. [file ECE3-15-e71249-s001.pdf]

Supplementary material to the article

Title: **Diversity drivers of inland saline vegetation – what unites them and divides them?**

Authors: Zuzana Dítě*, Róbert Šuvada, Tibor Tóth & Daniel Dítě

Journal: Ecology and Evolution

Appendix S1.

Geography, landscape features and research history of 13 subregions with significant occurrence of inland saline habitats in eastern central Europe.

A. North German and Polish Plain
1. Thüringen
2. Sachsen-Anhalt
3. Kujawy

B. Pannonian Lowland
4. Jižní Morava
5. Seewinkel
6. Podunajská nížina
7. Dunántúl
8. Východoslovenská nížina
9. Alföld

C. Transylvanian Basin
10. Câmpia Transilvaniei
11. Harghita

Isolated subregions
12. Mostecká pánev
13. Spiš

The subregion subtitle is in the following format:
Subregion name/country code/other frequently name used in the literature/size/elevation range.

1. Thüringen/DE/Thuringia/ 106 km^2/ 200 – 240 m

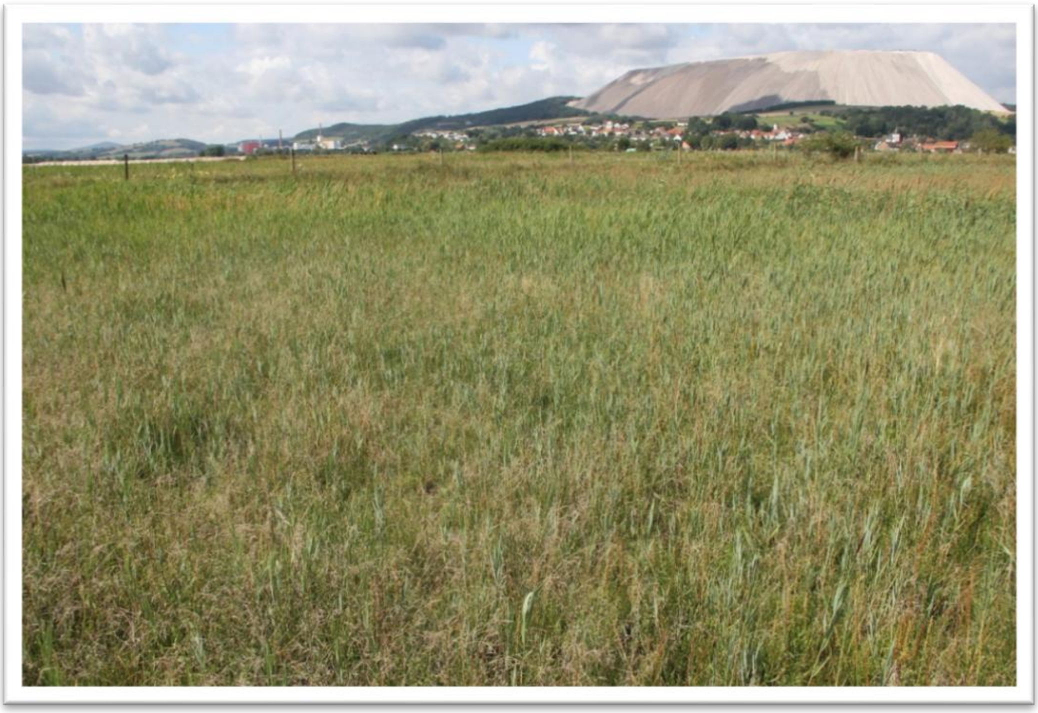

photo credit: authors

In this hilly, relatively humid territory, salt-affected soils are scattered in the middle section of the Werra River. The complete characteristics of salt-affected habitats are summarized in the publication by Westhus et al. (1997). Annual hypersaline succulent stands and moderately salt meadows are the most typical here, considerably affected by the soda industry and mining of potash and uranium (Krisch 1967). The large slag heaps in the landscape caused an infiltration of mineral salts into the groundwater which were transported by rivers to larger distances. The subregion was suffering from environmental damage caused by discharges of Europe's largest supplier of potash for fertilization in agriculture, which has been reduced to a large extent after 1990.

2. Sachsen-Anhalt/DE/Saxony-Anhalt/ 5357 km^2/ 40 – 130 m

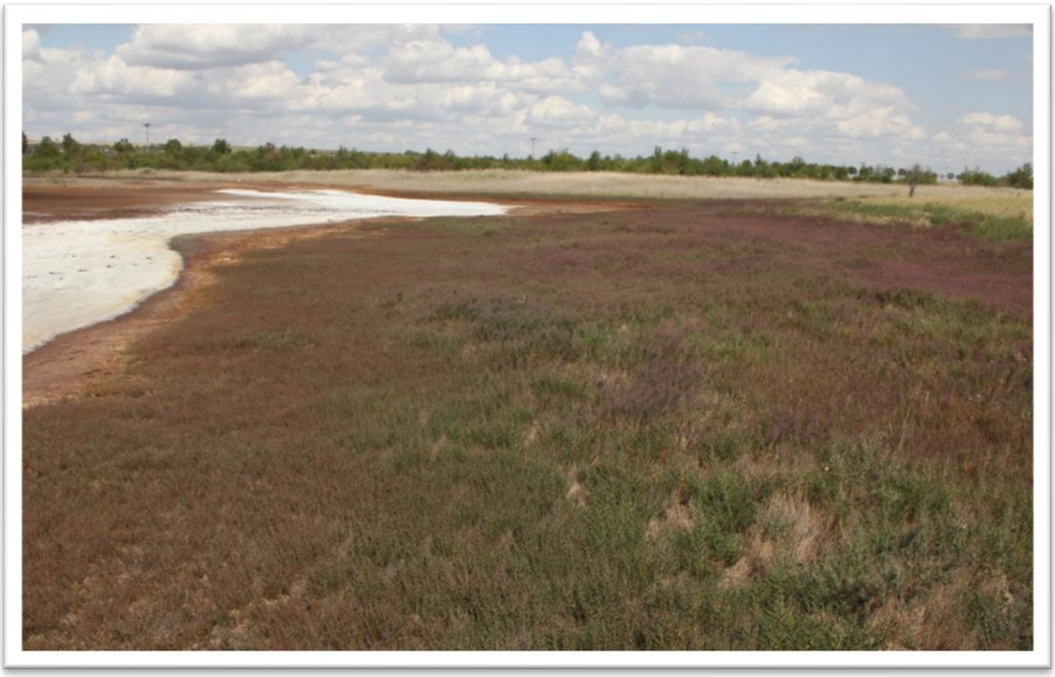

photo credit: authors

The gently undulating and largely treeless landscape comprises loose morainic material from the Saale glaciation period, the older bedrock is covered by wind-blown loess, noted for its fertile Chernozem soils. It is one of the driest regions in Germany, situated in the rain shadow of the Harz Mountains, enhancing the development of saline vegetation with a moderate steppe character. Saline soils are found in the lowlands of the Saale River and its tributaries, but secondary saline soils as a result of salt/potash mining and processing are also frequent. An important site of salt marshes near Artern we included in this subregion due to the similar topography and climate, however, it belongs administratively to the state of Thuringia (Barthel & Pusch 1993). Sachsen-Anhlat enjoys the longest history of floristic research on salt marshes, dating back to the 16th Century, the most comprehensive flora and vegetation study provided Altehage and Rossmann (1939). As one of the best surveyed territory of inland saline habitats, there were established many monitoring areas and active habitat conservation implications (e.g. Hartenauer et al. 2012).

3. Kujawy/PL/3204 km^2/ 75 – 90 (105) m

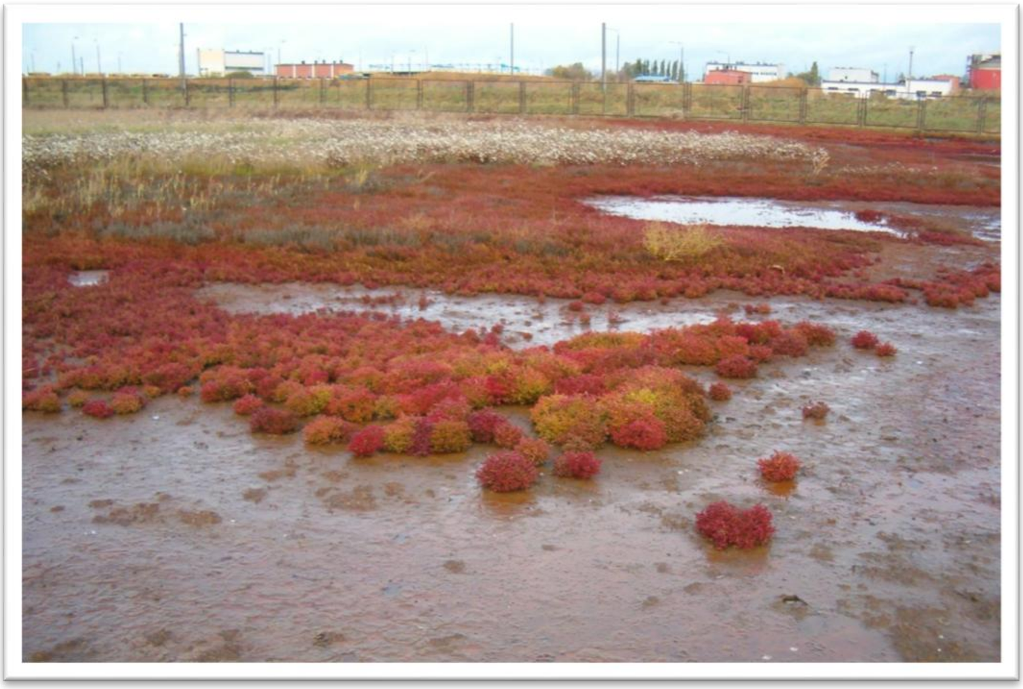

photo credit: Piotr Hulisz, published in doi.org/10.1080/00380768.2015.1028874

The subregion belongs to the Great Poland Lakeland (Kondracki 2000), located between the Vistula and the Noteć Górna rivers. The landscape is undulating with a few moraine hills and sandy gravel embankments. Under the glacial formations are rock-salt and potassium layers. With the lowering impact of the oceanic climate from the North Sea the continentality increases towards the east (Rivas-Martinez & Rivas-Saenz 2009), therefore it is one of the driest areas of central Europe. The rain shadow of the lake land elevations maintains natural soil salinity on a local scale (Piernik 2003), developed from alluvial sands and sandy loams, rich in $CaCO_3$ and strongly affected by gleyic properties, i.e. groundwater movements. Histosols and Gleysols are typical with neutral to weakly-alkaline reaction (Piernik & Hulisz 2011). Due to the occurrence of fossil salt strata, the exploitation of salt and brine deposits developed in Kujawy as well (e.g. in Inowrocław and Janikowo), which formed anthropogenic salt marshes and salt meadows near waste ponds of the factories and brine pipelines (Hulisz & Piernik 2013). Significant observations were carried out in the 19th and early 20th centuries by Prussian, German and Polish florists, which resulted in a comprehensive survey on the halophytic flora and vegetation by Wilkoń-Michalska (1963). Ecological conditions of temperate inland salt marshes show a wealth of recent knowledge, e.g. (Piernik 2005; 2012). Intensive human activity has rapidly changed the range of halophytes (Karasinska et al. 2021).

4. Jižní Morava/CZ/1596 km^2/ 170 – 210 (240) m

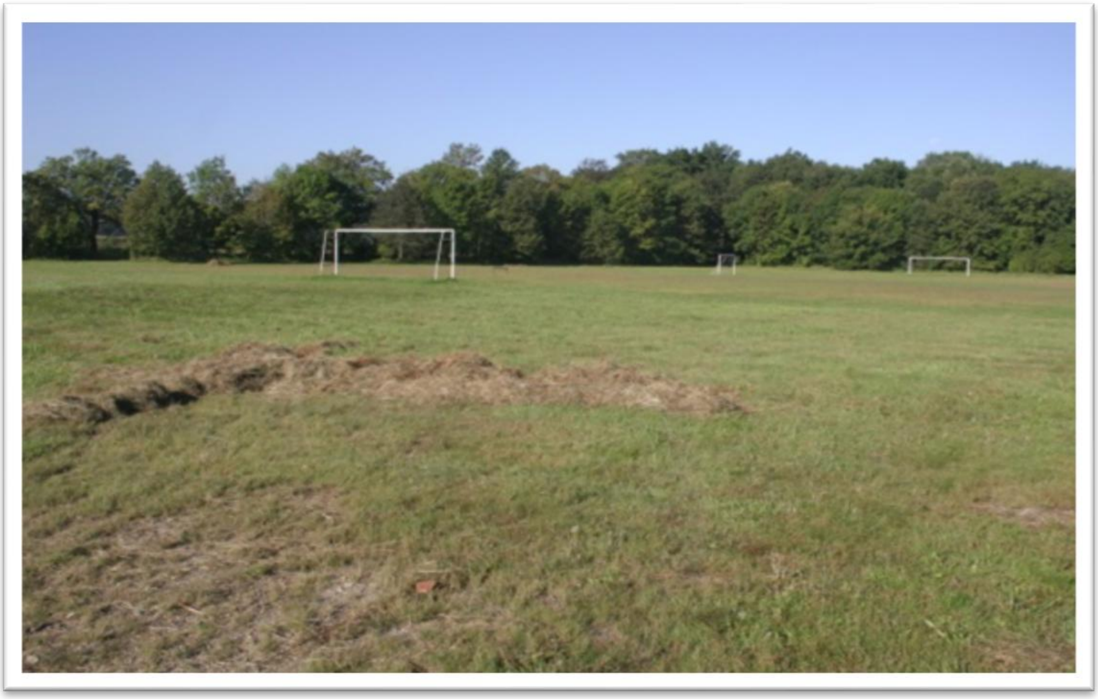

photo credit: authors

The NW enclave of the Pannonian Lowland lies in the Vienna Basin which is the warmest area of the Czech Republic. Small islets of saline soils occur in the Lower Morava Valley and the adjacent Svratka and Dyje river valleys. These soils developed from the young Tertiary clays rich in sodium, magnesium and calcium sulphates (Pelíšek 1948). The once-rich halophytic flora steadily declined from the early 19th century due to drainage and after the mid-20th century due to reclamation and abandonment (Šmarda 1953). The westernmost occurrence of *Achillea* salt steppes is reported here, however, they are regarded as degraded vegetation of former salt meadows (Danihelka et al. 2022). The succulent vegetation of *Salicornion prostratae* had disappeared in the study area by the 1970s (Grulich 1987). Restoration activities from the 1990s contributed to stabilizing the last sites of inland salt marshes and meadows.

5. Seewinkel, Fertőzug/AT,HU/ 308 km^2/ 110 – 120 m

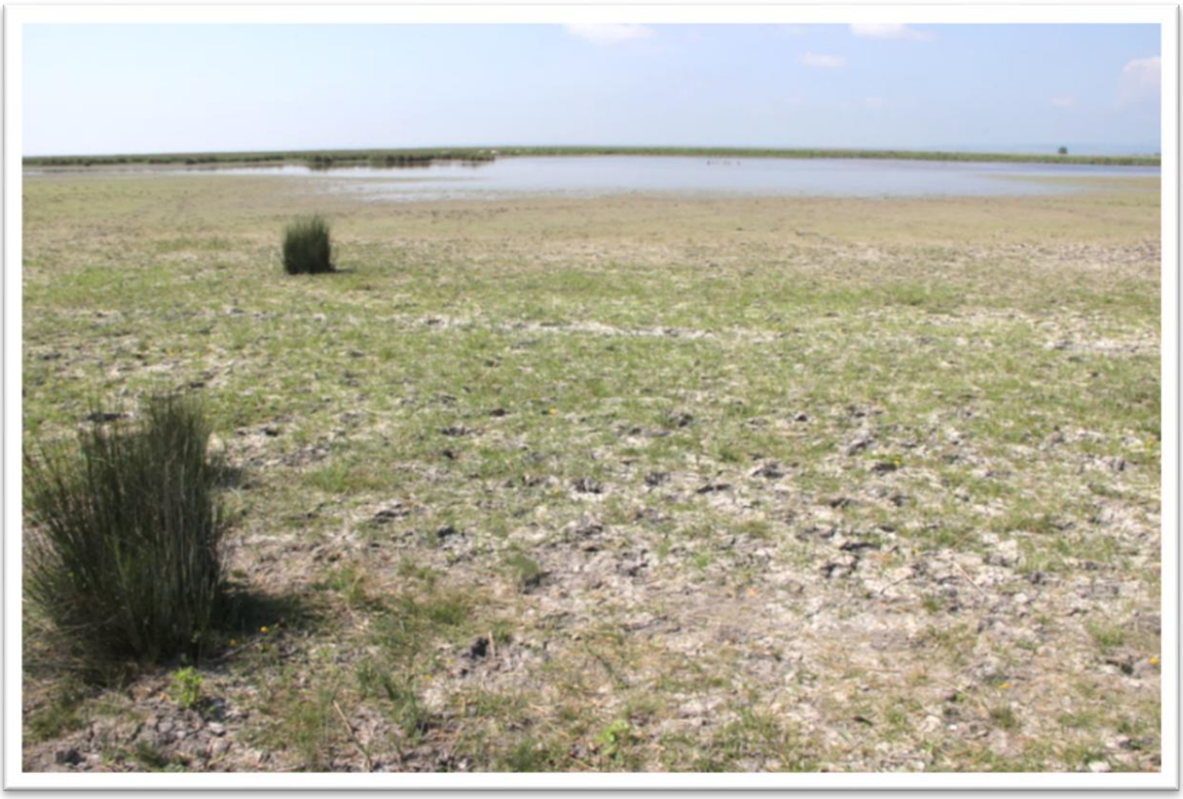

photo credit: authors

The subregion lies on also on the western periphery of the Pannonian Lowland, in the Little Danubian Plain/Kisalföld. The dominant landscape component is lake Fertő (Neusiedler See), known as the westernmost Eurasian steppe lake without runoff (Kárpáty & Fally 2012). The eastern side of the lake is a part of the alluvial fan of the ancient Danube covered with saline clay. The bedrock is made of Paleozoic crystalline slates which were later inundated by the Pannonian Sea, and the basin was filled up by silt of the inflowing watercourses. Main soil forming processes were silt deposition and salt accumulation, peat layers have also emerged leaving fens and swamp meadows to our times, mainly on the Hungarian side. Solonchak soils are typical, with predominating $NaHCO_3$ and Na_2SO_4 salts, locally NaCl. Saline habitats are more abundant on the Austrian side, forming several mosaics of astatic soda pans (Boros et al. 2013) and salt marshes, while salt steppes are sporadic (Wendelberger 1943). The drainage system built between 1892 and 1910 around the lake resulted in water decrease and reed expansion. After the establishment of the Fertő-Hanság and Seewinkel National Parks in the 1990s, artificial inundation partially restored the habitats (Király & Takács 2020), now it is protected by the highest national and international nature conservation standards (Albert et al. 2020).

6. Podunajská nížina/SK/2025 km^2/100–140 m

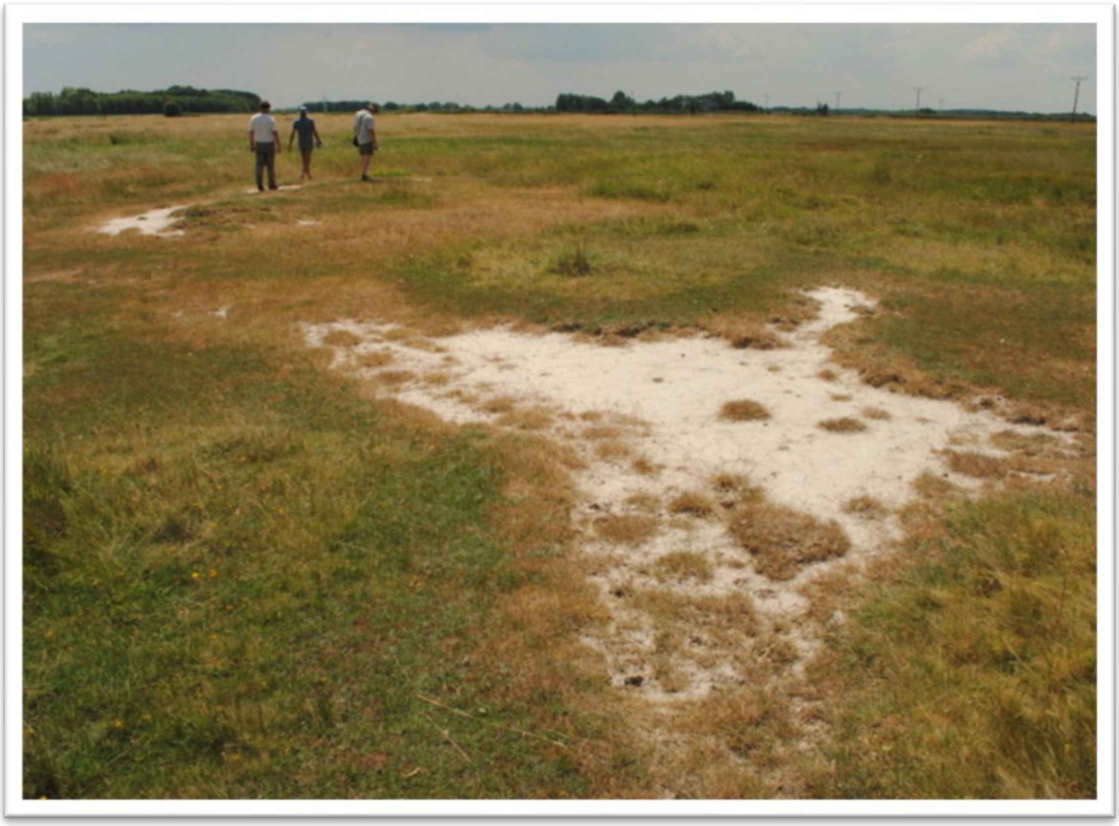

photo credit: authors

It is also part of the Little Danubian Plain which is divided from the Hungarian side (Kisalföld) by the Danube River. Podunajská nížina lies on its northern half, in SW Slovakia. The flat area is continuously sinking from the Miocene which was covered by the Pannonian Sea. Later, the Danube and its tributaries left rich sediments (gravel, loam, clay and loess), and Tertiary peat can also be found in the fillings of oxbows (Fordinál et al. 2022). It has one of the largest alluvial fans in Europe with fertile, intensively cultivated soils. The rivers are of a middle section character with rich meandering and, thus have been completely regulated. Podunajská nížina forms the northern border of the Pannonic salt steppes and marshes, which are concentrated in several islets on the eastern part of the plain (Dítě et al. 2014) on mostly Solonetz soils with high sodium content and pH (Kyntera 1937), on the western part there were vast fens (Bosáčková 1972). Rich flora confined to alkaline soils was observed by the 1960s (Krist 1940, Vicherek 1973), recently it has dramatically decreased (Sádovský et al. 2004). Beyond the loss in quantity, the vegetation suffers from degradation induced by the water table drop and leaching of soils via land reclamations, and abandonment Dítě et al. 2015). Conservation activities (re-introduction of grazing and local water retention) in the last 10 years have partially restored the bad habitats conditions.

7. Dunántúl/HU/1102 km^2/95 – 180 m

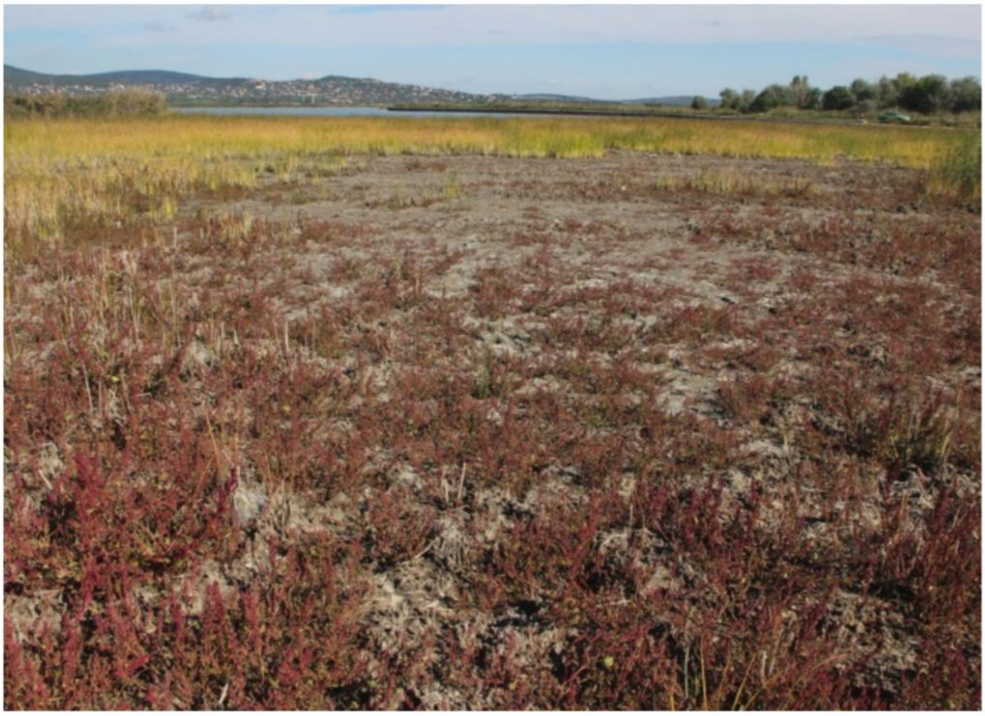

photo credit: authors

The division of the Pannonian Lowland in case of its peripheral subregions is more obvious due to sharper geomorphological boundaries, whereas the inner parts (e.g. Dunántúl and Alföld in Hungary) have smooth transitions and intermediate zones. West of the Danube River is the Transdanubian part of the Pannonian Lowland, called Dunántúl. Although its easternmost part, Mezőföld, phytogeografically belongs to Alföld (Boros 1959, Király et al. 2008) in this study we attach to Dunántúl, based on the geomorphological division of Pécsi (1970) where the borderline is the Danube basin dividing the Dunántúl from the lower lying Alföld by a 30-50 m high valley flank. The landscape is a grassy, open-woody mosaic with permanent wetlands. There are three core areas of saline habitats, all strongly connected to water bodies: the alluvium of the highly fluctuating Sárvíz stream (recently regulated), the shores of the shallow brackish lake Velence (26 km^2) - both match with the above-mentioned Mezőföld intermediate zone, and the southern shores of the Balaton lake (600 km^2) which is a graben depression formed by repeated subsidence. The latter features an interesting vegetation history: between the southern lakeshore dunes Calcic Histosols and Calcic Gleysols developed forming relic peatlands at the beginning of the Holocene (Pécsi 1970). Today the growing human pressure on the Balaton Lake (tourism and land reclamations) significantly decreased the permanent water cover of the fens, and in the last 50 years secondary salinization has taken place (Bauer 2022). Similar processes were going on in Mezőföld also due to the vast hydromeliorations starting from the early 19th century (Boros 1937).

8. Východoslovenská nížina/SK/1313 km^2/100 – 120 m

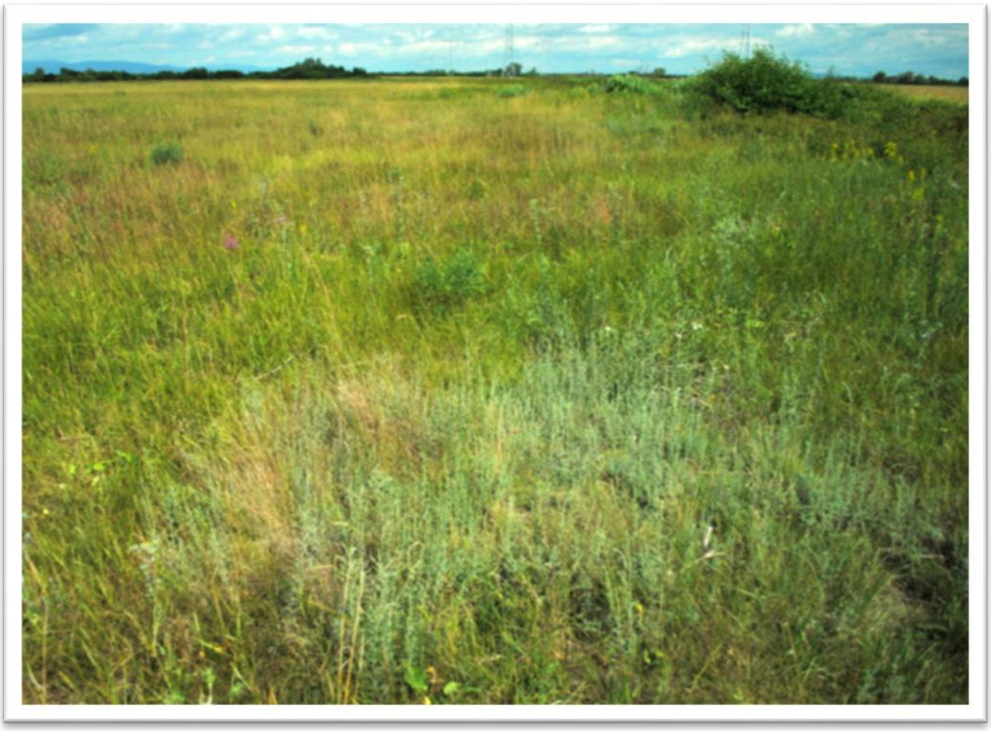

photo credit: authors

The NE appendix of the Pannonian Lowland is a tectonically developed young plain filled with river sediments in the Pleistocene and formed by eolian activity in the Holocene by depositing loess clays and fine sands. Many old riverbeds are in the subregion, around which the upper Tisza and its tributaries (Bodrog, Latorica, Laborec, Uh, Ondava and Topľa) have left marshy areas with poor runoff. At higher river levels, depressions are often flooded, the small slope of the plain and the low water permeability of the soils result in waterlogging and development of hydromorphic soils (Gleysols) where significant wetlands and fens occurred, strongly suffering from melioration from the 1960s. Locally, where negative water balance is in the soil during the summer droughts and the groundwater is even richer in minerals, islets of Solonetz soils have developed. The salts are accumulated at a depth of 1 m below the surface (Vilček 2004). The halophytic vegetation (mostly moderately saline steppes and meadows) is scattered among the vast arable fields and impoverished secondary wetlands.

9. Alföld/Great Hungarian Plain/HU, SRB, RO/43502 km^2/80 – 120 m

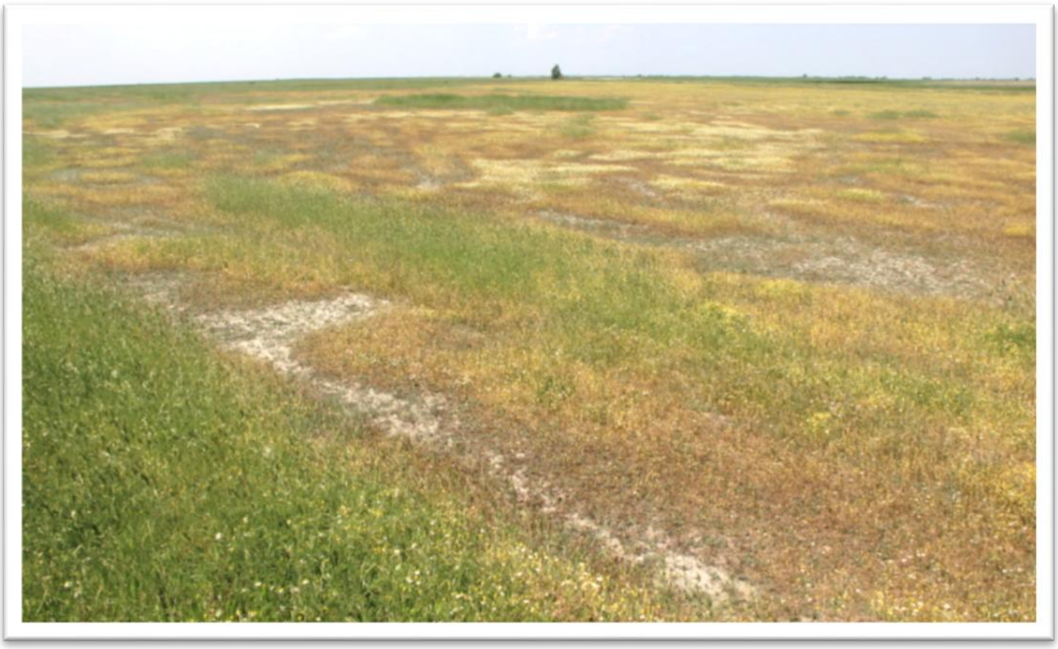

photo credit: authors

The largest, central unit of the Pannonian Lowland is Alföld, spanning from the Danube Basin to the east through the Tisza Basin to the foothills of the Eastern and South Carpathians. Prior to the flood control works, these rivers roamed over a vast area stepping out of its beds (Pécsi 1970, Mezősi 2011). The retained water in the basin-shaped, undrained depressions evaporated in the hot dry summer season, contributed to the development of shallow astatic soda pans (Boros et al. 2013). Paleo-ecological studies revealed that minimal forest cover of the marshland-grassland complex existed since the late Pleistocene (Barczi et al. 2006), and it has never been completely wooded in the Holocene. By the Early Middle Ages the forest cover had been reduced to less than 50%, leaving vast pasturelands (Molnár et al. 2018). The floodplains were cut by arable lands only on higher loess plateaus, but after water regulation the majority of low productive grasslands were also ploughed up (Biró et al. 2013). Nevertheless, saline and alkali habitats are still wide-spread in both river basins: in the depressions of the windblown sandy ridges of the Duna-Tisza Interfluve, and along and east of the Tisza River which are connected to the Tisza tributaries (Berettyó, Körös, Maros and Timiş). The halophytic vegetation was largely studied at the first half of the 20th century in Hungary (e.g. Rapaics 1927, Magyar 1928), as well as in Voyvodina, Serbia (Slavnić 1948), and also in Romania (e.g. Ţopa 1939, Pop 1968).

10. Câmpia Transilvaniei/RO/557 km^2/270 – 380 m

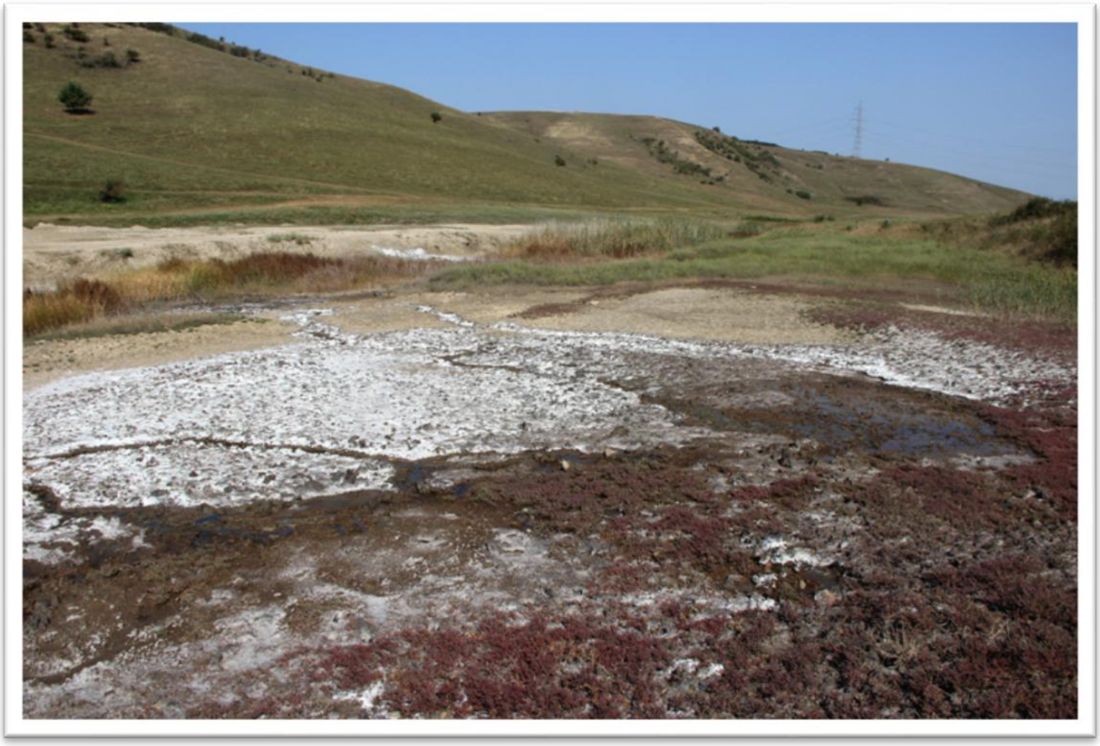

photo credit: authors

The hilly landscape is formed by a network of valleys, ravines and canyons and is almost completely deforested which is carried in its Romanian and Hungarian names (*Câmpia Transilvaniei*, *Erdélyi Mezőség*). Among the semi-natural non-forest habitats, the extremely species-rich dry grasslands maintained by traditional mowing are well studied (Ruprecht 2005) compared to the salt-affected vegetation. Floristic and vegetation surveys have been conducted scatterly in the past (Todor 1947, 1948; Pop 2002), the latest salt vegetation survey provided Dítě et al. (2021). Natural salt domes and diapirs developed from the Miocene salt deposits (Krézsek and Bally 2006). The most frequent occurrence of vegetation confined to salt domes is in Cluj County in the catchment area of the Someşul Mic and Mureş rivers. Salt mining dating back to the Middle Ages in Sic and Turda has contributed to further development of salt-affected areas (Jakab et al. 2019). Near the old mines and salt ponds, the underground water can easily come into contact with soluble salt deposits and emerge as salt springs. The surface erosion created wide flat meadows accompanied by salt springs and sapropelic mud plateaus, both inhabited by highly specialized vegetation of succulent hypersaline stands and saline meadows (Podar et al. 2019). Salt domes and mines are distributed elsewhere within the Transilvanian Basin, e.g. Ocna Sibiului or ocna Mures, which were not included in this subregion due to their distant geographical locations.

11. Harghita/RO/1599 km^2/450 – 650 (760) m

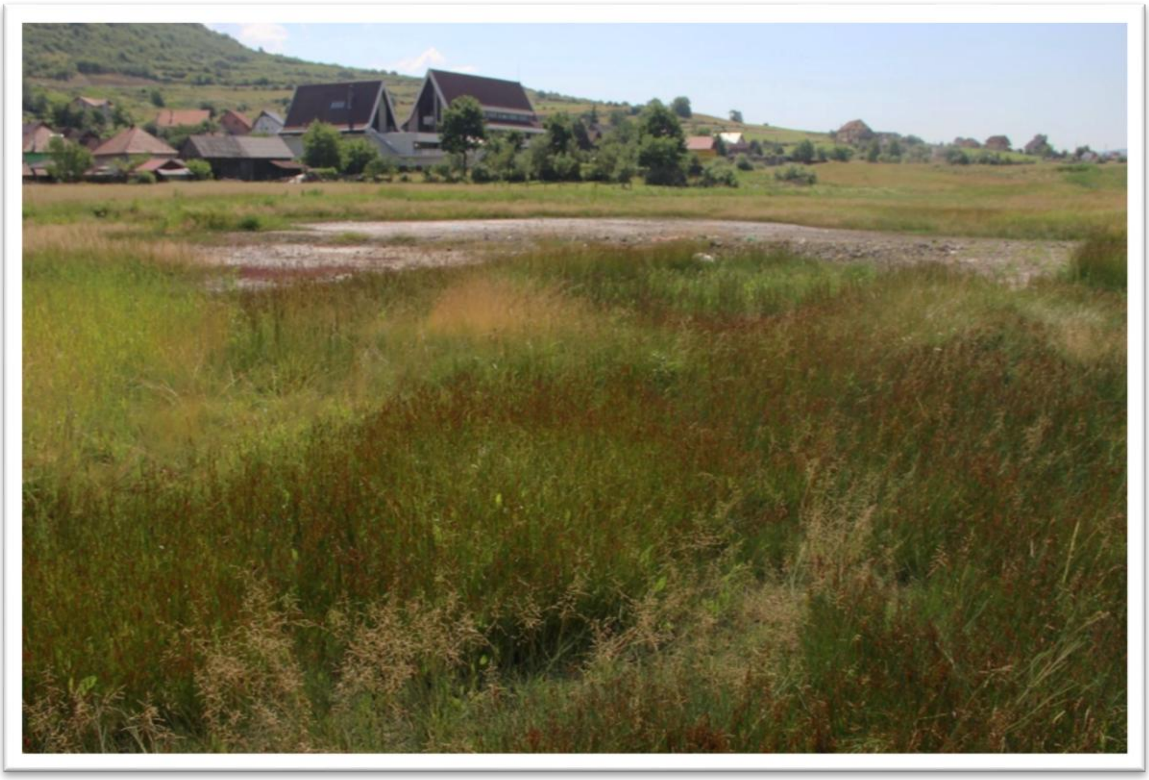

photo credit: authors

The climate in the Eastern Carpathian subregion (Harghita and partially Brasov counties) is mountainous, with long-lasting cold winters and relatively warm summers (Kun et al. 2004). The contact area between the Transylvanian basin and the Inner Eastern Carpathians (the crystalline Harghita Mts.) is one of the most important areas of sparkling mineral water resources in Europe (Kis et al. 2012) with rich occurrence of $NaHCO_3$ and CO_2-rich cold springs, bore wells and dry mofettes (Vaselli et al. 2002). In the Tertiary period, the active compression tectonics caused diapiric intrusions of halite deposits and associated saline, CO_2-rich waters along active faults (Szakács and Krézsek 2006). Such diapirs are recently mined for halite, the most famous is in Praid. Water circulation in the salt massifs leads to intense water mineralization after the NaCl leaching process. Vegetation affected by salt outcrops and salt springs was described very scarcely (e.g. in Kovács 2004), their revision including recent field data was provided only recenlty (Dítě et al. 2023). Compared to the saline areas in Câmpia Transilvaniei, these are smaller in size, and only three euhalophytic plant commnunities make up the vegetation zonation.

12. Mostecká pánev/CZ/1295 km^2/180 – 300 m

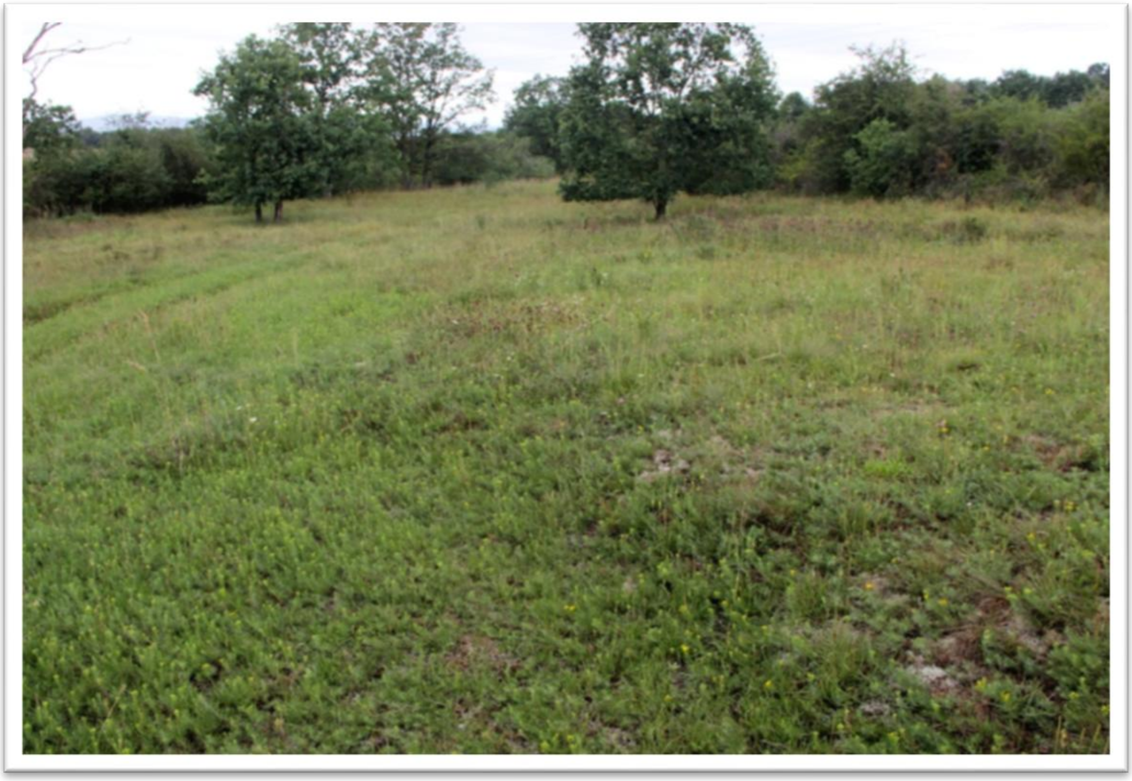

photo credit: authors

The region is a part of the Podkrušnohorská oblast in NW Bohemia in a zone of tectonic depressions and volcanic hills, created during tectonic movements in the Tertiary period. In the basin there are deposits of lignite, mined from the 17th century. Outcrops of CO_2 along the tectonic faults created mineral springs where subhalophytic wetland vegetation has developed, the main salt is $MgSO_4$ (Toman 1976). Except other geochemical and topographical peculiarities, like the flat terrain with bad water runoff, specific climatic conditions also took part in shaping the subhalophytic vegetation. The region is in a rain shadow which is indicated mainly by long dry periods when evaporation resulted in salt accumulation in the root zone, salt crystals were often observed on the top soil (Sládek 1988). Wendelberger (1950) adressed a question whether this salt district it is more related to Moravia, based on the occurrence of several species of the Pannonian Lowland or it is more related to the geographically closer saline vegetation of inland Germany. Recently these habitats have not been preserved, several halophytic species vanished (Novák 2000), and the remnants are formed by stands of *Plantago maritima*.

13. Spiš/SK/ 25 km^2/ 435 – 605 m

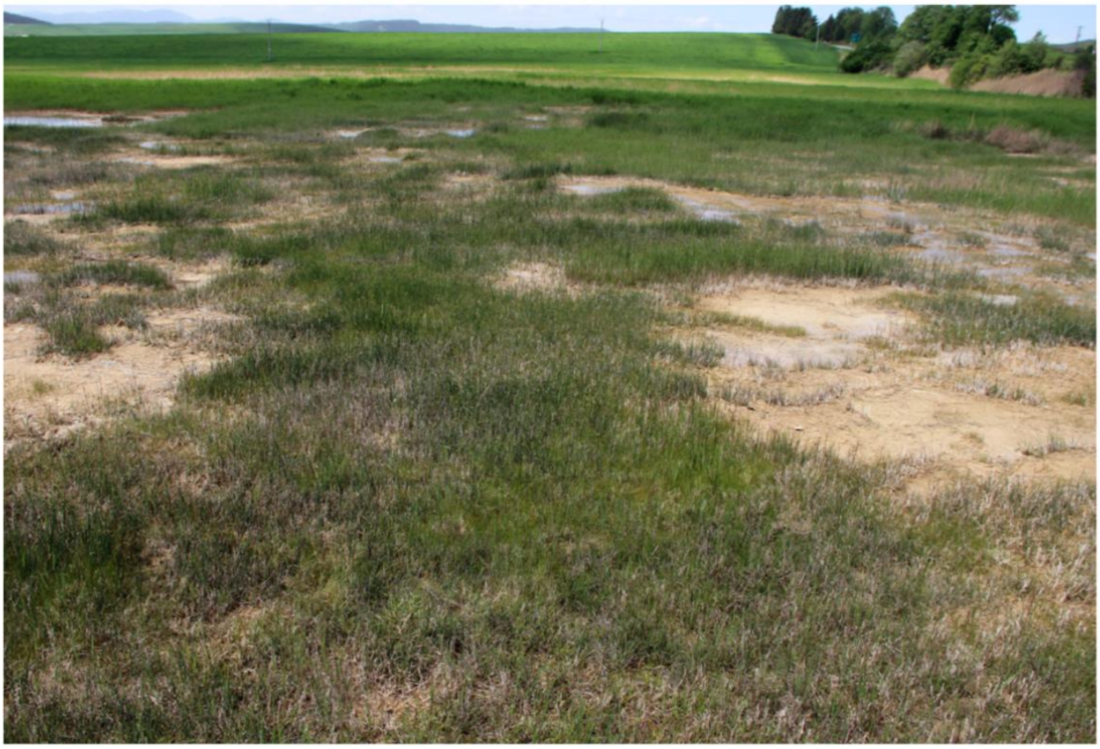

photo credit: authors

Situated in the Popradská and Hornádska kotlina basins in the rain shadow of the Western Carpathians, this subregion has remarkable geological features (Ložek 1964), however, it has been overlooked within the scope of recent halophytic vegetation surveys of Europe (e.g. Eliáš et al. 2013). Along tectonic faults, mineral waters reaching the surface have gradually deposited extensive and thick (more than 50 m) travertine cones from the end of the Pliocene, and the accumulation lasts until today (Tometz 1997). A highly specialized habitat is bound to strongly mineralized cold water with a high saturation of dissolved minerals like sulfates, CO_2, and H_2S, as well as compact travertine (Michaeli 1998). The vegetation inhabiting the travertine formations consists of a mixture of (sub)halophytic and calcareous fen species, which belong to the association *Glauco-Trichophoretum pumili* (Šmarda 1961) showing high electric conductivity of the soil (Dítě et al. 2004).

References

Albert R., Werner V. & Popp M. with contributions by Fischer M.A. & Niklfeld H., 2020. Botanical treasures in front of us – Plants of saline habitats in the Lake Neusiedl Region, Burgenland. Acta ZooBot Austria 157, 2020, 115–143

Altehage C. & Roßmann B. 1939. Vegetationskundliche Untersuchungen der Halophytenflora binnenländischer Salzstellen im Trockengebiet Mitteldeutschlands. – Beihefte zum botanischen Centralblatt 60/B: 135–180.

Barczi A., Tóth M.T., Csanádi A., Sümegi P. & Czinkota I. 2006. Reconstruction of the paleo-environment and soil evolution of the Csípo-halom kurgan, Hungary. } Quarternary International 156–157: 49–59.

Barthel K. J. & Pusch J. 1993. Zum Vorkommen der Kleinblütigen Schwarzwurzel (Scorzonera parviflora Jacq.) im Esperstedter Ried (Landkreis Arten). Landschaftspflege u Naturschutz Thüringen 30: 101–102.

Bauer N. 2022. Kiegészítések Külső-Somogy és a Balaton déli partmelléke flórájához és növényföldrajzához. Botanikai Közlemönyek 109(2): 109–163.

Biró M., Czúcz B., Horváth F., Révész A., Csatári B., & Molnár Z. 2013. Drivers of grassland loss in Hungary during the post-socialist transformation (1987–1999). Landscape ecology 28: 789–803.

Boros Á. 1937. Fejér-vármegye növénytakarója Magyar Városok és Vármegyék Monográfiája, Budapest.

Boros Á. 1959. A Mezőföld növényföldrajza. In: Ádám L. et al. (Eds.), A Mezőföld természeti földrajza, Akadémiai Kiadó, Budapest. pp. 363–383.

Boros E., Ecsedi Z. & Oláh J. 2013. *Ecology and management of soda pans in the Carpathian Basin*. Hortobágy Environmental Association, Balmazújváros, HU.

Bosáčková E. 1972. Súčasný stav a ochrana slatinnej vegetácie Žitného ostrova. SÚPSOP v Prírode, Bratislava. 82 pp.

Danihelka J., Chytrý K., Harásek M., Hubatka P., Klinkovská K., Kratoš F., Kučerová A., Slachová K., Szokala D., Prokešová H., Šmerdová E., Večeřa M. & Chytrý M. 2022. Halophytic flora and vegetation in southern Moravia and northern Lower Austria: past and present. Preslia 94: 13 –110

Dítě D., Eliáš. P. jun., Sádovský M. 2004. Recentný výskyt halofytov v Liptovskej a Spišských kotlinách (severné Slovensko). Bulletin Slovenskej Botanickej Spoločnosti Suppl. 10:117–121.

Dítě D., Melečková Z., Eliáš, P. jun. 2014. *Festuco-Puccinellietea*. In: Hegedüšová Vantarová K., Škodová I., (Eds.), Plant communities of Slovakia 5. Grassland vegetation. Bratislava (SK): Veda; p. 483–510.

Dítě D., Melečková Z., Eliáš jun. P. & Senko D. 2015. Nezvratný zánik biotopov v krajine na prípade slanísk v k. ú. Palárikovo (JZ Slovensko). Phytopedon 14/1: 55–32.

Dítě D., Šuvada R. & Dítě Z. 2021. Habitat shaped by ancient salt: inland halophytic plant communities of the Transylvanian Basin (Romania). Folia Geobotanica 56: 109–123.

Dítě Z., Šuvada R. & Dítě D. 2023. Isolated occurrence of halophytic vegetation on mineral springs in the Eastern and Western Carpathians. Folia Geobotanica 57: 231–246.

Eliáš P. jun., Sopotlieva D., Dítě D., Hájková P., Apostolova I., Senko D., Melečková Z. & Hájek M. 2013. Vegetation diversity of salt-rich grasslands in the south-east Europe. Applied Vegetation Sciences 16: 521–537.

Fordinál K., Maglay K., Moravcová M., Vitovič L., Nagy A., Šimon L. & Šefčík P. 2022. Geologická mapa Podunajskej nížiny – juhovýchodná časť 1:50 000 MžP, ŠGÚDŠ

Grulich V. 1987. Slanomilné rostliny na jižní Moravě (katalog historických lokalit). Český svaz ochránců přírody, Břeclav.

Hartenauer K., Balaske P., Jentzsch M., John H., Kainz W., Süssmuth T., Stark A., Spitzenberg D., Stottmeister L. & Trost M. (2012) Beschreibung der Binnenlandsalzstellen in den FFH-Gebieten. In: Binnenlandsalzstellen im Schutzgebietssystem Natura 2000 des Landes Sachsen-Anhalt. − Naturschutz Land Sachsen-Anhalt (Halle) 49 (SH): 66−155.

Hulisz P. & Piernik A. 2013. Soils affected by soda industry in Inowrocław. In Technogenic Soils of Poland, Eds. Charzyński P, Hulisz P, Bednarek R, pp. 125–140. Polish Society of Soil Science, Toruń.

Jakab G., Silye L., Sümegi P., Tóth A., Sümegi B., Pál I. & Benkő E. 2019. Relict Anthropogenic Ecosystem from the Middle Ages: History of a Salt Marsh from Transylvania (Sic, N Romania), Environmental Archaeology 25: 96–113.

Karasińska W, Nienartowicz A, Kunz M, Kamiński D, Piernik A. 2021. Resources and dynamics of halophytes in agricultural and industrial landscapes of the western part of Kujawy, Central Poland. Ecological Questions 32/4: 1–26.

Kárpáty L. & Fally J. 2012. Fertő-Hanság - Neusiedler See-Seewinkel Nemzeti Park. Monografikus tanulmányok a Fertő és a Hanság vidékéről. Fertő-Hanság Nemzeti Park Igazgatóság – Szaktudás Kiadó Ház, Budapest.

Király G. & Takács G. 2020. A magyar Fertő edényes flórája. A Fertő–Hanság Nemzeti Park Igazgatóság tanulmánykötetei 3. Fertő–Hanság Nemzeti Park Igazgatóság Sarród.

Király G., Molnár Zs., Bölöni J. & Vojtkó A. (eds.) 2008. Magyarország földrajzi kistájainak növényzete. MTA Ökológiai és Botanikai Kutatóintézete, Vácrátót

Kis B.M., Czellecz C., Baciu C. & Kékedy-Nagy L. 2012. Hydrogeochemical features of some mineral waters at the contact between Harghita Mts. (Eastern Carpathians) and the Transylvanian Basin. Procedia Environmental Sciences 14:195–206.

Kovács J. A. 2004. Syntaxonomical checklist of the plant communities of Szeklerland (Eastern Transylvania). Kanitzia 12: 75–149.

Krisch H. 1967. Die Grünland- und Salzpflanzengesellschaften der Werraaue bei Bad Salzungen Teil II: Die salzbeeinflußten Pflanzengesellschaften. Hercynia 5: 49–95.

Krist V. 1940. Halofytní vegetace jihozápadního Slovenska a severní části Malé uherské nížiny Práce Moravské přírodovědecké společnosti 12: 1–100.

Kyntera V. 1937. Soľné pôdy, ich vlastnosti a zlepšovanie so zvláštnym zreteľom na sôlné pôdy na Slovensku. Sborník výzkumných ústavov zemědělských v býv. ČSR, Praha, 157: 343 pp.

Kun A., Ruprecht E. & Szabó A. 2004. Az Erdélyi-medence bioklimatológiai jellemzése. *Múzeumi Füzetek* 13:63–81.

Ložek V. 1964. Genéza a vek spišských travertínov. Sbornik Východoslov Múz Košiciach, Séria 5A: 7–33.

Magyar P. 1928. Adatok a Hortobágy növényszociológiai és geobotanikai viszonyaihoz. Erdészeti. Kisérletek 30: 26–63.

Mezősi G. 2011. Az Alföld természeti képének kialakulása. In: Rakonczai J. (ed.) A környezeti változások és az Alföld. Nagyalföldi alapítvány kötetei 7. Békéscsaba, pp. 15–24.

Michaeli E. 1998. Národná prírodná rezervácia Sivá Brada a okolie. Folia Geographica 1:271–301.

Molnár Zs., Király G. & Fekete G (eds.) 2018. National Atlas of Hungary – Natural environment. In: Kocsis K. (Editor-in-Chief): National Atlas of Hungary. Budapest, MTA CSFK Geographical Institute. pp. 94–103.

Novák J. 2000. Které podmínky prostředí významně ovlivňují česká slaniska? Severočes. Přír. 32: 37–43.

Pécsi M. 1970. Geomorphological regions of Hungary. Akadémiai Kiadó, Budapest.

Pelíšek J. 1948. Solné půdy jižní Moravy. Sborník Vysoké školy zemedělské v Brne, Brno, 1–23

Piernik A. 2003. Inland halophilous vegetation as indicator of soil salinity. Basic Applied Ecology 4: 525–536

Piernik A 2005. Vegetation-environment relations on inland saline habitats in Central Poland. Phytocoenologia 35: 19–38.

Piernik A., Hulisz P. 2011. Soil-plant relations in inland natural and anthropogenic saline habitats. The European Journal of Plant Sciences and Biotechnology 5: 37–43.

Piernik A. 2012. Ecological pattern of inland salt marsh vegetation in Central Europe. – Nicolaus Copernicus University Press, Toruń.

Piernik A., Hulisz P. & Rokicka A. 2015. Micropattern of halophytic vegetation on technogenic soils affected by the soda industry. Soil Science and Plant Nutrition, 61(sup1): 98–112.

Podar D., Macalik K., Réti K., Martonos I., Carpa R., Török E., Csiszár J., & Szekely Gy. 2019. Morphological, physiological and biochemical aspects of salt tolerance of halophyte *Petrosimonia triandra* grown in natural habitat. Physiology and Molecular Biology of Plants 25: 1335–1347

Pop I. 1968. Flora si vegetatia Cimpiei Crisurilor Acad. Rebubl. Soc. Romania, Bukuresti.

Pop I. 2002. Vegetatia solurior saraturoase den Romania. Contributii Botanice 35: 287 – 332.

Pop I., Cristea V. & Hodişan I. 2002. Vegetaţia judeţului Cluj. (Studiu fitocenologic, ecologic, bioeconomic şi eco-protectiv). Contributii Botanice 35: 5–254.

Rapaics R. 1927. A Középtiszavidéki szikes talajok növényszövetkezetei. Debreceni Szemle: 194–210.

Rivas-Martinez S. & Rivas-Saenz S. 2009. Worldwide bioclimatic classification system 1996–2009. Phytosociological Research Center, Madrid, ES. Available at http://www. globalbioclimatics.org, accessed on December 15, 2024.

Ruprecht E. 2005. Secondary succession in old-fields in the Transylvanian Lowland. Preslia 77: 145–157

Sádovský M., Eliáš P. jun. & Dítě D. 2004. Historické a súčasné rozšírenie slaniskových spoločenstiev na juhozápadnom Slovensku. Bulletin Slovenskej Botanickej Spoločnosti, Supl. 10: 127–129.

Sládek J. 1988. Současný stav slanobytné vegetace na Mostecku. Sborník Okresního muzea v Mostě. Rada přírodovedná 10: 43–59.

Slavnić Ž. 1948. Slatinska vegetacija Vojvodine. Arhiv za poljoprivredne nauke i tehniku (Beograd) 3(4): 76–142.

Szakács A. & Krézsek Cs. 2006. Volcano-basement interaction in the Eastern Carpathians: explaining unusual tectonic features in the Eastern Transylvanian Basin, Romania. Journal of Volcanology and Geothermal Research 158: 6–20

Šmarda J. 1953. Halofytní květena jižní Moravy. Práce Moravskoslezské akademie věd přírodních 25: 121–168.

Šmarda J. 1961. Vegetační poměry Spišské kotliny. SAV, Bratislava. 268 pp.

Todor I. 1947. Flora şi vegetaţia de la Băile Sărate-Turda I. Buletinul Grădinii Botanice Cluj 27: 1–64.

Todor I. 1948. Flora şi vegetaţia de la Băile Sărate-Turda II. Buletinul Grădinii Botanice Cluj 28: 21–174.

Toman M. 1976. Halofilní květena severozápadních Čech. Preslia 48: 60–75.

Tometz L. 1997. Inžinierskogeologické pomery travertínových kôp a ich širšieho okolia pri Spišskom Podhradí. Acta Montanistica Slovaca 2: 167–176.

Ţopa E. 1939. Vegetaţia halofitelor din Nordul României în legătură cu cea din restul țării. PhD. Thesis. Bul. Fac. Sţi. Cernăuţi, 13: 1–80.

Török P., Valkó O., Deák B., Kelemen A., Tóth E. & Tóthmérész B. 2016. Managing for species composition or diversity? Pastoral and free grazing systems in alkali steppes. Agriculture, Ecosystems & Environment 234: 23–30.

Vaselli O., Minissale A., Tassi F., Magro G., Seghedi I., Ioane D. & Szakács A. 2002. A geochemical traverse across the Eastern Carpathians (Romania): constraints on the origin and evolution on the mineral water and gas discharges. Chemical Geology 182: 637–654.

Vicherek J. 1973. Die Pflanzengesellschaften der Halophyten- und Subhalophytenvegetation der Tschechoslowakei. Academia, Praha.

Vilček J. 2004. Geografia poľnohospodárskych pôd Východoslovenskej nížiny. Prírodné vedy - Folia Geographica 7 roč. XLII, Acta facultatis studiorum humanitatis et naturae Universitatis Prešoviensis, Prešov, pp. 220–246

Wendelberger G. 1943. Die Salzpflanzengesellschaften des Neusiedler Sees. Wiener Botanische Zeitung, Wien, 3: 124–144.

Wendelberger G. 1950. Zur Soziologie der kontinentales Halophytenvegetation Mitteleuropas. Österr.Akademie der Wissenschaften, Mathematisch-Naturwissenschaftliche Klasse, Wien, 108: 1–28 + Tab.

Westhus W., Fritzlar F., Pusch J., van Elsen T. & Andres C. 1997. Binnensalzstellen in Thüringen – Situation, Gefährdung und Schutz. Naturschutzreport, Jena, 12: 1–193.
