# Supplementary material for: Diversity Drivers of Inland Saline Vegetation—What Unites Them and Divides Them?
Source: Ecol Evol. 2025 May 14;15(5):e71249. doi: 10.1002/ece3.71249 (PMC12078054; doi:10.1002/ece3.71249)
Supplement: Supplementary file 2 — Appendix S2. Descriptive statistics of 19 bioclimatic factors for all subregions. [file ECE3-15-e71249-s002.pdf]

Supplementary material to the article
Title: Diversity drivers of inland saline vegetation – what unites them and divides them?
Authors: Zuzana Dítě*, Róbert Šuvada, Tibor Tóth & Daniel Dítě
Journal: Ecology and Evolution

Electronic Appendix 2: Descriptive statistics of 19 climate factors for all subregions, boxplots showing these values with the indicated ANOVA result, and statistically significant differences among groups using ANOVA and the Unequal N HSD test (alpha = 0.05).

Macroregions			North German Plain			Pannonian Lowland						Transylvanian Basin			
Subregions			Thüringen	Sachsen-Anhalt	Kujawy	Jižní Morava	Seewinkel	Podunajská nížina	Dunántúl	Východo-slovenská nížina	Alföld	Câmpia Transilvaniei	Harghita	Mostecká pánev	Spiš
Cluster Number			1	2	3	4	5	6	7	8	9	10	11	12	13
WorldClim2		Mean / Min, Max / Std. Dev.													
Bio1	Annual Mean Temperature	Mean	**8.45**	**9.08**	**8.43**	**9.57**	**10.32**	**10.29**	**10.79**	**9.55**	**10.62**	**8.53**	**6.33**	**9.06**	**6.75**
		Min	8.26	8.55	8.14	8.50	10.13	9.99	10.55	9.21	10.09	7.90	4.54	7.09	6.37
		Max	8.69	9.58	8.65	9.96	10.41	10.73	10.92	9.73	11.52	8.95	7.48	9.79	7.33
		Std. Dev.	0.15	0.22	0.14	0.28	0.08	0.24	0.08	0.10	0.28	0.21	0.69	0.38	0.40
Bio2	Mean Diurnal Range	Mean	**8.38**	**8.47**	**8.50**	**8.94**	**9.05**	**10.01**	**8.38**	**9.81**	**10.13**	**10.25**	**11.47**	**8.70**	**9.74**
		Min	8.18	8.03	8.08	7.95	7.63	9.33	7.01	9.37	8.77	9.64	10.72	7.62	9.51
		Max	8.62	8.84	8.69	9.33	9.54	10.24	9.20	10.06	10.92	10.57	12.01	9.43	9.90
		Std. Dev.	0.15	0.19	0.13	0.24	0.55	0.25	0.51	0.14	0.65	0.25	0.36	0.32	0.18
Bio3	Isothermality	Mean	**32.70**	**32.46**	**29.68**	**30.54**	**30.54**	**32.64**	**28.68**	**31.42**	**32.07**	**32.60**	**34.82**	**31.48**	**32.71**
		Min	32.13	31.09	28.60	27.79	27.29	30.72	25.05	30.55	29.24	31.64	33.32	29.67	32.00
		Max	33.41	33.37	30.60	31.50	31.59	33.44	31.23	31.97	33.94	33.36	35.71	33.73	33.37
		Std. Dev.	0.45	0.51	0.54	0.62	1.20	0.66	1.37	0.32	1.34	0.52	0.67	0.85	0.68
Bio4	Temperature Seasonality	Mean	**652.09**	**658.16**	**766.26**	**776.30**	**770.24**	**788.41**	**798.32**	**814.43**	**821.31**	**818.83**	**828.61**	**721.51**	**760.93**
		Min	646.64	641.60	740.77	749.36	759.87	774.04	776.61	808.65	790.93	795.55	796.29	693.77	751.04
		Max	657.35	675.13	780.52	785.53	779.24	818.49	806.11	819.59	870.45	832.58	852.93	732.75	778.16
		Std. Dev.	4.03	6.28	8.45	6.05	6.73	13.37	5.88	2.77	16.69	7.45	11.88	5.48	10.99
Bio5	Max Temperature of Warmest Month	Mean	**22.77**	**23.83**	**24.22**	**25.48**	**26.20**	**26.92**	**26.30**	**25.93**	**27.54**	**24.62**	**23.25**	**24.24**	**22.23**
		Min	22.47	22.92	23.48	24.09	25.01	26.32	25.34	25.26	26.52	23.42	20.88	21.24	21.81
		Max	23.08	24.41	24.60	26.10	26.81	27.96	26.70	26.21	28.48	25.37	24.61	24.90	22.95
		Std. Dev.	0.24	0.33	0.27	0.42	0.57	0.46	0.31	0.15	0.50	0.38	0.86	0.47	0.45
Bio6	Min Temperature of Coldest Month	Mean	**-2.85**	**-2.26**	**-4.42**	**-3.80**	**-3.40**	**-3.75**	**-2.92**	**-5.28**	**-4.03**	**-6.81**	**-9.69**	**-3.37**	**-7.55**
		Min	-3.00	-2.60	-5.03	-4.17	-3.51	-4.02	-3.19	-5.45	-4.80	-6.99	-10.80	-4.39	-7.86
		Max	-2.64	-1.86	-4.08	-3.50	-2.93	-3.24	-2.64	-5.10	-2.90	-6.48	-8.80	-2.53	-7.19
		Std. Dev.	0.14	0.23	0.20	0.18	0.15	0.21	0.14	0.11	0.56	0.13	0.56	0.33	0.29
Bio7	Temperature Annual Range	Mean	**25.62**	**26.09**	**28.64**	**29.28**	**29.61**	**30.67**	**29.21**	**31.21**	**31.57**	**31.43**	**32.94**	**27.62**	**29.78**
		Min	25.32	25.36	27.74	28.26	27.97	29.75	27.99	30.58	30.00	30.23	31.68	25.63	29.65
		Max	25.80	26.61	29.43	29.71	30.31	31.74	29.74	31.45	32.98	31.92	33.88	28.17	30.15
		Std. Dev.	0.17	0.29	0.32	0.29	0.70	0.51	0.43	0.15	0.88	0.37	0.51	0.37	0.20
Bio8	Mean Temperature of Wettest Quarter	Mean	**15.14**	**16.58**	**17.82**	**18.15**	**18.13**	**18.82**	**19.31**	**18.65**	**19.27**	**16.79**	**14.84**	**17.90**	**14.46**
		Min	14.96	15.01	17.29	17.09	17.88	18.11	18.91	17.74	18.41	15.91	13.10	15.59	14.00
		Max	15.32	17.91	18.20	19.44	18.30	19.80	20.45	19.48	20.90	17.34	16.02	18.61	15.18
		Std. Dev.	0.13	0.85	0.21	0.75	0.15	0.59	0.57	0.68	0.73	0.28	0.70	0.41	0.48
Bio9	Mean Temperature of Driest Quarter	Mean	**4.08**	**2.20**	**0.04**	**1.40**	**2.34**	**2.10**	**2.44**	**0.95**	**1.99**	**-0.18**	**-2.66**	**1.27**	**-1.43**
		Min	3.87	1.59	-0.16	-0.27	2.11	1.89	2.16	0.70	1.23	-0.60	-4.27	-0.97	-1.71
		Max	4.32	4.93	0.18	1.86	2.47	2.44	2.55	1.15	3.13	0.15	-1.56	4.63	-1.03
		Std. Dev.	0.16	0.75	0.09	0.46	0.07	0.15	0.10	0.09	0.43	0.15	0.65	1.71	0.28
Bio10	Mean Temperature of Warmest Quarter	Mean	**16.60**	**17.19**	**17.82**	**19.02**	**19.73**	**19.82**	**20.43**	**19.21**	**20.45**	**18.10**	**16.18**	**17.90**	**15.86**
		Min	16.44	16.48	17.29	17.70	19.47	19.34	19.98	18.81	19.75	17.24	14.08	15.59	15.40
		Max	16.78	17.91	18.20	19.53	19.93	20.65	20.54	19.48	21.13	18.69	17.44	18.61	16.57
		Std. Dev.	0.12	0.31	0.21	0.35	0.18	0.41	0.11	0.13	0.27	0.28	0.79	0.41	0.48
Bio11	Mean Temperature of Coldest Quarter	Mean	**0.58**	**1.08**	**-1.08**	**-0.12**	**0.82**	**0.42**	**0.82**	**-0.85**	**0.27**	**-2.06**	**-4.27**	**0.18**	**-2.89**
		Min	0.35	0.78	-1.50	-0.73	0.66	0.22	0.65	-1.12	-0.67	-2.34	-5.55	-1.42	-3.09
		Max	0.88	1.47	-0.88	0.15	0.95	0.81	0.92	-0.71	1.49	-1.79	-3.36	1.01	-2.63
		Std. Dev.	0.19	0.18	0.14	0.20	0.05	0.14	0.07	0.07	0.46	0.12	0.54	0.38	0.19
Bio12	Annual Precipitation	Mean	**692.30**	**514.27**	**521.71**	**534.94**	**575.25**	**534.44**	**543.81**	**631.09**	**540.05**	**600.00**	**601.59**	**608.57**	**669.07**
		Min	669.00	477.00	510.00	497.00	571.00	514.00	522.00	583.00	497.00	583.00	568.00	505.00	625.00
		Max	717.00	589.00	531.00	603.00	581.00	552.00	626.00	734.00	630.00	632.00	629.00	819.00	718.00
		Std. Dev.	19.09	23.39	4.33	31.81	2.93	8.89	27.12	32.76	25.16	13.40	19.50	65.43	36.51
Bio13	Precipitation of Wettest Month	Mean	**73.00**	**62.23**	**77.58**	**77.39**	**70.38**	**62.15**	**60.36**	**79.77**	**69.59**	**93.09**	**94.36**	**84.25**	**100.07**
		Min	71.00	54.00	75.00	73.00	68.00	57.00	56.00	76.00	58.00	92.00	88.00	74.00	95.00
		Max	75.00	73.00	80.00	85.00	72.00	67.00	78.00	86.00	86.00	96.00	100.00	106.00	106.00
		Std. Dev.	1.41	6.23	1.19	3.40	0.96	2.54	5.82	2.75	6.67	1.04	4.03	6.75	4.25
Bio14	Precipitation of Driest Month	Mean	**44.20**	**28.47**	**22.15**	**24.80**	**28.36**	**28.06**	**26.44**	**32.75**	**28.47**	**24.96**	**24.13**	**31.65**	**27.07**
		Min	41.00	24.00	21.00	22.00	27.00	27.00	26.00	29.00	24.00	24.00	23.00	21.00	25.00
		Max	47.00	35.00	23.00	29.00	31.00	29.00	27.00	41.00	36.00	28.00	25.00	49.00	29.00
		Std. Dev.	2.35	2.45	0.45	1.93	0.86	0.57	0.50	2.70	1.96	1.19	0.80	6.45	1.64

Bio15	Precipitation Seasonality	**14.61**		**25.58**		**38.54**		**40.84**		**28.59**		**25.32**		**25.74**		**29.65**		**27.52**		**44.16**		**50.21**		**35.96**		**42.52**	
		13.44	15.92	20.44	32.01	36.55	40.47	37.29	43.06	26.39	29.94	23.76	26.96	23.67	33.69	23.73	35.15	23.93	32.96	39.02	46.58	48.62	52.93	25.10	47.58	41.29	44.22
		0.84		3.50		0.77		1.28		0.96		0.97		2.40		2.45		2.37		2.04		0.86		5.92		1.24	
Bio16	Precipitation of Wettest Quarter	**203.10**		**172.47**		**203.93**		**212.15**		**194.74**		**175.72**		**170.54**		**220.90**		**183.13**		**247.30**		**258.05**		**230.95**		**267.29**	
		199.00	205.00	162.00	190.00	199.00	210.00	199.00	240.00	190.00	199.00	164.00	185.00	160.00	225.00	209.00	242.00	162.00	220.00	243.00	254.00	244.00	269.00	208.00	282.00	254.00	284.00
		2.28		7.10		2.50		12.26		2.49		5.07		15.95		8.33		13.32		2.51		7.91		14.84		11.96	
Bio17	Precipitation of Driest Quarter	**145.10**		**96.30**		**77.86**		**79.54**		**93.13**		**88.93**		**84.98**		**102.23**		**89.89**		**82.09**		**77.59**		**105.67**		**94.14**	
		138.00	152.00	84.00	114.00	75.00	82.00	72.00	90.00	91.00	98.00	86.00	92.00	82.00	90.00	89.00	134.00	72.00	115.00	77.00	94.00	73.00	83.00	70.00	170.00	84.00	104.00
		5.59		7.14		1.22		5.19		1.35		1.19		1.59		9.65		7.36		4.52		2.77		21.83		8.08	
Bio18	Precipitation of Warmest Quarter	**196.70**		**171.13**		**203.93**		**211.38**		**193.55**		**173.42**		**169.27**		**220.72**		**180.32**		**236.39**		**254.03**		**230.95**		**262.36**	
		194.00	199.00	162.00	188.00	199.00	210.00	197.00	240.00	190.00	197.00	164.00	185.00	158.00	225.00	209.00	241.00	158.00	217.00	232.00	243.00	240.00	269.00	208.00	282.00	246.00	281.00
		1.83		7.09		2.50		12.91		2.05		6.39		16.62		8.22		13.26		2.69		8.27		14.84		13.64	
Bio19	Precipitation of Coldest Quarter	**164.70**		**99.93**		**87.75**		**81.98**		**95.51**		**101.77**		**98.98**		**117.35**		**103.52**		**92.39**		**82.33**		**105.80**		**99.93**	
		152.00	176.00	87.00	119.00	85.00	91.00	73.00	94.00	93.00	100.00	99.00	106.00	96.00	102.00	101.00	158.00	81.00	133.00	86.00	108.00	78.00	88.00	70.00	172.00	87.00	112.00
		8.86		8.38		1.09		6.34		1.95		1.51		1.12		12.76		7.87		6.10		3.19		22.01		10.33	

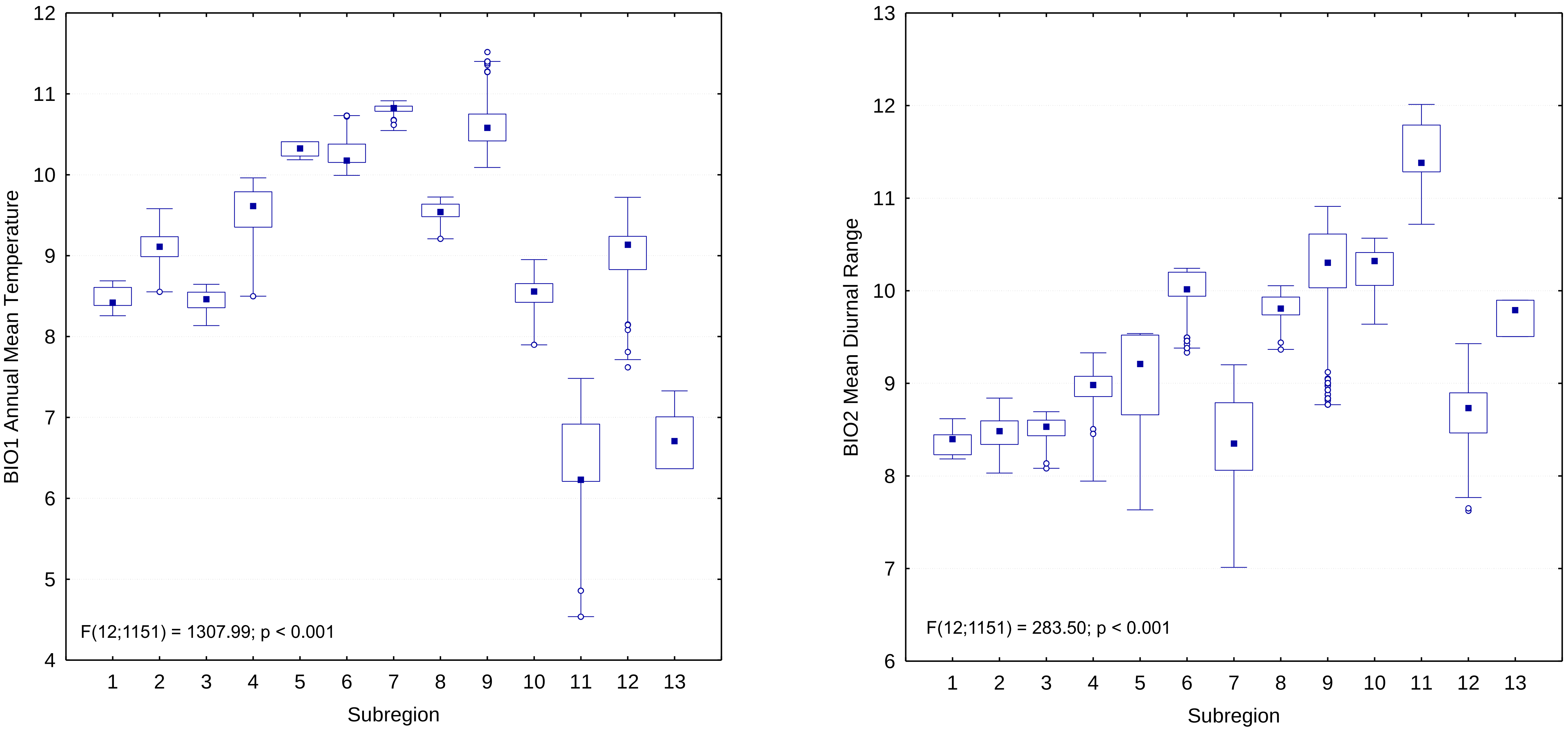

BIO1 Annual Mean Temperature
F(12;1151) = 1307.99; p < 0.001
Subregion
BIO2 Mean Diurnal Range
F(12;1151) = 283.50; p < 0.001
Subregion

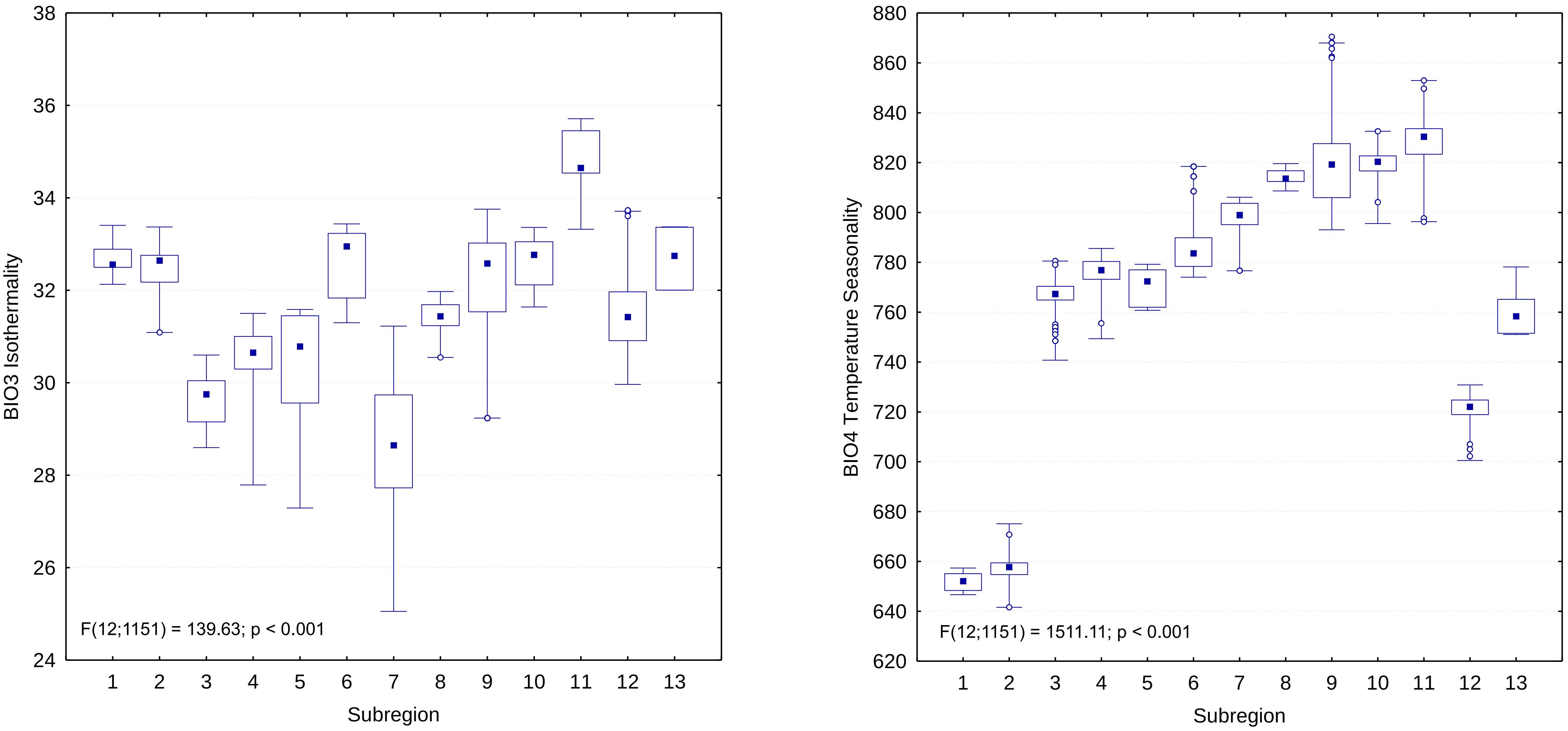

BIO3 Isothermality
Subregion
F(12;1151) = 139.63; p < 0.001
BIO4 Temperature Seasonality
Subregion
F(12;1151) = 1511.11; p < 0.001

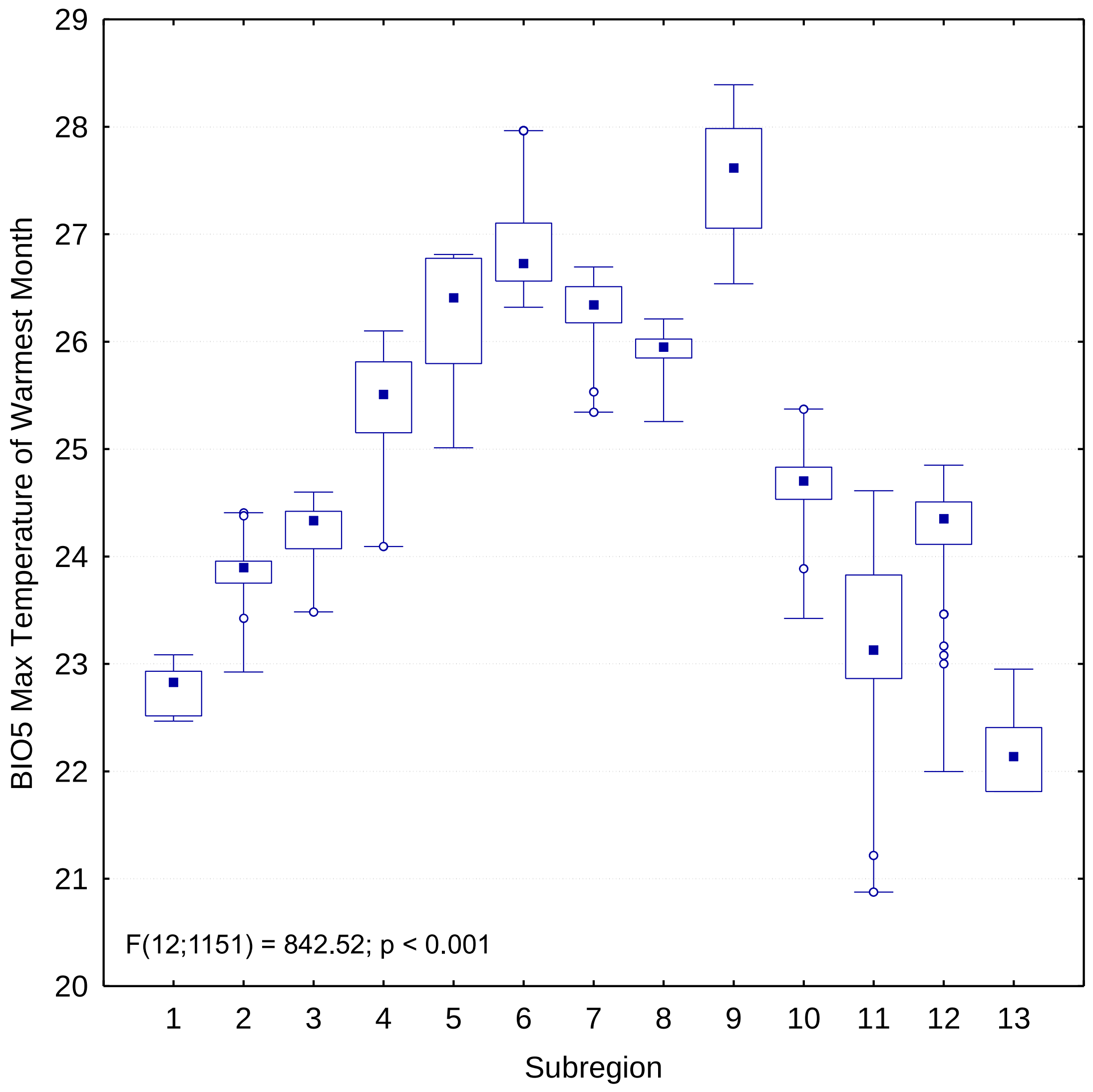
BIO5 Max Temperature of Warmest Month
Subregion
F(12;1151) = 842.52; p < 0.001
29
28
27
26
25
24
23
22
21
20
1 2 3 4 5 6 7 8 9 10 11 12 13

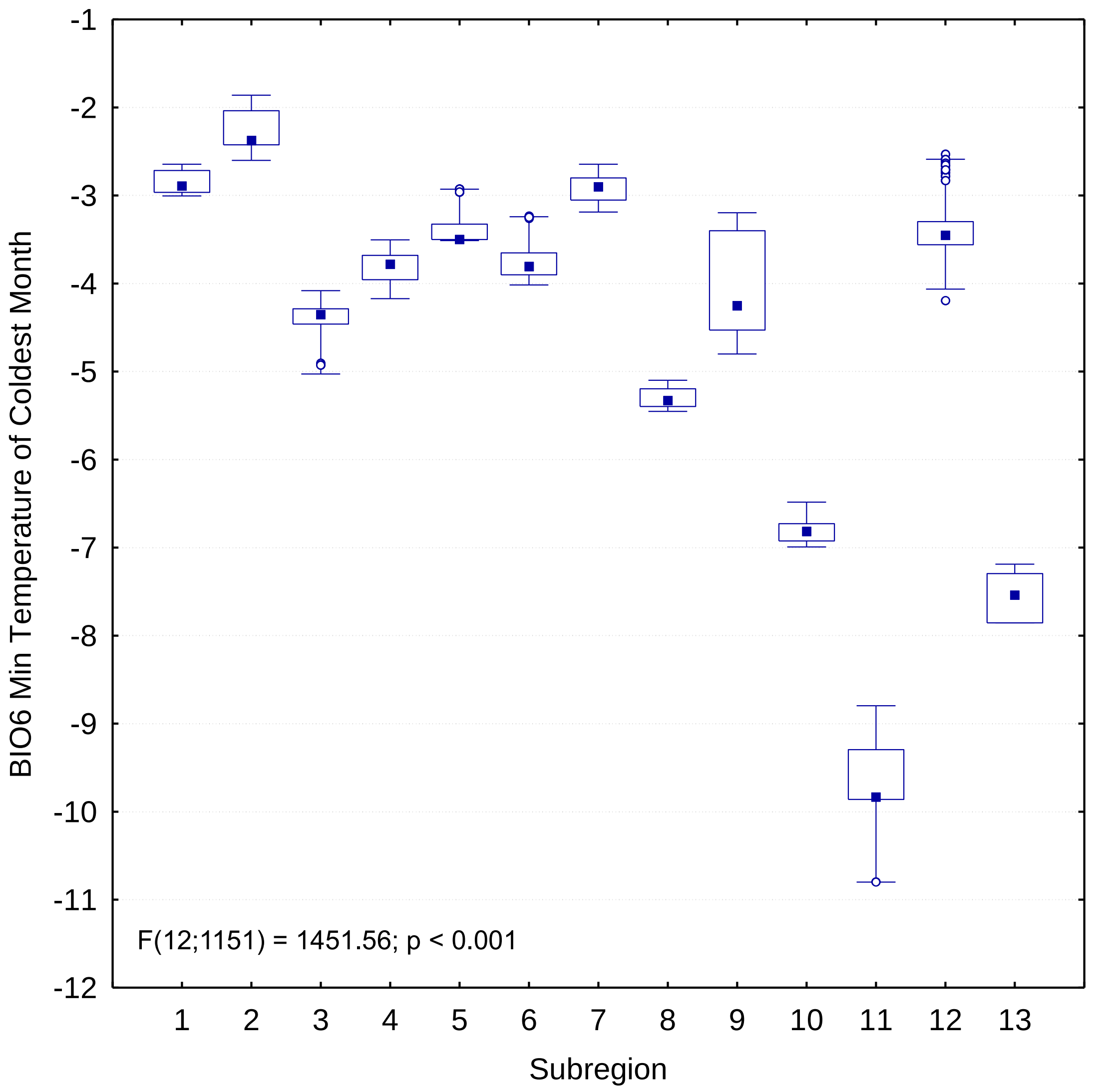
BIO6 Min Temperature of Coldest Month
Subregion
F(12;1151) = 1451.56; p < 0.001
-1
-2
-3
-4
-5
-6
-7
-8
-9
-10
-11
-12
1 2 3 4 5 6 7 8 9 10 11 12 13

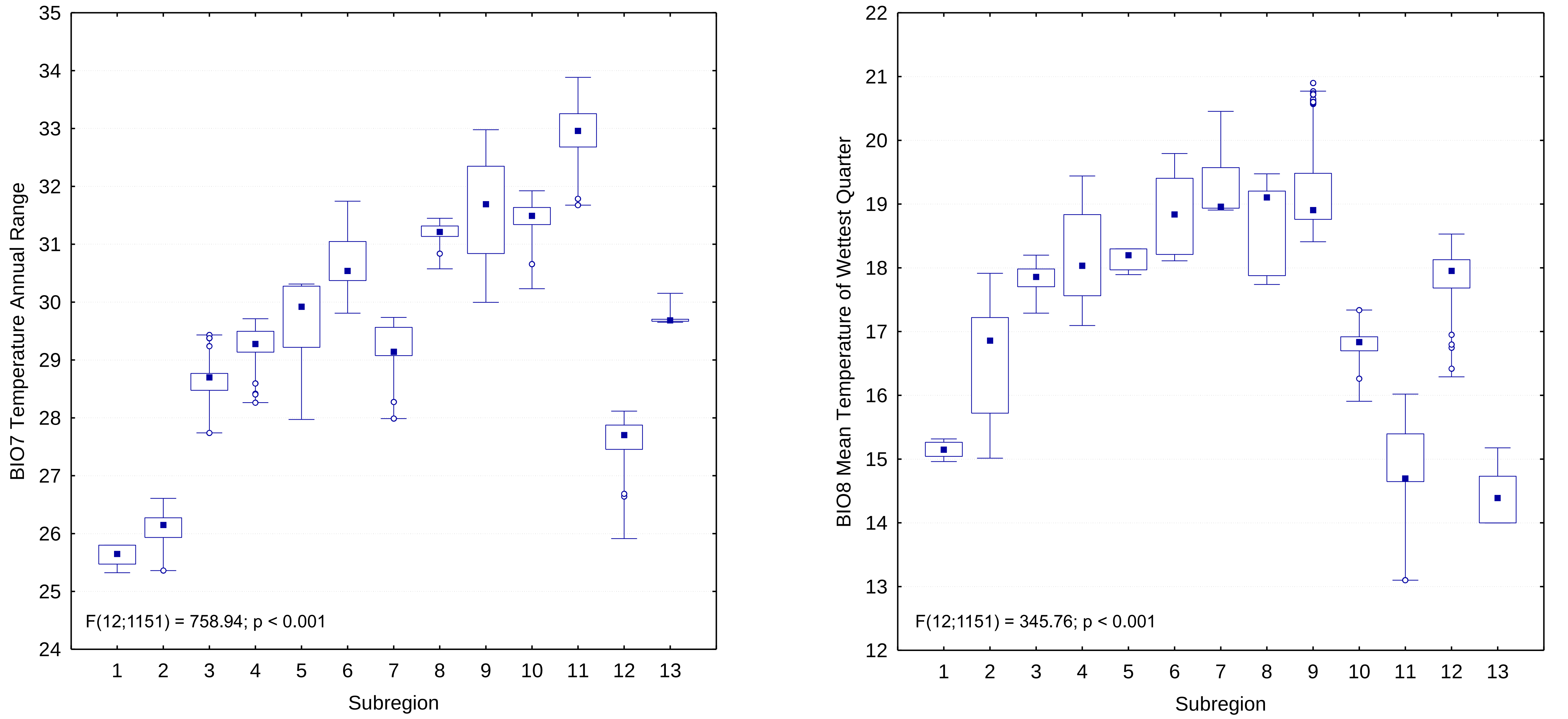
BIO7 Temperature Annual Range
F(12;1151) = 758.94; p < 0.001
Subregion
BIO8 Mean Temperature of Wettest Quarter
F(12;1151) = 345.76; p < 0.001
Subregion

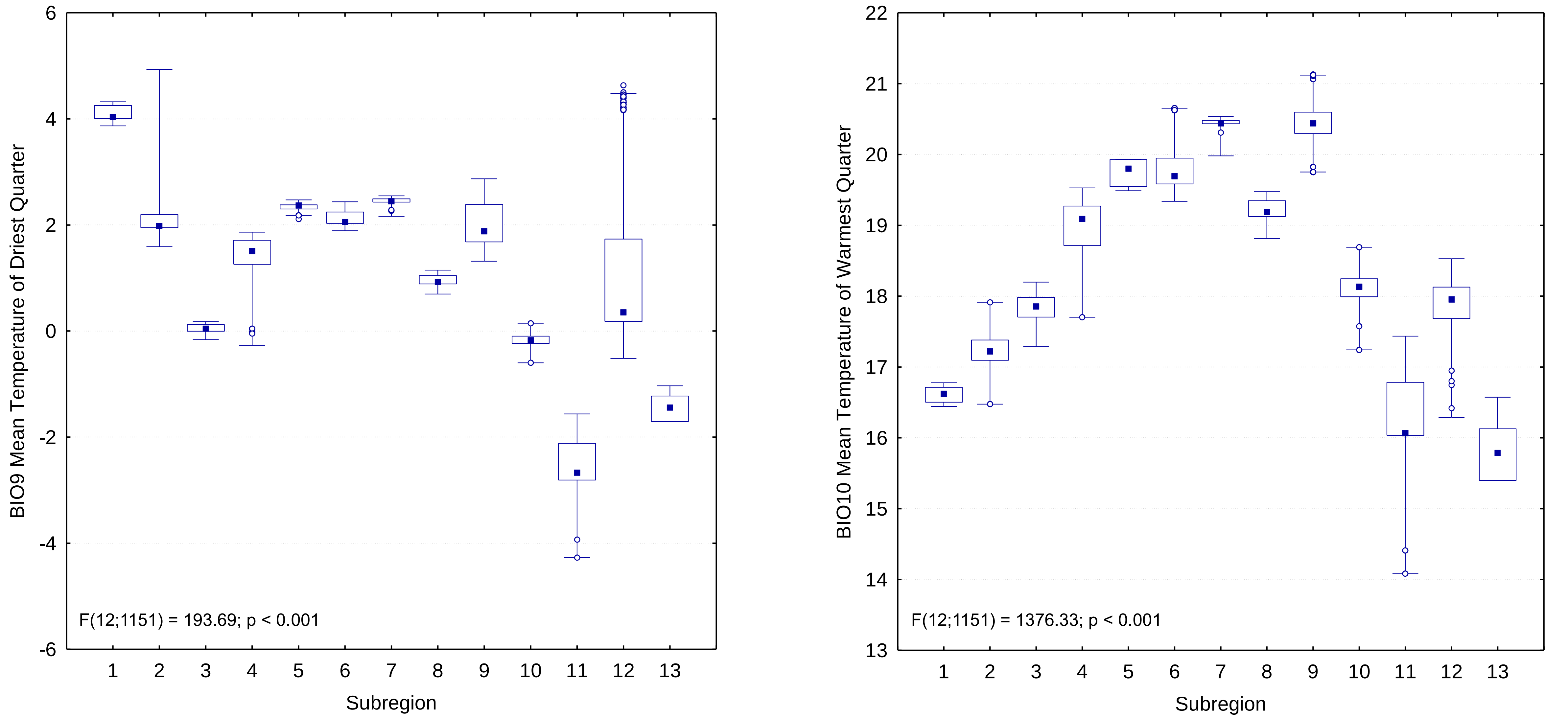

BIO9 Mean Temperature of Driest Quarter
F(12;1151) = 193.69; p < 0.001
Subregion
BIO10 Mean Temperature of Warmest Quarter
F(12;1151) = 1376.33; p < 0.001
Subregion

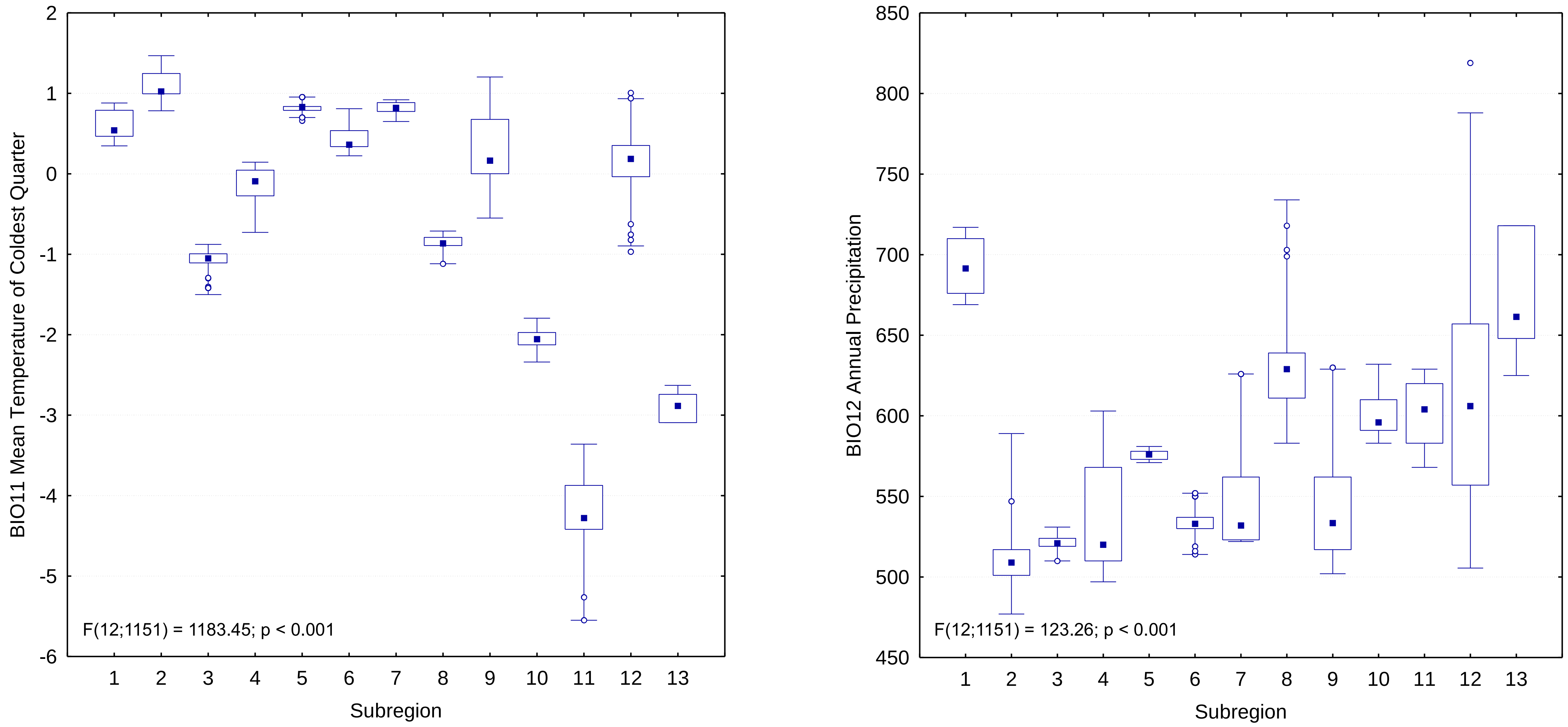

BIO11 Mean Temperature of Coldest Quarter
Subregion
F(12;1151) = 1183.45; p < 0.001
BIO12 Annual Precipitation
Subregion
F(12;1151) = 123.26; p < 0.001

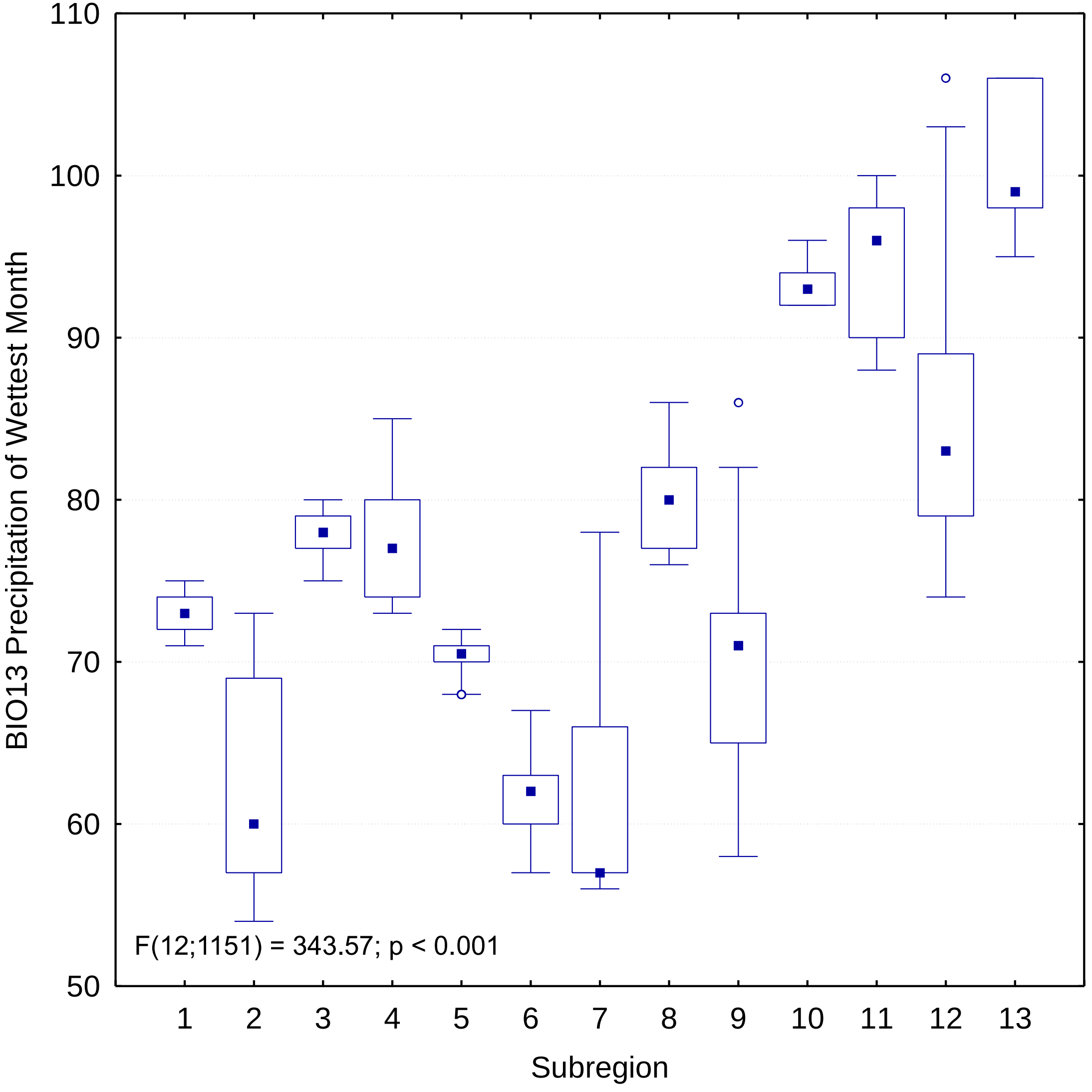
BIO13 Precipitation of Wettest Month
110
100
90
80
70
60
50
1 2 3 4 5 6 7 8 9 10 11 12 13
Subregion
F(12;1151) = 343.57; p < 0.001

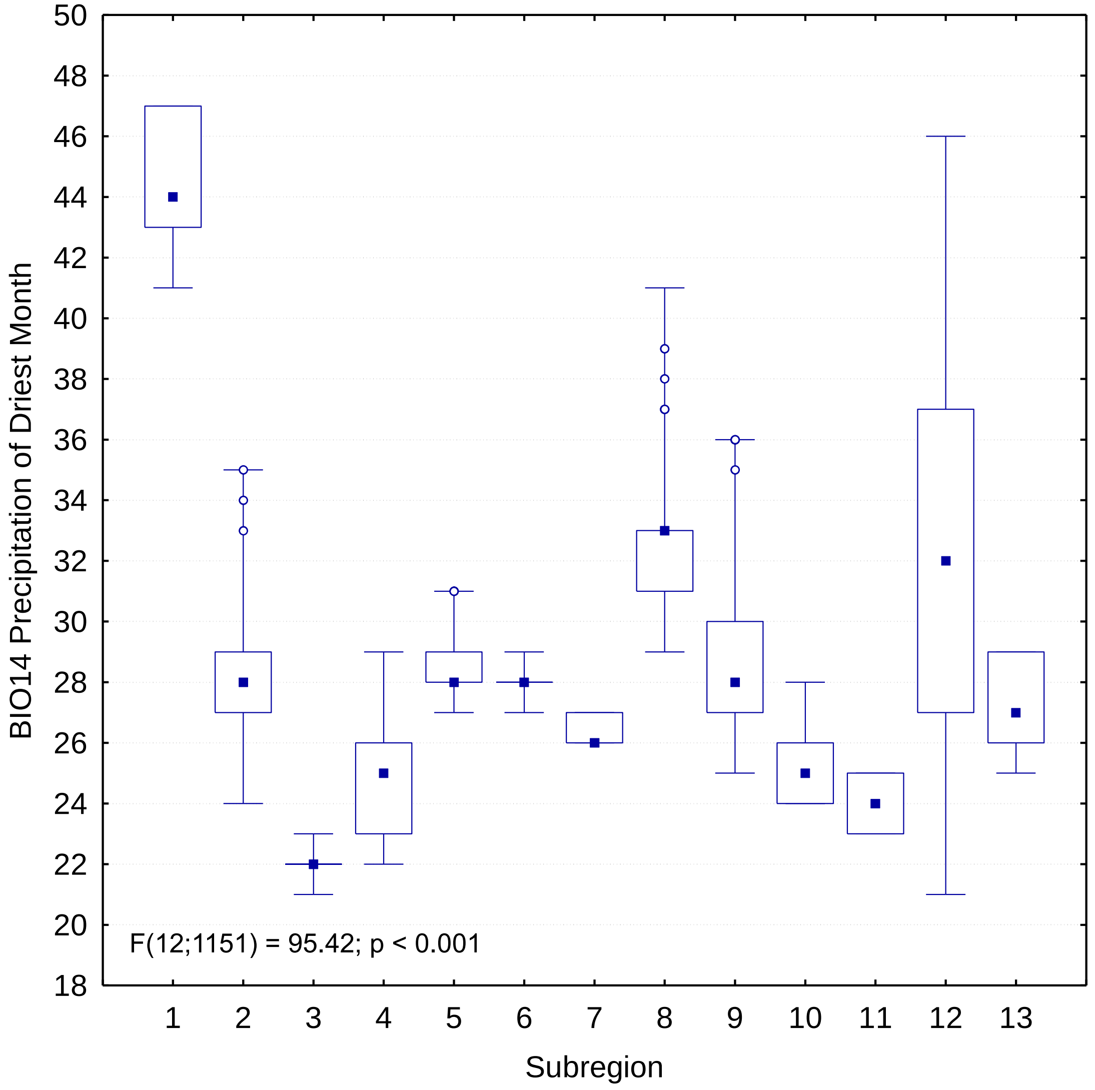
BIO14 Precipitation of Driest Month
50
48
46
44
42
40
38
36
34
32
30
28
26
24
22
20
18
1 2 3 4 5 6 7 8 9 10 11 12 13
Subregion
F(12;1151) = 95.42; p < 0.001

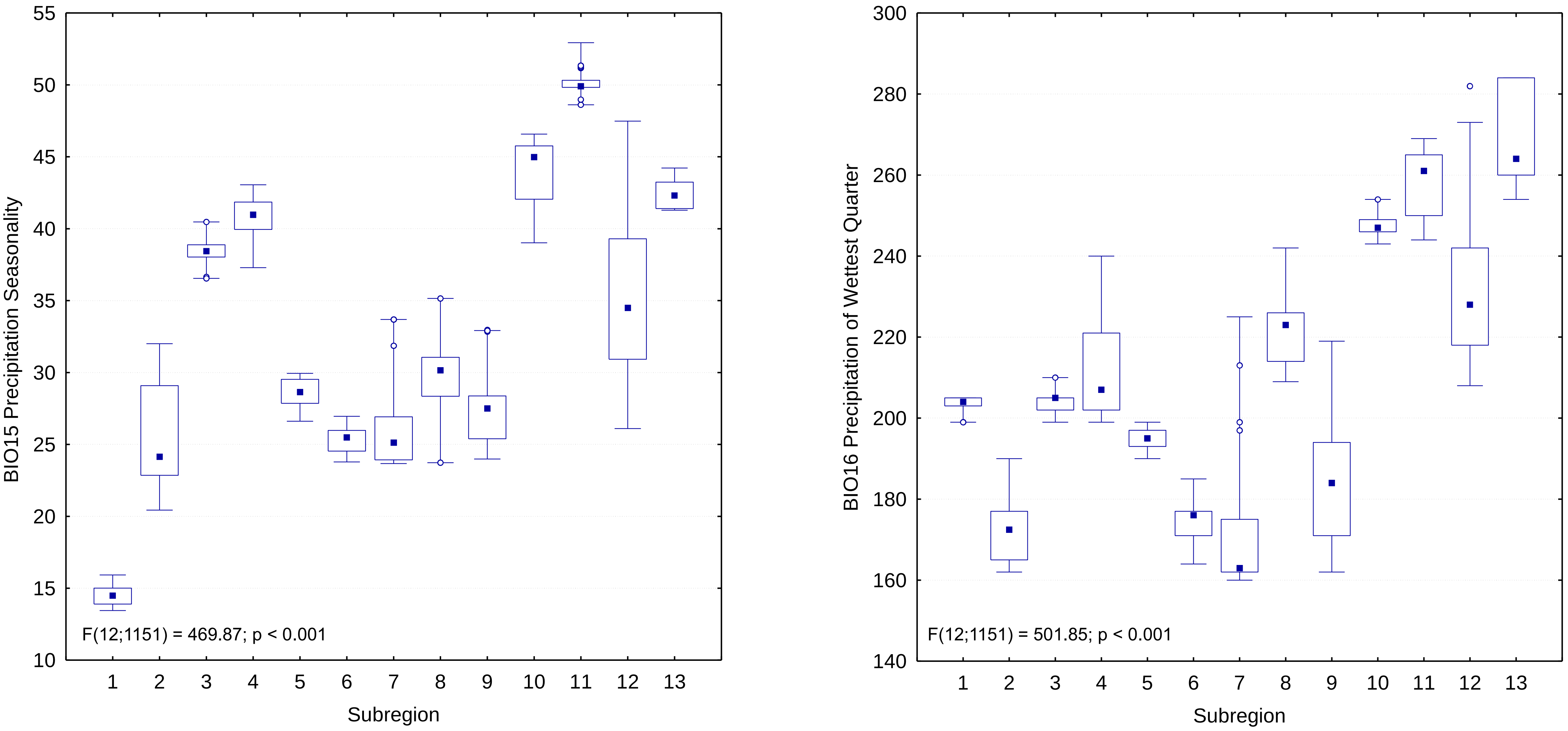

BIO15 Precipitation Seasonality
F(12;1151) = 469.87; p < 0.001
Subregion
BIO16 Precipitation of Wettest Quarter
F(12;1151) = 501.85; p < 0.001
Subregion

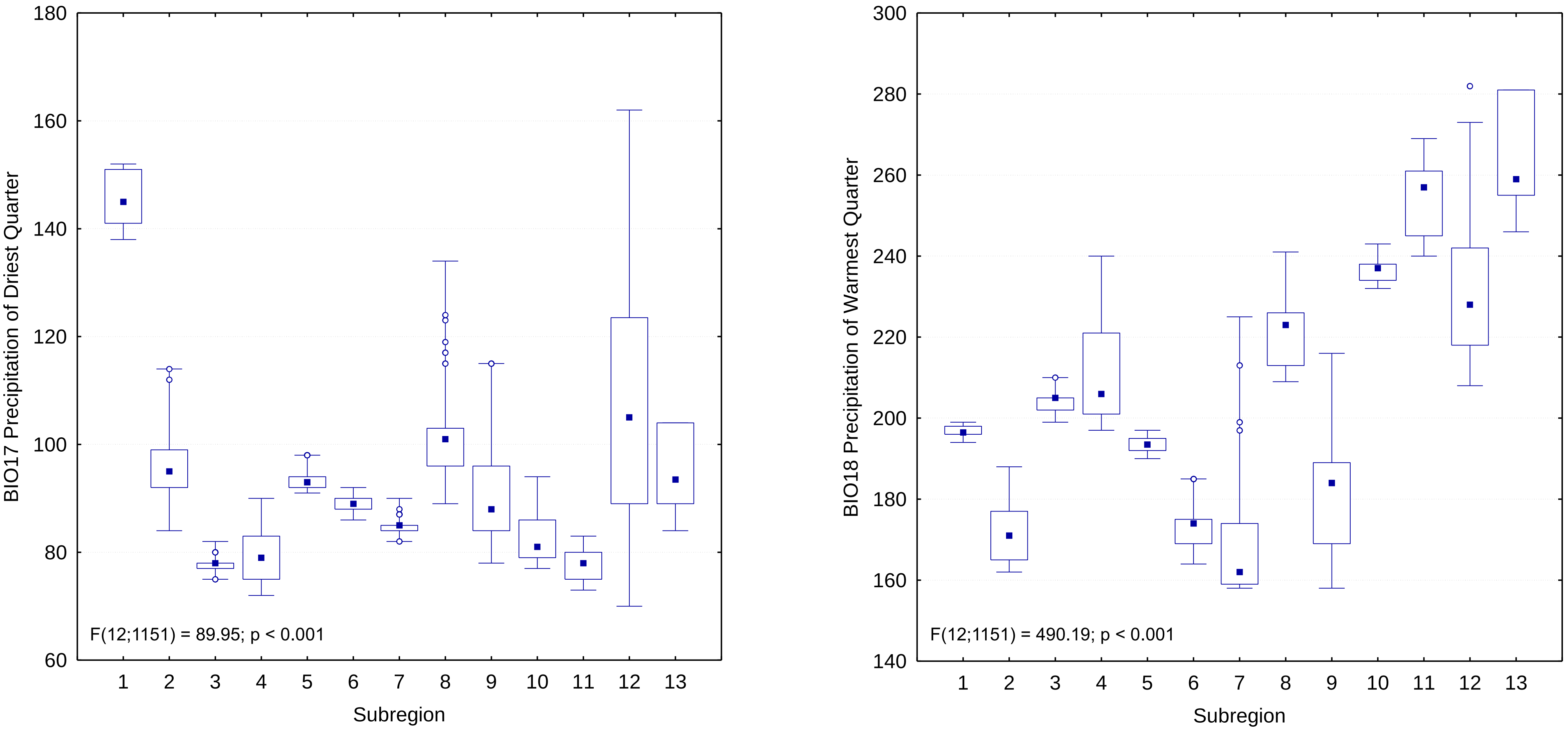

BIO17 Precipitation of Driest Quarter
F(12;1151) = 89.95; p < 0.001
Subregion
BIO18 Precipitation of Warmest Quarter
F(12;1151) = 490.19; p < 0.001
Subregion

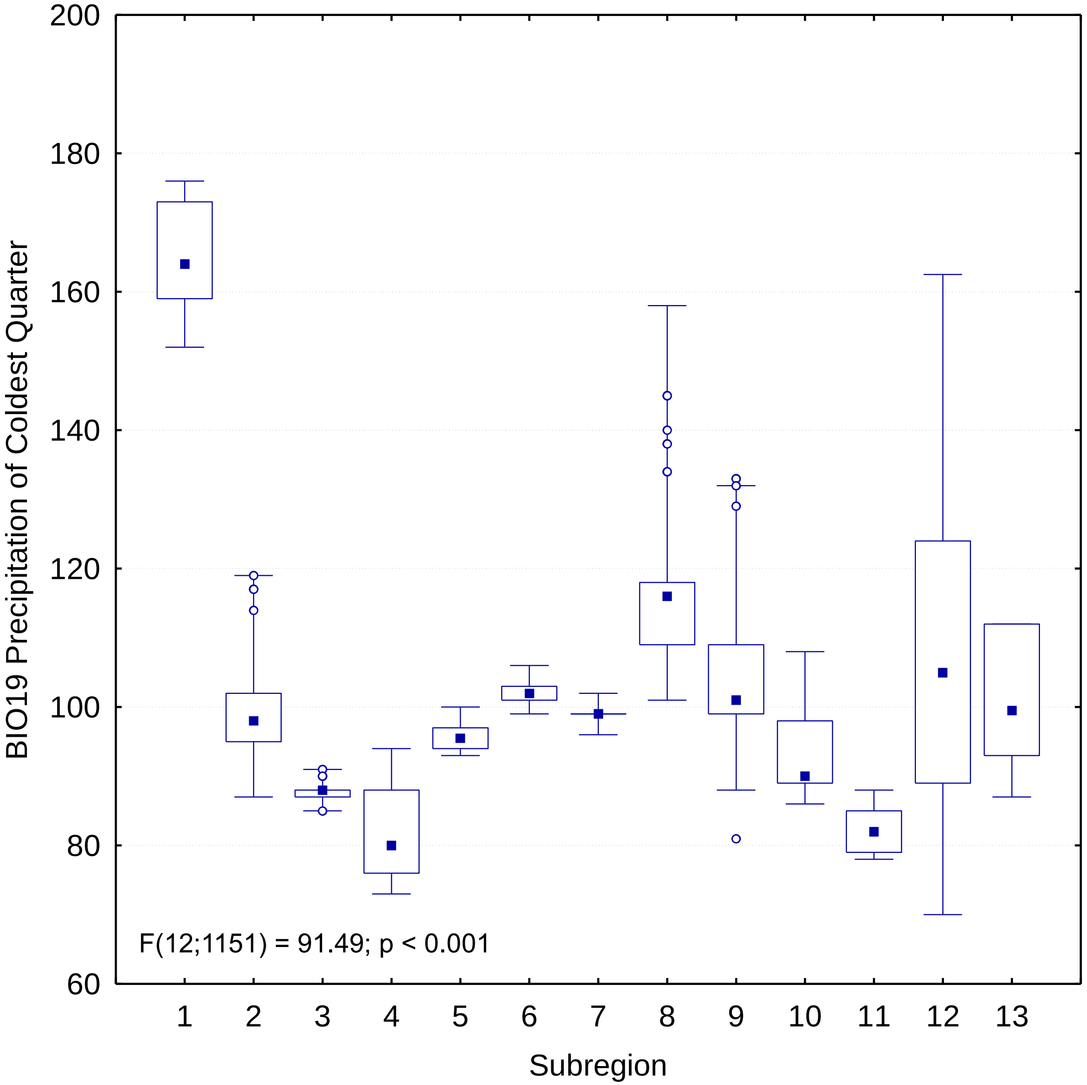

BIO19 Precipitation of Coldest Quarter
200
180
160
140
120
100
80
60
F(12;1151) = 91.49; p < 0.001
1
2
3
4
5
6
7
8
9
10
11
12
13
Subregion

Statistically significant differences among groups using ANOVA and the Unequal N HSD test (alpha = 0.05).

Cluster	Subregion	BIO1 Mean	BIO1 group	BIO2 Mean	BIO2 Group	BIO3 Mean	BIO3 Group	BIO4 Mean	BIO4 Group	BIO5 Mean	BIO5 Group	BIO6 Mean	BIO6 Group	BIO7 Mean	BIO7 Group
1	Thüringen	8.45	c	8.38	abc	32.70	defg	652.09	a	22.77	ab	-2.85	i	25.62	a
2	Sachsen-Anhalt	9.08	d	8.47	ab	32.46	ef	658.16	a	23.83	c	-2.26	j	26.09	a
3	Kujawy	8.43	c	8.50	ab	29.68	b	766.26	c	24.22	cd	-4.42	e	28.64	c
4	Jižní Morava	9.57	e	8.94	cd	30.54	c	776.30	d	25.48	e	-3.80	g	29.28	d
5	Seewinkel	10.32	f	9.05	d	30.54	c	770.24	c	26.20	g	-3.40	h	29.61	e
6	Podunajská nížina	10.29	f	10.01	ef	32.64	f	788.41	e	26.92	h	-3.75	g	30.67	f
7	Dunántúl	10.79	h	8.38	a	28.68	a	798.32	f	26.30	g	-2.92	i	29.21	d
8	Východoslovenská nížina	9.55	e	9.81	ef	31.42	d	814.43	g	25.93	f	-5.28	d	31.21	g
9	Alföld	10.62	g	10.13	f	32.07	e	821.31	h	27.54	i	-4.03	f	31.57	h
10	Câmpia Transilvaniei	8.53	c	10.25	f	32.60	ef	818.83	gh	24.62	d	-6.81	c	31.43	gh
11	Harghita	6.33	a	11.47	g	34.82	h	828.61	h	23.25	b	-9.69	a	32.94	i
12	Mostecká pánev	9.06	d	8.70	b	31.48	dg	721.51	b	24.24	d	-3.37	h	27.62	b
13	Spiš	6.75	b	9.74	ef	32.71	efg	760.93	c	22.23	a	-7.55	b	29.78	de

Cluster	Subregion	BIO8 Mean	BIO8 Group	BIO9 Mean	BIO9 Group	BIO10 Mean	BIO10 Group	BIO11 Mean	BIO11 Group	BIO12 Mean	BIO12 Group	BIO13 Mean	BIO13 Group
1	Thüringen	15.14	a	4.08	h	16.60	b	0.58	ghi	692.30	g	73.00	bc
2	Sachsen-Anhalt	16.58	b	2.20	fg	17.19	c	1.08	j	514.27	a	62.23	a
3	Kujawy	17.82	cd	0.04	c	17.82	d	-1.08	d	521.71	a	77.58	c
4	Jižní Morava	18.15	cd	1.40	e	19.02	e	-0.12	f	534.94	ab	77.39	c
5	Seewinkel	18.13	d	2.34	g	19.73	g	0.82	i	575.25	c	70.38	b
6	Podunajská nížina	18.82	e	2.10	fg	19.82	g	0.42	h	534.44	ab	62.15	a
7	Dunántúl	19.31	f	2.44	fg	20.43	h	0.82	i	543.81	b	60.36	a
8	Východoslovenská nížina	18.65	e	0.95	d	19.21	f	-0.85	e	631.09	ef	79.77	c
9	Alföld	19.27	f	1.99	f	20.45	h	0.27	g	540.05	ab	69.59	b
10	Câmpia Transilvaniei	16.79	b	-0.18	c	18.10	d	-2.06	c	600.00	cde	93.09	e
11	Harghita	14.84	a	-2.66	a	16.18	ab	-4.27	a	601.59	d	94.36	ef
12	Mostecká pánev	17.90	cd	1.27	de	17.90	d	0.18	g	608.57	d	84.25	d
13	Spiš	14.46	a	-1.43	b	15.86	a	-2.89	b	669.07	fg	100.07	f

Cluster	Subregion	BIO14 Mean	BIO14 Group	BIO15 Mean	BIO15 Group	BIO16 Mean	BIO16 Group	BIO17 Mean	BIO17 Group	BIO18 Mean	BIO18 Group	BIO19 Mean	BIO19 Group
1	Thüringen	44.20	g	14.61	a	203.10	cde	145.10	g	196.70	cde	164.70	h
2	Sachsen-Anhalt	28.47	de	25.58	bc	172.47	a	96.30	de	171.13	ab	99.93	def
3	Kujawy	22.15	a	38.54	f	203.93	d	77.86	a	203.93	d	87.75	ac
4	Jižní Morava	24.80	bc	40.84	g	212.15	e	79.54	ab	211.38	e	81.98	a
5	Seewinkel	28.36	e	28.59	d	194.74	c	93.13	d	193.55	c	95.51	b
6	Podunajská nížina	28.06	de	25.32	b	175.72	a	88.93	cd	173.42	a	101.77	def
7	Dunántúl	26.44	cd	25.74	bc	170.54	a	84.98	bc	169.27	a	98.98	bde
8	Východoslovenská nížina	32.75	f	29.65	d	220.90	f	102.23	ef	220.72	f	117.35	g
9	Alföld	28.47	e	27.52	c	183.13	b	89.89	cd	180.32	b	103.52	ef
10	Câmpia Transilvaniei	24.96	abc	44.16	h	247.30	h	82.09	abc	236.39	g	92.39	abcd
11	Harghita	24.13	ab	50.21	i	258.05	i	77.59	ab	254.03	h	82.33	a
12	Mostecká pánev	31.65	f	35.96	e	230.95	g	105.67	f	230.95	g	105.80	f
13	Spiš	27.07	bcde	42.52	gh	267.29	i	94.14	cdef	262.36	h	99.93	bcdef
