# Supplementary material for: Diversity Drivers of Inland Saline Vegetation—What Unites Them and Divides Them?
Source: Ecol Evol. 2025 May 14;15(5):e71249. doi: 10.1002/ece3.71249 (PMC12078054; doi:10.1002/ece3.71249)
Supplement: Supplementary file 3 — Appendix S3. Barchart plots for six variables (PCspec, PCassoc, PCclim, PCdiss, Area, Number of Species) showing the transformation of ordination scores and original values into rank categories. [file ECE3-15-e71249-s004.pdf]

Supplementary material to the article

Title: Diversity drivers of inland saline vegetation – what unites them and divides them?
Authors: Zuzana Dítě*, Róbert Šuvada, Tibor Tóth & Daniel Dítě
Journal: Ecology and Evolution

Electronic Appendix 3: Barchart plots for six variables (PCspec, PCassoc, PCclim, PCdiss, Area, Number of Species) showing the transformation of ordination scores and original values into rank categories.

PCspec (48.67 % of variance)

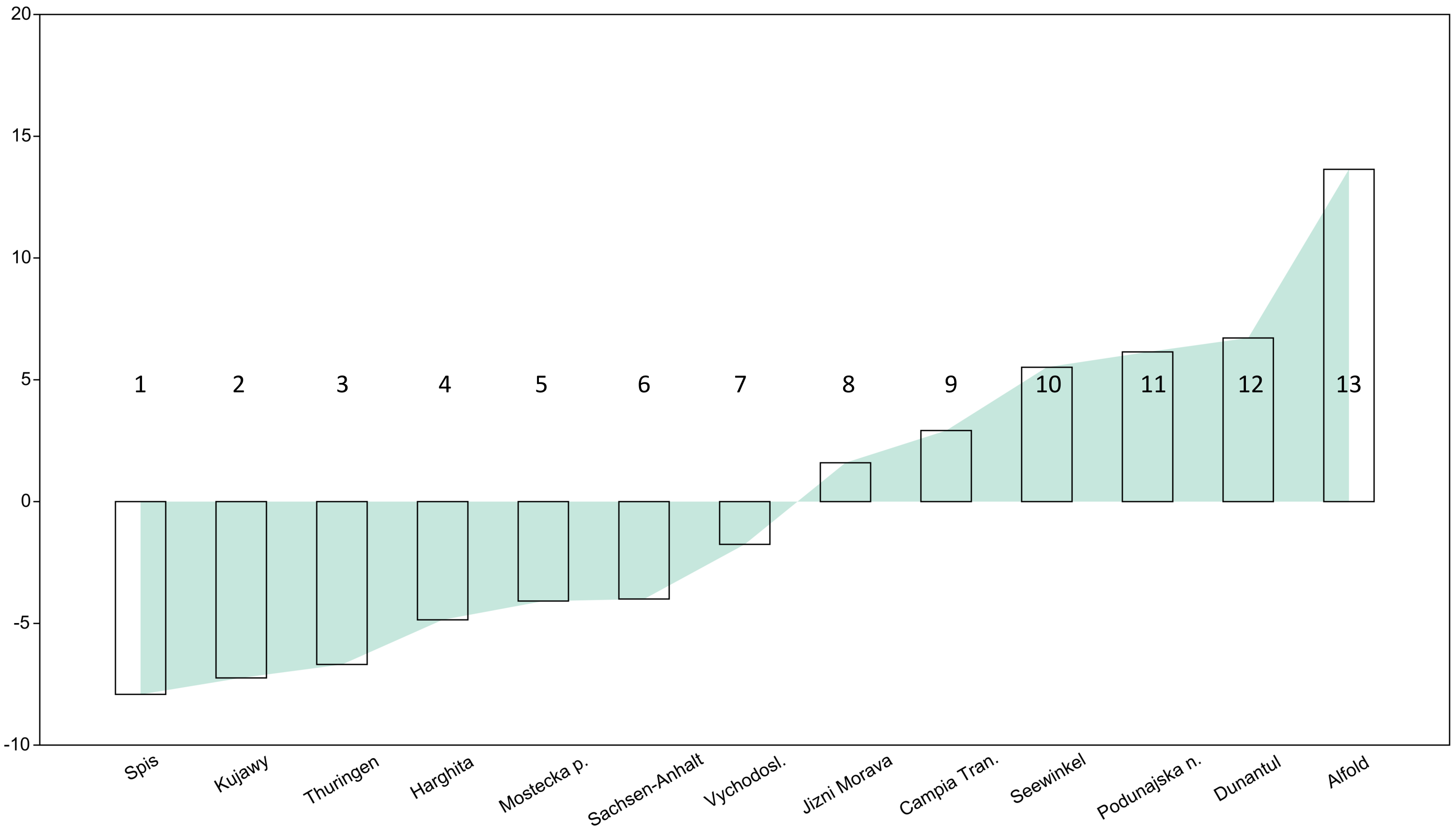

PCassoc (60.26 % of variance)

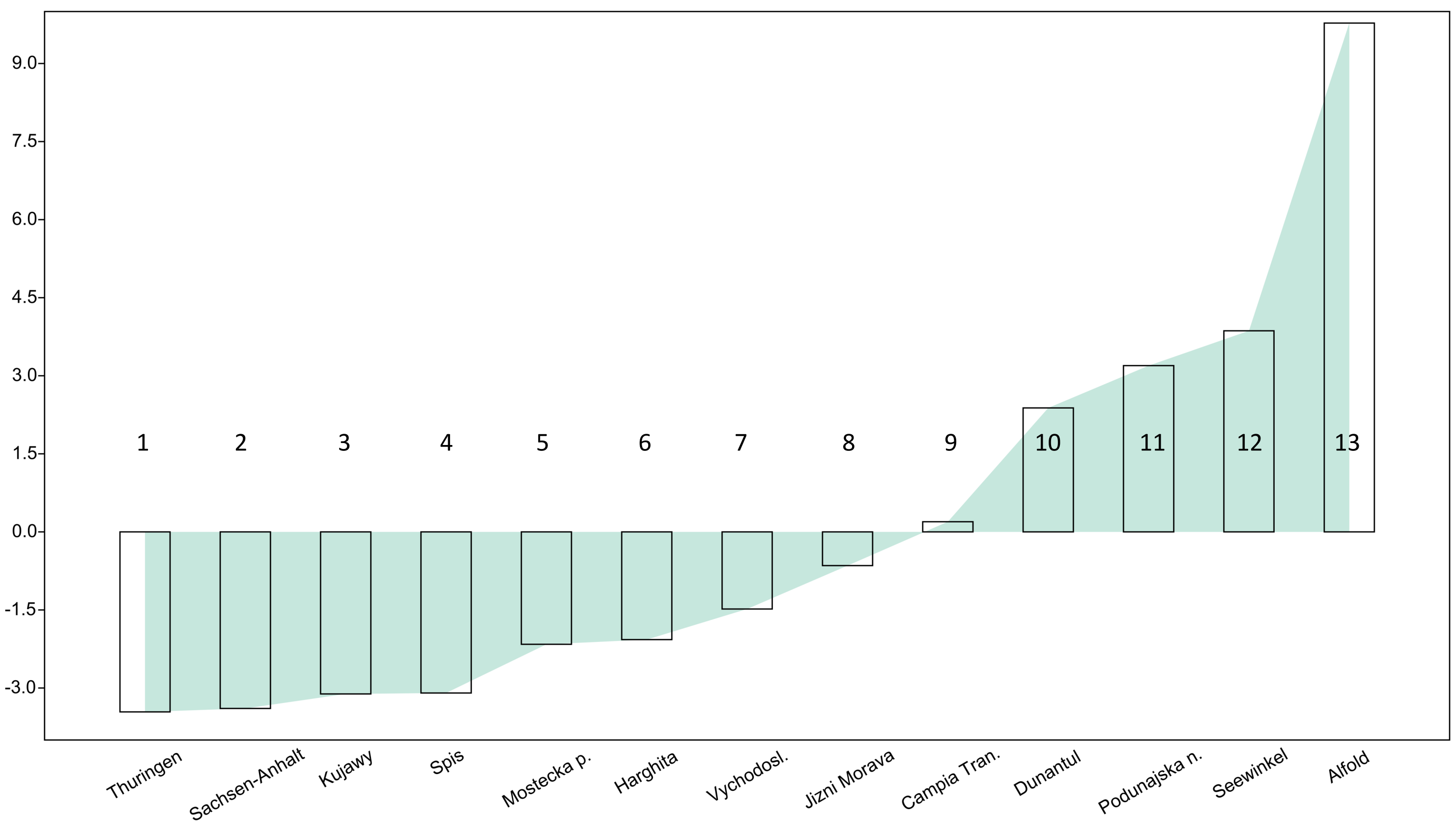

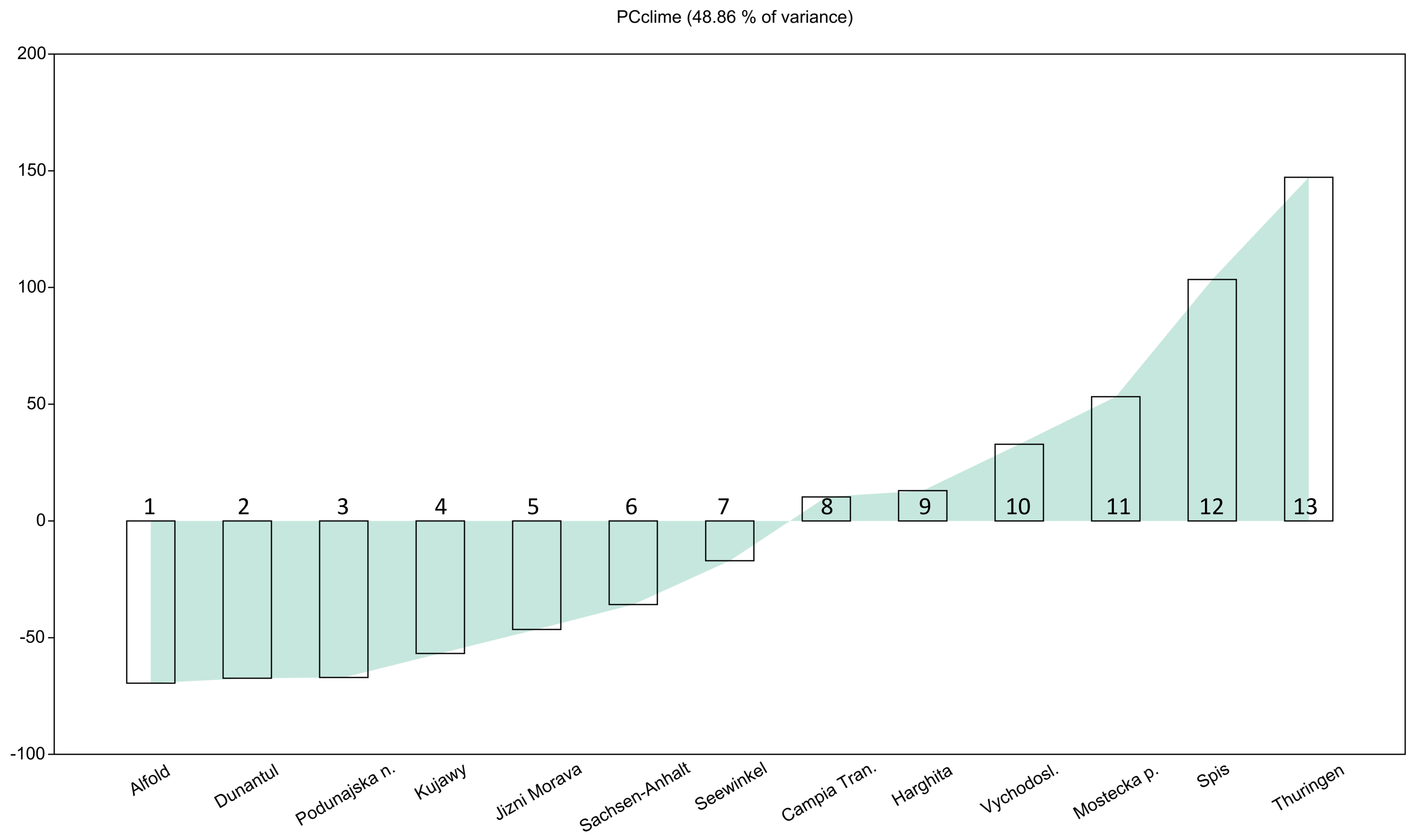
PCclime (48.86 % of variance)
200
150
100
50
0
-50
-100
1
2
3
4
5
6
7
8
9
10
11
12
13
Alfold
Dunantul
Podunajska n.
Kujawy
Jizni Morava
Sachsen-Anhalt
Seewinkel
Campia Tran.
Harghita
Vychodosl.
Mostecka p.
Spis
Thuringen

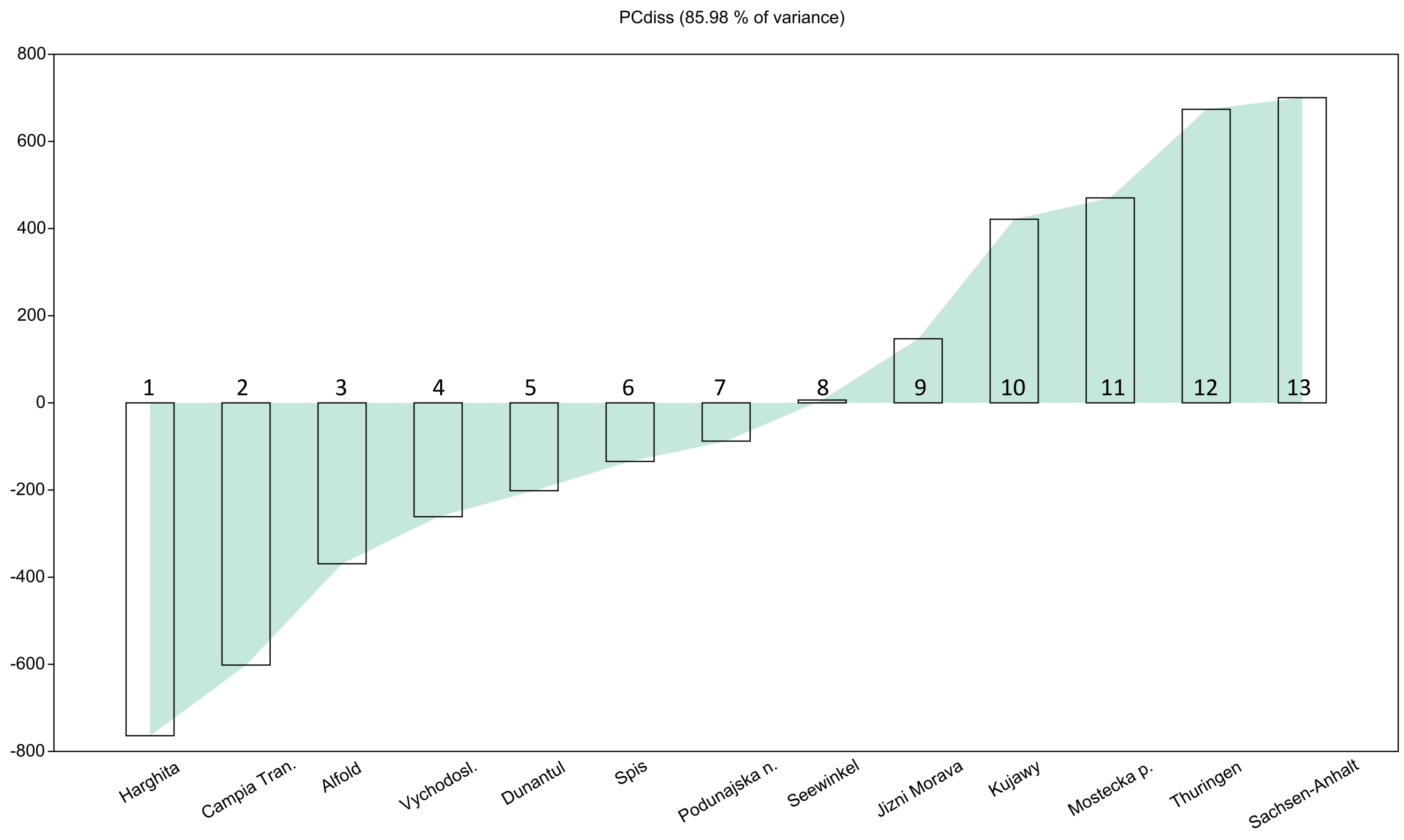
PCdiss (85.98 % of variance)
800
600
400
200
0
-200
-400
-600
-800
1
2
3
4
5
6
7
8
9
10
11
12
13
Harghita
Campia Tran.
Alfold
Vychodosl.
Dunantul
Spis
Podunajska n.
Seewinkel
Jizni Morava
Kujawy
Mostecka p.
Thuringen
Sachsen-Anhalt

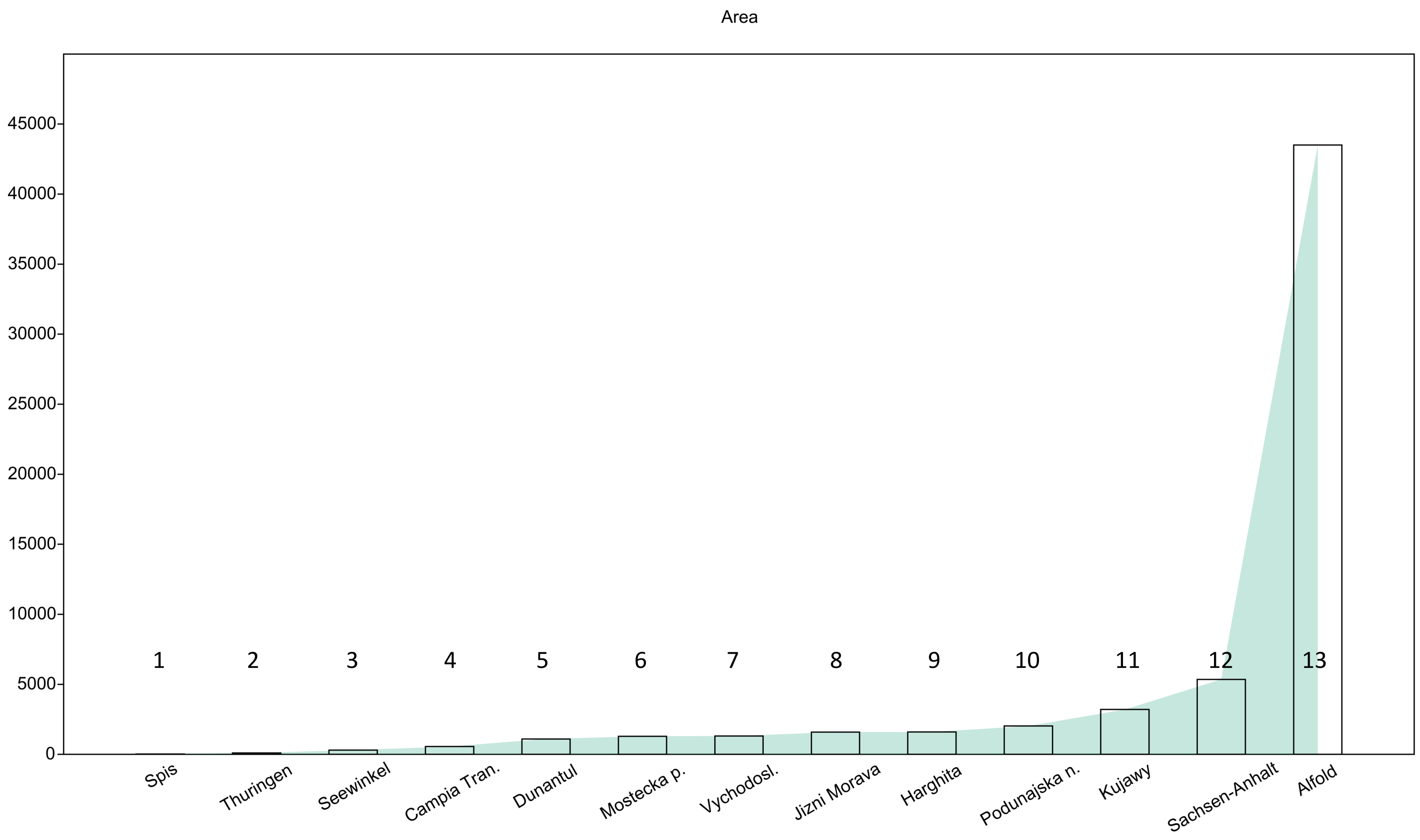

Area
45000
40000
35000
30000
25000
20000
15000
10000
5000
0
1
2
3
4
5
6
7
8
9
10
11
12
13
Spis
Thuringen
Seewinkel
Campia Tran.
Dunantul
Mostecka p.
Vychodosl.
Jizni Morava
Harghita
Podunajska n.
Kujawy
Sachsen-Anhalt
Alfold

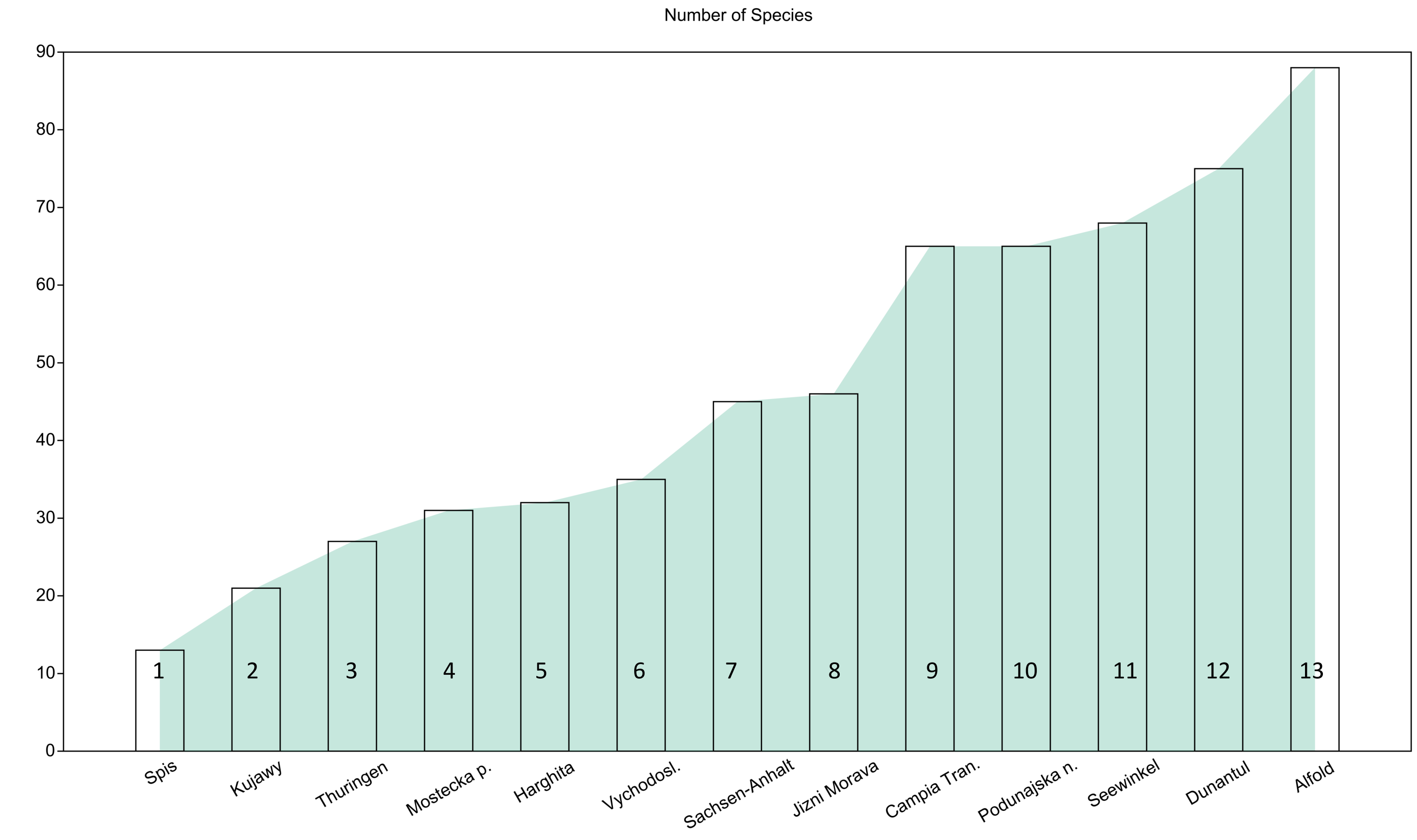

Number of Species
90
80
70
60
50
40
30
20
10
0
1
2
3
4
5
6
7
8
9
10
11
12
13
Spis
Kujawy
Thuringen
Mostecka p.
Harghita
Vychodosl.
Sachsen-Anhalt
Jizni Morava
Campia Tran.
Podunajska n.
Seewinkel
Dunantul
Alfold
